# Assessing the performance of population adjustment methods for anchored indirect comparisons: A simulation study

**DOI:** 10.1002/sim.8759

**Published:** 2020-10-04

**Authors:** David M. Phillippo, Sofia Dias, A. E. Ades, Nicky J. Welton

**Affiliations:** ^1^ Bristol Medical School (Population Health Sciences) University of Bristol Bristol UK; ^2^ Centre for Reviews and Dissemination University of York York UK

**Keywords:** effect modification, indirect comparison, individual patient data, matching‐adjusted indirect comparison, multilevel network meta‐regression, simulated treatment comparison

## Abstract

Standard network meta‐analysis and indirect comparisons combine aggregate data from multiple studies on treatments of interest, assuming that any factors that interact with treatment effects (effect modifiers) are balanced across populations. Population adjustment methods such as multilevel network meta‐regression (ML‐NMR), matching‐adjusted indirect comparison (MAIC), and simulated treatment comparison (STC) relax this assumption using individual patient data from one or more studies, and are becoming increasingly prevalent in health technology appraisals and the applied literature. Motivated by an applied example and two recent reviews of applications, we undertook an extensive simulation study to assess the performance of these methods in a range of scenarios under various failures of assumptions. We investigated the impact of varying sample size, missing effect modifiers, strength of effect modification and validity of the shared effect modifier assumption, validity of extrapolation and varying between‐study overlap, and different covariate distributions and correlations. ML‐NMR and STC performed similarly, eliminating bias when the requisite assumptions were met. Serious concerns are raised for MAIC, which performed poorly in nearly all simulation scenarios and may even increase bias compared with standard indirect comparisons. All methods incur bias when an effect modifier is missing, highlighting the necessity of careful selection of potential effect modifiers prior to analysis. When all effect modifiers are included, ML‐NMR and STC are robust techniques for population adjustment. ML‐NMR offers additional advantages over MAIC and STC, including extending to larger treatment networks and producing estimates in any target population, making this an attractive choice in a variety of scenarios.

## 1 INTRODUCTION

**FIGURE 1:**
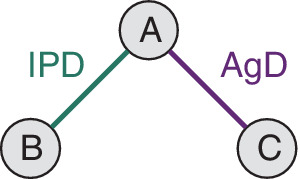
The two‐study indirect comparison scenario with one *A* vs *B* and one *A* vs *C* study. IPD are available for the *AB* study; only AgD are available for the *AC* study. An indirect comparison compares treatments *B* and *C* via the common *A* arm [Colour figure can be viewed at wileyonlinelibrary.com]

Indirect comparison and network meta‐analysis (NMA) are standard approaches for producing pooled estimates of relative treatment effects for multiple treatments from multiple randomized controlled trials, where the treatments of interest may not have all been compared in the same randomized controlled trial, but instead form a connected network of treatment comparisons.^1^, ^2^, ^3^, ^4^, ^5^, ^6^ For example, we may have a scenario (illustrated in Figure 1) where trials of treatments *A* vs *B* and *A* vs *C* are available, but there are no trials comparing *B* vs *C*. An indirect comparison for the *B* vs *C* treatment effect can be obtained via the common comparator *A*. NMA is the generalization of indirect comparisons to more complex networks of treatment comparisons. Health technology assessments, such as those conducted by the National Institute for Health and Care Excellence in the United Kingdom, require companies to submit evidence on the clinical and cost effectiveness of their treatment compared with other relevant treatments. Indirect comparisons and NMA are routinely used as part of the health technology assessment process, since the available trial evidence may not necessarily include a single head‐to‐head study of all of the relevant treatment options, and is likely to instead consist of several studies each comparing a subset of treatments.

Because the statistical methods used for indirect comparisons and NMA respect the randomization in the included trials, results are not affected by any differences between trials in the distribution of prognostic variables—those that affect absolute outcomes regardless of treatment. However, bias will be introduced if there are any differences between trials in the distribution of effect modifying variables—those that alter the relative effect of treatment as measured on a given scale. If individual patient data (IPD) are available from every study then an IPD network meta‐regression is the “gold‐standard” approach to adjusting for differences in effect modifiers.^7^, ^8^, ^9^, ^10^, ^11^ However, this level of data availability is rare in health technology appraisal: it is much more common for a submitting company to have IPD from their own study or studies, and only published aggregate data (AgD) from their competitors'. Several methods have been proposed in this scenario where there are studies with IPD and studies with AgD, aiming to use available IPD to produce a “population‐adjusted” indirect comparison.

Matching‐adjusted indirect comparison (MAIC)^12^, ^13^ and simulated treatment comparison (STC)^13^, ^14^ are population adjustment methods designed specifically for a two‐study indirect comparison scenario. In this scenario there is a single IPD study comparing treatments *A* and *B*, and a single AgD study comparing treatments *A* and *C* (Figure 1). MAIC is a weighting approach, and STC is based on regression adjustment. The relative effect of treatment *B* vs treatment *A* in the aggregate study population is predicted, based on weighting (for MAIC) or regression (for STC), and used to construct a population‐adjusted indirect comparison with the aggregate relative effect of *C* vs *A*.

Multilevel network meta‐regression (ML‐NMR) is an extension of the NMA framework to incorporate both individual and aggregate data from a connected network formed by any number of studies and treatments.^15^, ^16^ In contrast to previous approaches based on NMA where mean covariate values are “plugged in” to an individual‐level model for those studies with AgD,^17^, ^18^, ^19^, ^20^, ^21^ ML‐NMR instead integrates an individual‐level model over the covariate distribution in each AgD study, thus avoiding aggregation bias when the model is nonlinear.

Population adjustment methods are increasingly being used in health technology appraisal submissions and in the wider literature,^22^, ^23^, ^24^ yet to date have largely only been investigated in limited simulation studies designed to demonstrate performance under ideal scenarios where they would be expected to perform well. ML‐NMR, as the most recently published of the three methods, has yet to be examined in a simulation study. In this article, we aim to assess the performance of ML‐NMR, MAIC, and STC population adjustment methods in a range of ideal and nonideal scenarios under various failures of assumptions using a simulation study. We base our simulations on the two‐study setting presented in Figure 1. The simulation scenarios are designed to probe the underlying assumptions and properties of the methods,^22^, ^23^ and are motivated by recent reviews of technology appraisals submitted to the National Institute of Health and Care Excellence in the United Kingdom and of applications in the literature.^22^, ^25^

We begin in Section 2 by describing the methods of standard indirect comparison, MAIC, STC, and ML‐NMR. This is followed in Section 3 by a motivating example of population adjustment applied to a network of plaque psoriasis treatments. Section 4 then provides an overview of the assumptions, properties, and issues often encountered in practical applications of population adjustment methods, based on recent methodological^22^, ^23^ and applied^22^, ^25^ reviews. Motivated by the example and these reviews, we set out the simulation study design in Section 5, and report the results in Section 6. We conclude with a discussion in Section 7.

## 2 METHODS FOR POPULATION ADJUSTMENT

Indirect comparisons follow the same general form, regardless of the method used to estimate them. In a population *P*, the relative effect *d*_*BC*(*P* )_ of treatment *C* vs treatment *B* is determined from the difference in the relative effects of *B* and *C* against treatment *A*:

(1) $$d_{BC(P)}=d_{AC(P)}-d_{AB(P)}.$$

Differences between methods are due to how the relative effects *d*_*AB*(*P* )_ and *d*_*AC*(*P* )_ are estimated, and in which population. Equation (1) describes an *anchored* indirect comparison, since the comparison is made via the common comparator *A*. We focus on anchored indirect comparisons in this article. However, it is also possible to produce *unanchored* comparisons, without reliance on a common comparator, under much stronger assumptions (see Section 4.1 and the discussion).

### 2.1 Standard indirect comparison

A standard indirect comparison, without any population adjustment, is performed following the Bucher method.^1^ The relative effects are assumed to be constant across populations, so that the relative effect of *B* vs *A* in the population of the *AB* study is the same in the population of the *AC* study, *d*_*AB*(*AB*)_ = *d*_*AB*(*AC*)_ = *d*_*AB*_ , and similarly for the *C* vs. *A* effect *d*_*AC*(*AB*)_ = *d*_*AC*(*AC*)_ = *d*_*AC*_ , and the *C* vs *B* effect *d*_*BC*(*AB*)_ = *d*_*BC*(*AC*)_ = *d*_*BC*_ . The standard indirect comparison is then

(2a) $$\begin{array}{ll} {\hat{d}}_{BC} & ={\hat{d}}_{AC}-{\hat{d}}_{AB} \end{array}$$

with standard error

(2b) $$\begin{array}{ll} s_{BC} & =\sqrt{s_{AC}^{2}+s_{AB}^{2}}, \end{array}$$

where ${\hat{d}}_{AB}$ and ${\hat{d}}_{AC}$ are the relative effects estimated from the *AB* and *AC* studies respectively with standard errors *s*_*AB*_ and *s*_*AC*_ . Standard indirect comparisons will be biased if there are differences between studies in the distribution of effect‐modifying covariates.

### 2.2 Simulated treatment comparison

For STC, a regression model is fitted to the IPD in the *AB* study, with linear predictor

(3) $$g(\theta_{ik(AB)})=\mu_{\left(AB\right)}+x_{ik(AB)}^{T}\beta_{1}+\left(x_{ik(AB)}^{T}\beta_{2,B}+\gamma_{B}\right)𝕀(k=B),$$

where *g*(·) is a suitable link function (eg, the logit function for logistic regression) transforming the expected outcome $\theta_{ik(AB)}$ for individual *i* on treatment *k* in the *AB* study with covariate vector ***x***_*ik*(*AB*)_ , $\mu_{\left(AB\right)}$ is the intercept, $\beta_{1}$ are coefficients for prognostic variables, $\beta_{2,B}$ are coefficients for effect modifier interactions with treatment *B*, and $\gamma_{B}$ is the individual level effect of treatment *B* vs *A* at the reference level of the covariates. The predicted average treatment effect of *B* vs *A* in the *AC* population is then given by

(4) $${\hat{d}}_{AB(AC)}={\bar{x}}_{\left(AC\right)}^{T}{\hat{\beta }}_{2,B}+{\hat{\gamma }}_{B},$$

where ${\bar{x}}_{\left(AC\right)}$ is the vector of mean covariate values in the *AC* study population. A population‐adjusted indirect comparison between treatments *B* and *C* in the *AC* study population is then estimated as

(5a) $$\begin{array}{ll} {\hat{d}}_{BC(AC)} & ={\hat{d}}_{AC(AC)}-{\hat{d}}_{AB(AC)} \end{array}$$

with standard error

(5b) $$\begin{array}{ll} s_{BC(AC)} & =\sqrt{s_{AC(AC)}^{2}+s_{AB(AC)}^{2}}, \end{array}$$

where ${\hat{d}}_{AC(AC)}$ is the relative effect estimate from the *AC* study with standard error *s*_*AC*(*AC*)_ , and *s*_*AB*(*AC*)_ is the standard error of ${\hat{d}}_{AB(AC)}$ from the regression. STC produces estimates which are specific to the *AC* study population. However, making the shared effect modifier assumption (which is investigated in simulation scenario c, Section 6.3) means that the estimated *C* vs *B* relative effect is constant, *d*_*BC*(*AB*)_ = *d*_*BC*(*AC*)_ = *d*_*BC*_ , and thus applicable in any target population. STC is designed with this two‐study indirect comparison scenario in mind, and does not easily generalize to larger networks of studies or treatments.

### 2.3 Matching‐adjusted indirect comparison

MAIC estimates weights *w*_*ik*_ for each individual in the *AB* study, which match the moments of the reweighted covariate distributions to those in the *AC* study.^12^ Weights are estimated using the method of moments^12^ or entropy balancing,^26^, ^27^, ^28^ which have been shown to be equivalent.^29^ Given the weights, the predicted mean outcomes on treatment *k* = *A*, *B* in the *AC* population are estimated by taking a weighted average of the outcomes *y*_*ik*(*AB*)_ of the *N*_*k*(*AB*)_ individuals on treatment *k* in the *AB* population

(6) $${ŷ}_{k(AC)}=\frac{\sum_{i=1}^{N_{k(AB)}}y_{ik(AB)}w_{ik}}{\sum_{i=1}^{N_{k(AB)}}w_{ik}},$$

and the predicted average treatment effect of *B* vs *A* in the *AC* population is then given by

(7) $${\hat{d}}_{AB(AC)}=g({ŷ}_{B(AC)})-g({ŷ}_{A(AC)}).$$

The population‐adjusted indirect comparison between treatments *B* and *C* in the *AC* study population is again given by (5). Typically both the mean and variance of each continuous covariate are matched between the two study populations (only proportions are matched for discrete covariates), and the standard error *s*_*AB*(*AC*)_ is estimated using either bootstrapping or robust sandwich estimators.^12^, ^22^ MAIC produces estimates which are specific to the *AC* study population. Again however, making the shared effect modifier assumption means that the estimated *C* vs *B* relative effect is constant across populations. Like STC, MAIC is designed with this two‐study indirect comparison scenario in mind, and does not easily generalize to larger networks of studies or treatments.

### 2.4 Multilevel network meta‐regression

ML‐NMR extends the network meta‐analysis framework to synthesize evidence from a connected network of studies where some studies provide IPD and some provide AgD. The general ML‐NMR model is^16^

Individual level:

(8a) $$\begin{array}{ll} y_{ijk} & ∼\pi_{\text{Ind}}(\theta_{ijk}) \end{array}$$

(8b) $$\begin{array}{ll} g(\theta_{ijk}) & =\eta_{jk}(x_{ijk})=\mu_{j}+x_{ijk}^{T}(\beta_{1}+\beta_{2,k})+\gamma_{k} \end{array}$$

Aggregate level:

(8c) $$\begin{array}{ll} y_{•jk} & ∼\pi_{\text{Agg}}(\theta_{•jk}) \end{array}$$

(8d) $$\begin{array}{ll} \theta_{•jk} & =\int_{X}g^{-1}\left(\eta_{jk}(x)\right)f_{jk}(x)dx \end{array}$$

The individual‐ and aggregate‐level data are given appropriate likelihood distributions $\pi_{\text{Ind}}(\cdot )$ and $\pi_{\text{Agg}}(\cdot )$. $\theta_{ijk}$ and $\eta_{jk}(x_{ijk})$ are the conditional mean outcome and linear predictor for an individual *i* in trial *j* on treatment *k* with covariate vector ***x***_*ijk*_ . $\theta_{•jk}$ is the marginal mean outcome on treatment *k* in trial *j*. *g*(·) is a suitable link function, and X denotes the support of ***x*** . The coefficients $\mu_{j}$ are study‐specific baselines, $\beta_{1}$ are coefficients for prognostic variables, and $\beta_{2,k}$ are coefficients for effect modifiers specific to each treatment *k*. The effect of the *k*th treatment (at the individual level), $\gamma_{k}$, is defined with respect to the reference treatment *A*, and we set $\gamma_{A}=0$ and $\beta_{2,A}=0$. The shared effect modifier assumption is not required for ML‐NMR if sufficient data are available to identify the model parameters (eg, in larger networks); however, in a two‐study indirect comparison scenario we make the shared effect modifier assumption setting $\beta_{2,B}=\beta_{2,C}$ to identify the model.

Numerical integration may be used to evaluate the integral (8d) for the aggregate‐level model. The quasi‐Monte Carlo integration approach described by Phillippo et al^15^, ^16^ is flexible, efficient, and widely applicable regardless of the model form or complexity. However, this approach typically requires assumptions regarding the forms of the marginal distributions and the covariate correlations in the AgD study or studies, as these are rarely reported in publications (Section 4.5). These assumptions are investigated in simulation scenarios g, h, and i (Section 6.6).

Using ML‐NMR, the population‐adjusted average treatment effects in each study population *P* = *AB* , *AC* are given by

(9) $$d_{ab(P)}={\bar{x}}_{\left(P\right)}^{T}(\beta_{2,b}-\beta_{2,a})+\gamma_{b}-\gamma_{a},$$

where *a*, *b* ∈ {*A*, *B*, *C*} and ${\bar{x}}_{\left(P\right)}$ is the vector of covariate means in population *P*.

Unlike MAIC or STC, ML‐NMR extends naturally to larger networks: Equation (8) applies equally to networks with any number of treatments and any number of AgD or IPD studies. Moreover, through Equation (9) we can produce population‐adjusted treatment effects for any target population with given covariate values, not just the AgD *AC* study population. Notably the ML‐NMR model (8) reduces to standard AgD NMA when no covariates are included in the model, and reduces to IPD network meta‐regression when IPD are available from every study.^16^

## 3 EXAMPLE: PLAQUE PSORIASIS

**FIGURE 2:**
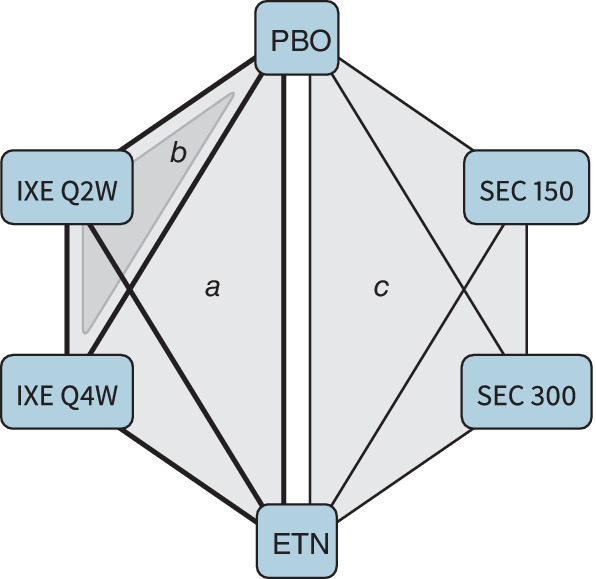
The UNCOVER^30^, ^31^ and FIXTURE^32^ trials form a network of six treatments. Shading indicates comparisons made in: (*a*) UNCOVER‐2 and UNCOVER‐3; (*b*) UNCOVER‐1; (*c*) FIXTURE. The thick and thin lines represent availability of IPD and AgD on a comparison, respectively. ETN, etanercept; IXE, ixekizumab; PBO, placebo; SEC, secukinumab. IXE and SEC were each investigated with two different dosing regimens. Reproduced from Phillippo et al^16^ [Colour figure can be viewed at wileyonlinelibrary.com]

We now demonstrate the application of MAIC, STC, and ML‐NMR to a real example. Figure 2 displays the treatment network formed by four phase 3 trials comparing six treatments for moderate‐to‐severe plaque psoriasis. In UNCOVER‐1, UNCOVER‐2, and UNCOVER‐3, patients were randomized to receive placebo, etanercept (in UNCOVER‐2 and UNCOVER‐3 only), ixekizumab every 2 weeks (Q2W), or ixekizumab every 4 weeks (Q4W).^30^, ^31^ In FIXTURE, patients were randomized to receive placebo, secukinumab 150 mg, secukinumab 300 mg, or etanercept.^32^ IPD were only available from UNCOVER‐1, UNCOVER‐2, and UNCOVER‐3. We analyze a binary outcome, success/failure to achieve 75% improvement on the Psoriasis Area and Severity Index (PASI) scale (PASI 75) at 12 weeks, on the standardized mean difference (SMD) scale (ie, using a probit link function). Five clinically relevant covariates considered to be potential effect modifiers are available for individuals in the UNCOVER trials and as summary statistics from the FIXTURE trial, with some differences between studies in the distribution of these covariates: mean body surface area covered ranges from 26.0% to 34.4%; mean duration of psoriasis ranges from 16.5 to 19.6 years; the proportion of individuals having previous systemic treatment ranges from 57.1% to 71.3%; the proportion of individuals with psoriatic arthritis ranges from 14.7% to 26.3%; and mean weight ranges from 83.3 to 92.2 kg. The within‐study variation in each covariate is much greater than the variation between studies, with standard deviations between 16.5% and 18.9% for body surface area, 11.9 to 12.5 years for duration of psoriasis, and 20.8 to 23.8 kg for weight. Full details are given by Phillippo et al.^16^

MAIC has previously been used to estimate a population‐adjusted indirect comparison between ixekizumab Q2W and secukinumab 300 mg (the approved dosages), adjusting for these baseline covariates, via the common etanercept arms in UNCOVER‐2 and UNCOVER‐3 and FIXTURE.^16^, ^33^ We also estimate this comparison using STC. However, for both MAIC and STC, data from UNCOVER‐1 could not be included as this study did not include an etanercept arm. Furthermore, comparisons made using MAIC and STC are valid only for the population of the study with AgD (here FIXTURE) unless the shared effect modifier assumption is made (Sections 2.2 and 2.3).

Using ML‐NMR allows all of the available data to be incorporated and to produce population‐adjusted indirect comparisons between any pair of treatments in any chosen target population. We use quasi‐Monte Carlo numerical integration to implement the aggregate‐level model, integrating the individual‐level model (8b) over the covariate distribution in the FIXTURE trial.^16^ The forms of the marginal distributions for each covariate in the FIXTURE trial are chosen based on theoretical properties and on the observed distributions in the UNCOVER trials: weight and duration are given a Gamma distribution to account for skewness, and body surface area as a percentage is given a scaled logit‐Normal distribution. Previous systemic treatment and psoriatic arthritis are binary covariates. Correlations between covariates in the FIXTURE study are assumed to match those observed in the UNCOVER trials since these are not reported, and are accounted for in the numerical integration using a Gaussian copula.^16^

We then fit a ML‐NMR model to the PASI 75 outcomes, including interaction terms for the five potential effect modifiers. We take a Bayesian approach implemented in Stan,^34^ placing a noninformative N(0, 100^2^) prior distribution on each parameter. With only two AgD secukinumab treatment arms available, it is not possible to identify a model with five distinct effect modifier interactions and a treatment effect for each secukinumab dose. However, since secukinumab and ixekizumab share modes of action as interleukin‐17A blockers, we assume that the effect modifier interaction parameters are common between these treatments across all doses (the shared effect modifier assumption) to identify the model. Four parallel chains were run for 2000 iterations each (the first 1000 iterations were discarded as warm‐up). Convergence was assessed using the potential scale reduction factor $\hat{R}$,^35^ and effective sample sizes and Monte Carlo standard errors guided the choice of number of iterations. There were no divergent transitions. Full details of the analysis are given by Phillippo et al.^16^

The treatment contrast estimates for MAIC, STC, and ML‐NMR are summarized in Table 1. There are small differences in the estimated average treatment effects in each population, for example, etanercept appears slightly more effective relative to placebo in the FIXTURE study population than in the UNCOVER study populations. Using MAIC, the comparison between ixekizumab Q2W and secukinumab 300 mg in the FIXTURE population is estimated as a SMD of 0.28 (0.00, 0.56) in favor of ixekizumab Q2W; with STC, this comparison estimate is 0.25 (−0.02, 0.52); with ML‐NMR, we estimate 0.34 (0.10, 0.58). The standard indirect comparison estimate is 0.37 (0.12, 0.63). The point estimates are similar between the three population adjustment approaches, but ML‐NMR has reduced uncertainty compared with MAIC and STC due to incorporating all available information from the studies. Phillippo et al^16^ also fitted a standard random effects NMA to this data, finding that ML‐NMR also reduced uncertainty compared with the NMA. For example, the random effects NMA estimate for the comparison between ixekizumab Q2W and secukinumab 300 mg was 0.45 (−0.02, 0.92). Since the differences in effect modifiers between trials are small, the possible bias in a standard indirect comparison or NMA is likely to also be small; however, ML‐NMR increases the precision of the estimates by explaining the within‐trial variation due to effect modification.

**TABLE 1:** ML‐NMR estimated SMD contrasts and 95% credible intervals for each treatment against placebo, plus the contrast targeted MAIC and STC, in each study population

	Study population			
Contrast	FIXTURE	UNCOVER‐1	UNCOVER‐2	UNCOVER‐3
IXE Q2W vs PBO	2.94	2.98	2.95	2.93
	(2.74, 3.14)	(2.80, 3.17)	(2.77, 3.13)	(2.76, 3.11)
IXE Q4W vs PBO	2.65	2.69	2.66	2.64
	(2.45, 2.84)	(2.51, 2.89)	(2.47, 2.84)	(2.46, 2.82)
ETN vs PBO	1.74	1.65	1.64	1.65
	(1.55, 1.93)	(1.47, 1.83)	(1.46, 1.81)	(1.47, 1.81)
SEC 150 vs PBO	2.29	2.33	2.30	2.28
	(2.07, 2.53)	(2.10, 2.58)	(2.07, 2.54)	(2.05, 2.52)
SEC 300 vs PBO	2.60	2.64	2.61	2.59
	(2.36, 2.83)	(2.40, 2.90)	(2.36, 2.86)	(2.35, 2.83)
SEC 300 vs IXE Q2W	−0.34^a^	−0.34	−0.34	−0.34
	(−0.58, −0.10)	(−0.58, −0.10)	(−0.58, −0.10)	(−0.58, −0.10)

*Note*: The ML‐NMR contrast estimates between ixekizumab and secukinumab treatments are the same in every population due to the shared effect modifier assumption for these treatments.

^a^ MAIC estimate is −0.28 (−0.56, −0.00). STC estimate is −0.25 (−0.52, 0.02). Standard indirect comparison estimate is −0.37 (−0.63, 0.12).

This example raises a number of issues which we will investigate in the simulation study. First, we made explicit assumptions regarding the joint covariate distribution in the FIXTURE study for ML‐NMR, and MAIC and STC also make implicit assumptions about the joint covariate distribution (see Section 4.5). The shared effect modifier assumption was required to identify the ML‐NMR model, and is also necessary if the results of MAIC and STC are to be applicable to a target population other than the FIXTURE study. While this assumption seems reasonable in this example since ixekizumab and secukinumab are in the same class of treatments, this may not always be the case. For all three population adjustment methods we assume that all effect modifiers have been suitably adjusted for; however, there may be unobserved or omitted effect modifiers. Finally, while the differences in covariate distributions between study populations were relatively minor, in many cases there may be more substantial differences (see Section 4.7), which raises questions regarding sufficient between‐study overlap and validity of extrapolation, particularly with continuous covariates. We would like to investigate the potential impact of each of these assumptions, particularly as they are often unverifiable (although some validation may be possible in larger networks using ML‐NMR, see Section 7).

## 4 ASSUMPTIONS AND ISSUES IN PRACTICAL APPLICATIONS OF POPULATION ADJUSTMENT

We now provide a more detailed overview of the assumptions, properties, and issues often encountered in practical applications of population adjustment methods, based on recent methodological^22^, ^23^ and applied^22^, ^25^ reviews.

### 4.1 Anchored indirect comparisons

Anchored indirect comparisons make use of a common comparator treatment to compare relative effects (Figure 1). That is, in a population *P*, the relative effect *d*_*BC*(*P* )_ of treatment *C* vs treatment *B* is determined from the difference in the relative effects of *B* and *C* against treatment *A* following Equation (1). Standard indirect comparisons make the *constancy of relative effects* assumption, which requires that there are no differences in the distribution of any effect modifiers between studies, so that the relative effects in (1) are constant across study populations. Anchored population adjustment methods relax this assumption to *conditional constancy of relative effects*, that is, all of the effect modifiers with respect to the chosen comparison scale (eg, log odds ratios) that are imbalanced between studies have been suitably adjusted for.^22^, ^23^

Unanchored indirect comparisons may be performed when there is no common comparator between randomized controlled trials or if a comparison involves single arm studies, under much stronger assumptions.^22^, ^23^ We focus on an anchored scenario in the simulation study (see Section 5.2), but expect the results and conclusions to apply widely to unanchored comparisons also. We discuss this further in Section 7.

### 4.2 Covariate selection and unobserved effect modifiers

For anchored indirect comparisons, we are concerned with identifying and adjusting for effect‐modifying covariates, so that the conditional constancy of relative effects assumption holds. Potential effect modifiers should be identified prior to analysis;^22^, ^23^ however, in practical applications there has often been no justification of effect modifier status, or any attempt to check for effect modifiers that are missing from the model (either omitted or unobserved).^22^, ^25^ In the plaque psoriasis example we adjusted for five covariates that had been selected in a previous analysis by expert opinion,^33^ and assumed that relative effects were constant given these (Section 3). We therefore assess the performance of the three population adjustment methods when all effect modifiers are accounted for and when there is a missing effect modifier, across all simulation scenarios. We also compare performance against a standard indirect comparison that makes no adjustment for effect modifiers.

### 4.3 Combining compatible estimates

When considering noncollapsible measures of treatment effects such as odds ratios or hazard ratios, care must be taken to ensure that compatible estimates that have been adjusted in the same way are combined. This is because, for such effect measures, adjustment for a covariate moves the treatment effect estimate away from the null and adjusted and unadjusted estimands do not typically coincide, even without interaction or effect modification.^36^, ^37^ Standard indirect comparison and MAIC typically combine unadjusted treatment effects (eg, crude log odds ratios). However, STC as commonly implemented^22^ combines an adjusted treatment effect estimate (from the STC model) with an unadjusted estimate from the *AC* study, which may lead to bias. An adjusted estimate from the *AC* study may be used instead, but it is unlikely that this estimate has been adjusted in the same manner as the STC model, if an adjusted estimate is even available. ML‐NMR correctly combines adjusted and unadjusted effect measures at each level of the model, through the integration in Equation (8d). Network meta‐regression with full IPD (the “gold‐standard” approach) combines adjusted treatment effects from each study, so we consider adjusted population‐average treatment effects as the target estimands of interest in this simulation study (Section 5.3). We discuss this issue and the interpretation of our results further in Section 7.

### 4.4 Producing estimates for a chosen target population

When effect modification is present, relative effect estimates are population specific. What is required for health technology assessment are estimates in the population that represents the target population for making decisions.^22^ For MAIC and STC, the resulting population‐adjusted estimates are valid in the population of the *AC* study—which is often not representative of the decision at hand, as found by the review of technology appraisals.^25^ The *shared effect modifier* assumption is required to generalize the resulting relative effect estimate to other target populations, and means that the effect modifiers of the active treatments are the same and interact with each treatment in the same way.^22^, ^23^ In the plaque psoriasis example (Section 3), this assumption was required for the MAIC and STC estimates to be applicable to a target population other than the FIXTURE study. This assumption may also be required to identify the ML‐NMR model in smaller networks, as demonstrated in the plaque psoriasis example where effect modifier coefficients were shared between ixekizumab and secukinumab. While this assumption seems reasonable in this example since ixekizumab and secukinumab are in the same class of treatments, this may not always be the case. We therefore investigate the impact of making this assumption when it does not hold. Using ML‐NMR in a larger network may allow this assumption to be assessed or relaxed; however, this is not possible using any of the approaches in the two‐study scenario, where the assumption is unverifiable.^16^

### 4.5 Availability of the joint covariate distribution

The joint distribution of covariates is not typically published, instead only marginal covariate summaries are available (eg, mean and standard deviation for continuous covariates, proportions for categorical covariates). However, for ML‐NMR the covariate joint distribution in the AgD studies is required in order to perform the integration step. Thus when implementing ML‐NMR, assumptions regarding the marginal distributional forms and the correlation structure are typically required to construct a joint distribution from the marginal summaries.^16^ In the plaque psoriasis example, we used IPD from the UNCOVER studies to guide the choice of marginal distributional forms and to compute a correlation matrix, to construct a joint distribution for the FIXTURE study (Section 3). This is a potential limitation of ML‐NMR, and we thus wish to assess the performance of ML‐NMR when incorrect assumptions regarding the covariate joint distribution are made. MAIC and STC typically ignore the correlations between covariates^22^, ^23^ which may in turn affect the performance of these methods, for example, if correlations differ between studies, and we also assess this in the simulation study.

### 4.6 Study sample size

In practical applications of population adjustment methods, sample sizes of the included studies are variable, ranging from several thousand down to less than a hundred participants.^22^, ^25^ We therefore assess performance of the methods at a range of sample sizes in this simulation study.

### 4.7 Overlap between populations

An important property of population adjustment methods is that they require sufficient overlap between the population of the IPD study and the AgD study.^22^, ^23^ As a weighting method MAIC cannot extrapolate, and thus requires that the AgD study population is contained within the IPD study population in order to effect an adjustment. However, considerable reductions in effective sample size after weighting in MAIC are often seen in practice. In the two reviews of applications, the average reduction in effective sample size was 70% in technology appraisals^25^ and 80% in the applied literature,^22^ and in some cases was as high as 98%. This indicates that in many cases there may be poor overlap between the study populations. The issue of overlap is also related to the choice of covariates to include in the adjustment. As the number of covariates increases the effective overlap between populations must decrease, a phenomenon that we observe later in the results of the simulation study (Section 6.1). To date, no simulation studies have investigated the performance of population adjustment methods as the level of overlap decreases. We therefore assess all methods at a range of levels of between‐study overlap. Regression‐based approaches such as STC and ML‐NMR are not restricted to scenarios with sufficient overlap, but any resulting extrapolation requires an additional assumption about the validity of predictions beyond the range of the IPD. We therefore also assess performance when extrapolation outside of the range of the IPD study is invalid.

## 5 SIMULATION STUDY PLAN

This simulation study plan follows the ADEMP framework,^38^ which breaks down the simulation study into five key elements: aims, data‐generating mechanisms, estimands, methods, and performance measures. The following sections are devoted to describing each of these elements in turn.

### 5.1 Aims

The simulation study aims to assess the performance of ML‐NMR, MAIC, and STC alongside standard indirect comparison in a range of ideal and nonideal scenarios under various failures of assumptions. The primary concerns are bias and efficiency of the estimators, along with coverage.

### 5.2 Data‐generating mechanisms

For this simulation study, we will consider a binary outcome, generated under a logit (log odds ratio) model. The basic data structure involves three treatments (*A*, *B*, *C*) investigated in two studies, *AB* and *AC* (Figure 1). We will simulate two continuous covariates which modify the effect of treatments *B* and *C*. IPD are available for the *AB* study as a binary outcome and covariate information for each individual. Only AgD are available for the *AC* study, given as an overall event count and summary covariate information. For the *AC* study, individual outcomes will be simulated and then aggregated.

The underlying model is of the form

$$\begin{array}{ll} y_{ijk} & ∼\text{Bern}(\theta_{ijk})\;\\ \text{logit}(\theta_{ijk}) & =\mu_{j}+q\left(x_{ijk}^{T}-m_{j}^{T}\right)\beta_{k}+\gamma_{k}\;\\ x_{ijk} & ∼\phi_{j},\; \end{array}$$

where outcomes *y*_*ijk*_ for individual *i* in study *j* receiving treatment *k* are generated from a Bernoulli distribution with individual event probability $\theta_{ijk}$. We define an outcome model on the logit scale with study intercept $\mu_{j}$, covariate vector ***x***_*ijk*_ and coefficients $\beta_{k}$, and treatment effects $\gamma_{k}$. The covariates are generated following the joint distribution $\phi_{j}$. The function *q*(·) defines a (potentially nonlinear) relationship between the covariates and outcome. Covariates are centered in the regression model against the mean in each study ***m***_*j*_ , so that the model coefficients are more easily interpreted as log odds and log odds ratios at the mean.

We set $\gamma_{A}=0$, $\gamma_{B}=-2$, $\gamma_{C}=-1.5$, $\mu_{AB}=1$, $\mu_{AC}=1.5$. The values of $\beta_{k}$ will be set depending on the scenarios set out below, but always $\beta_{A}=0$ because *A* is the reference treatment.

In the *AB* trial, the two covariates will be generated with means $m_{X_{1}(AB)}=1$ and $m_{X_{2}(AB)}=0.5$, and standard deviations $\sigma_{X_{1}(AB)}=0.5$ and $\sigma_{X_{2}(AB)}=0.1$ (as either a Normal or Gamma covariate, see g and h below). The distributions of the covariates in the *AC* trial are set to achieve the required overlap (see e below).

We shall consider varying the following parameters (where the reference levels for each parameter are **bold and underlined** below):

a. Overall sample size *N* in *AB* and *AC* trials (100, **500**, 1000). We will use 1:1 randomization within each study.
b. Strength of effect modification. Modifying the treatment effect log odds ratio by **0.1**, 0.5 per *AB* covariate SD, that is, $\beta_{k}=0.1\sigma_{X(AB)}$ or $0.5\sigma_{X(AB)}$. This corresponds to roughly ±10% and ±50% modification of treatment effects within ±2 SD of the mean covariate value ***m***_*X*(*AB*)_ in the *AB* study.
c. Shared effect modification. Effect modifier coefficients are **shared** ($\beta_{B}=\beta_{C}$) or not between treatments *B* and *C*.
d. Strength of correlation between covariates in each study ($\rho_{\left(AB\right)}=\rho_{\left(AC\right)}$ = 0, **0.25**, 0.5).
e. Between‐study overlap. Full overlap (AC contained entirely within *AB* ), and **50%**, 100% of *AC* population outside of *AB* . Set using a proxy parameter κ (see below).
f. Covariate‐outcome relationship. **Linear**, nonlinear beyond the range of the *AB* study.
g. Distribution of covariates in *AB* . Consider **Normal covariates** and Gamma covariates.
h. Distribution of covariates in *AC* . Consider **Normal covariates** and Gamma covariates.
i. Correlation structures. **Same in both studies** ($\rho_{\left(AB\right)}=\rho_{\left(AC\right)}$), different in each study ($\rho_{\left(AB\right)}\neq \rho_{\left(AC\right)}$).

For the purposes of computation time and reporting, we will not consider a full‐factorial design of all possible data‐generating mechanisms. Instead, each parameter will be varied independently. However, we will consider a factorial examination of scenarios e and f (investigating validity of extrapolation), and of g, h, and i (investigating validity of assumptions regarding the joint covariate distribution in the AgD study).

For scenario e, we wish to vary the overlap between the study populations, that is, the proportion of the AgD *AC* population lying within the IPD *AB* population (see Appendix A). We vary a proxy parameter κ, where approximately κ=0 corresponds to no overlap, κ=0.5 to 50% overlap, and κ=1 to full overlap. Using κ, we then define the mean and standard deviation of each covariate in the *AC* population as

$$\begin{array}{ll} m_{X(AC)} & =\left(1.1+{\left(1-\kappa \right)}^{2}\right)m_{X(AB)},\;\\ \sigma_{X(AC)} & =0.75\sigma_{X(AB)},\; \end{array}$$

noting that these are set to differ from the *AB* population even when κ=1.

**FIGURE 3:**
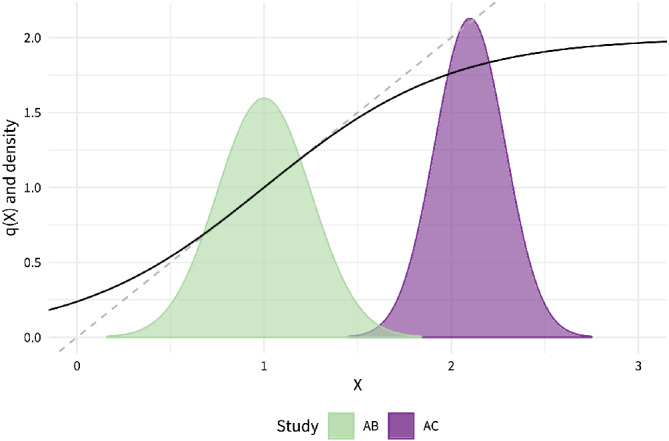
The function *q*(·) is approximately linear within the range of the *AB* study, but attenuates outside this range [Colour figure can be viewed at wileyonlinelibrary.com]

For scenario f, the nonlinear relationship will be specified with a sigmoid function, parametrized so that *y* ≈ *x* within ±2 SD of the mean covariate value in the *AB* study, but then attenuating outside this range (Figure 3):

(10) $$q(x)=8\sigma_{X(AB)}\left(\frac{1}{1+\exp(-(x-m_{X(AB)})/2\sigma_{X(AB)})}-\frac{1}{2}\right)+1.$$

### 5.3 Estimands

The estimands of interest will be adjusted population‐average relative effects between each treatment, as would be estimated by a “gold standard” correctly specified IPD network meta‐regression, with target population:

1. Represented by the *AC* trial population, denoted *d*_*AB*(*AC*)_ , *d*_*AC*(*AC*)_ , *d*_*BC*(*AC*)_ ;
2. Represented by the *AB* trial population, denoted *d*_*AB*(*AB*)_ , *d*_*AC*(*AB*)_ , *d*_*BC*(*AB*)_ .

ML‐NMR estimates all six of these relative effects (see Section 2.4). MAIC and STC only estimate *d*_*AB*(*AC*)_ , *d*_*BC*(*AC*)_ , and *d*_*BC*(*AB*)_ (see Sections 2.3 and 2.2), and so we only report results for these relative effects. For comparison, we report the same three relative effects for standard indirect comparison. For MAIC, STC, and standard indirect comparison, *d*_*AB*(*AB*)_ and *d*_*AC*(*AC*)_ are taken to be the observed relative effects in the *AB* and *AC* studies respectively and are thus unchanged between these methods.

### 5.4 Methods under evaluation

We compare ML‐NMR, MAIC, STC, and standard indirect comparison, as described above in Section 2. For ML‐NMR, MAIC, and STC we will consider models with a full set of effect modifiers or missing one effect modifier. For standard indirect comparison, no effect modifiers are adjusted for.

For MAIC, we match both the mean and variance of the covariates between the study populations and estimate the standard error *s*_*AB*(*AC*)_ using bootstrapping.^22^ For STC, we fit a logistic regression model to the IPD in the *AB* study, with linear predictor given by (3) and a logit link function. For both MAIC and STC, making the shared effect modifier assumption (which is investigated in scenario c, Section 6.3) means that the estimated *B* vs *C* relative effect is constant across populations, *d*_*BC*(*AB*)_ = *d*_*BC*(*AC*)_ = *d*_*BC*_ .

For ML‐NMR, we use a logit link function, a Bernoulli individual‐level likelihood $\pi_{\text{Ind}}(\theta_{ijk})=\text{Bern}(\theta_{ijk})$, and a Binomial aggregate‐level likelihood $\pi_{\text{Agg}}(\theta_{•jk})=\mathrm{Bin}(N_{jk},\theta_{•jk})$. In this two‐study scenario, we require the shared effect modifier assumption to identify the model: we set $\beta_{2,B}=\beta_{2,C}$. This assumption is investigated in scenario c (Section 6.3). We evaluate the integral (8d) for the aggregate‐level model using quasi‐Monte Carlo numerical integration, with a Gaussian copula to account for the correlations between covariates. In the AgD study we only have marginal covariate information (here, means and standard deviations); therefore, the joint covariate distribution *f*_(*AC*)_(***x***) in the AgD study is assumed to have the same form of marginal distributions and covariate correlations as in the IPD study, an assumption that we investigate in scenarios g, h, and i (Section 6.6).

We implement ML‐NMR in a Bayesian framework using Stan.^34^ We use vague N(0, 100^2^) prior distributions for all parameters, and summarize uncertainty in the parameter estimates with standard errors and credible intervals from the posterior distribution.

### 5.5 Performance measures

To assess the performance of each method, we compute the bias, empirical standard error, model standard error, and coverage probability in each simulation scenario. Bias is the difference between the expected value of an estimator and the truth, estimated as the average difference between the repeated estimates and the truth. An unbiased estimator (bias of zero) is desirable. The empirical standard error is the true variability of the estimator, estimated as the observed standard error of the repeated estimates. The model standard error is the average standard error reported by a method over all repetitions (taken on the variance scale). We thus desire both that the empirical standard error is small (the estimator is precise) and that the empirical standard error is well estimated by the model standard error (so that uncertainty is appropriately quantified). The coverage probability is the probability that the confidence or credible intervals contain the true value, estimated as the proportion of repetitions with intervals that include the true value, which we wish to be at the nominal level (here 95%).

## 6 RESULTS

The simulation study was run in R 3.4.1,^39^ using the package simsalapar ^40^ to facilitate parallelization, error handling, and saving of results. Each scenario was replicated 2000 times to achieve Monte Carlo standard errors (on the log odds ratio scale) below 0.035 for bias and 0.025 for empirical standard error, for all methods (other than a small number of scenarios for which MAIC was highly unstable).

Due to the size of simulation study, additional figures (including coverage plots) and tabulated summaries of results can be found in Appendix B. For reference, Table 2 summarizes the simulation scenarios and lists the corresponding results sections and appendices.

**TABLE 2:** Overview of simulation study scenarios and index of results

Scenario	Investigating	Results section	Appendix
a	Sample size	6.1	B.1
b	Strength of effect modification	6.2	B.2
c	Shared effect modifier assumption	6.3	B.3
d	Correlation between covariates	6.4	B.4
e and f	Between‐study overlap and covariate‐outcome relationship	6.5	B.5
g, h, and i	Covariate distributions and correlation structures	6.6	B.6

### 6.1 Scenario a: Sample size

**FIGURE 4:**
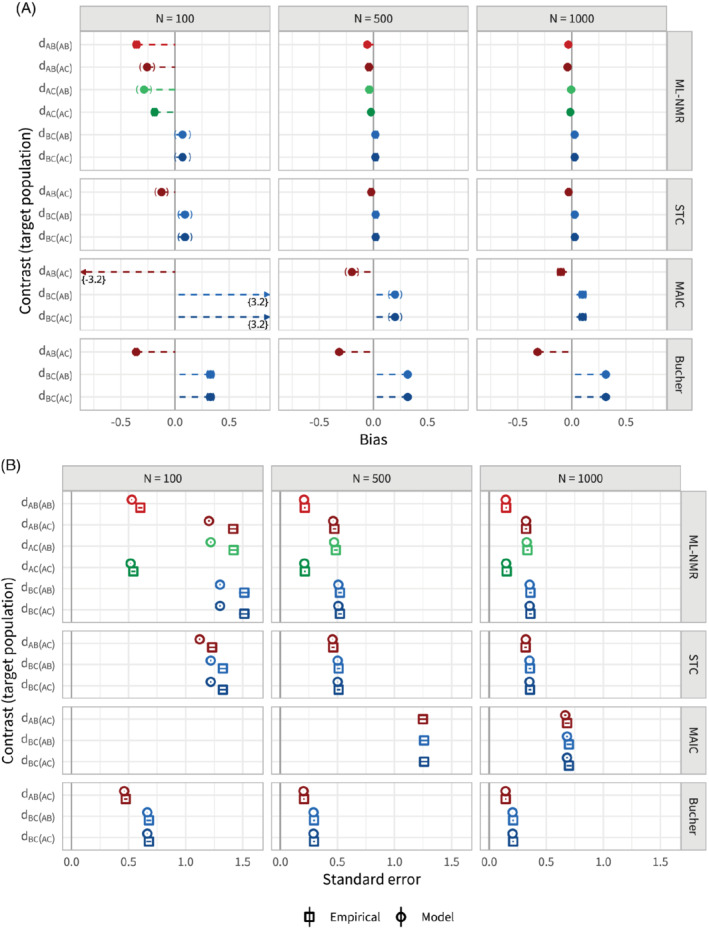
Bias, A, and standard errors, B, for the population‐average contrast estimates for scenario a, along with 95% Monte Carlo confidence intervals. Each method (other than Bucher) adjusts for the full set of effect modifiers. Sample size is varied between 100, 500, and 1000. The points are colored by contrast, with lighter shades for the *AB* population and darker for the *AC* population [Colour figure can be viewed at wileyonlinelibrary.com]

In scenario a, the sample sizes of the two studies were varied between 100, 500, and 1000. Figure 4A shows the bias in the population‐average contrast estimates for each method, adjusting for all effect modifiers, along with 95% Monte Carlo confidence intervals. To aid comparison between methods, the points are colored by contrast, with lighter shades for the *AB* population and darker for the *AC* population. Figure 4B shows the corresponding empirical and model standard errors. Coverage zip plots^38^ for the *d*_*BC*(*AC*)_ contrast estimate are shown in Figure B1. Table B1 provides the results in tabular format.

We see that ML‐NMR and STC are comparable in terms of both bias and standard error, largely eliminating the bias that the Bucher method (standard indirect comparison) incurs, with similar empirical standard errors that are well estimated by the model standard errors. For the smallest sample size (100), the model standard errors slightly underestimate the empirical standard errors. Some small bias remains in each of the estimates for ML‐NMR and STC, and is most pronounced at the smallest sample size; this is likely due to the small sample bias inherent to logistic regression and the low average number of events on treatment *B* (13.6 from 50 individuals).^41^ As expected, ML‐NMR produces more precise estimates (ie, with lower standard error) for contrasts between treatments where there is direct evidence than those based on indirect evidence ( *d*_*AB*(*AB*)_ and *d*_*AC*(*AC*)_ are more precisely estimated than the remaining contrasts). ML‐NMR and STC also perform similarly in terms of coverage, achieving nominal coverage for sample sizes 500 and 1000. Both methods display slight undercoverage at the smallest sample size (100), 90.6% (89.3, 91.8) for ML‐NMR, and 93.6% (92.6, 94.7) for STC, perhaps due to increased small‐sample bias. The standard indirect comparison is always below nominal coverage, and coverage drops off severely with increasing sample size as a biased estimator is more precisely estimated (note the rightward skew to the zip, which agrees with Figure 4A).

MAIC does not perform as well as ML‐NMR or STC in this scenario. The reference level of between‐study overlap is set at 50%, but this means that any reweighting scheme such as MAIC cannot hope to eliminate the bias since extrapolation is not possible. As a result, MAIC provides a lesser bias reduction than ML‐NMR and STC for sample sizes 500 and 100, and for the smallest sample size actually substantially increases the bias compared with a standard indirect comparison. Empirical standard errors for MAIC are larger than for ML‐NMR and STC, and the MAIC model standard error is only a good estimate at the largest sample size. For sample size 500, the MAIC model standard errors (derived through bootstrapping) are extremely unstable, as the weights are highly dependent on a small number of individuals, and were only successfully obtained 47% of the time. For sample size 100, estimation failed entirely 23% of the time, and no attempts to obtain model standard errors were successful. Coverage for sample size 500 is at the nominal value, despite remaining bias and unstable standard errors. For sample size 1000, coverage of 90.7% (89.4, 91.9) is below the nominal value, as the standard errors have stabilized but the estimator remains biased.

**FIGURE 5:**
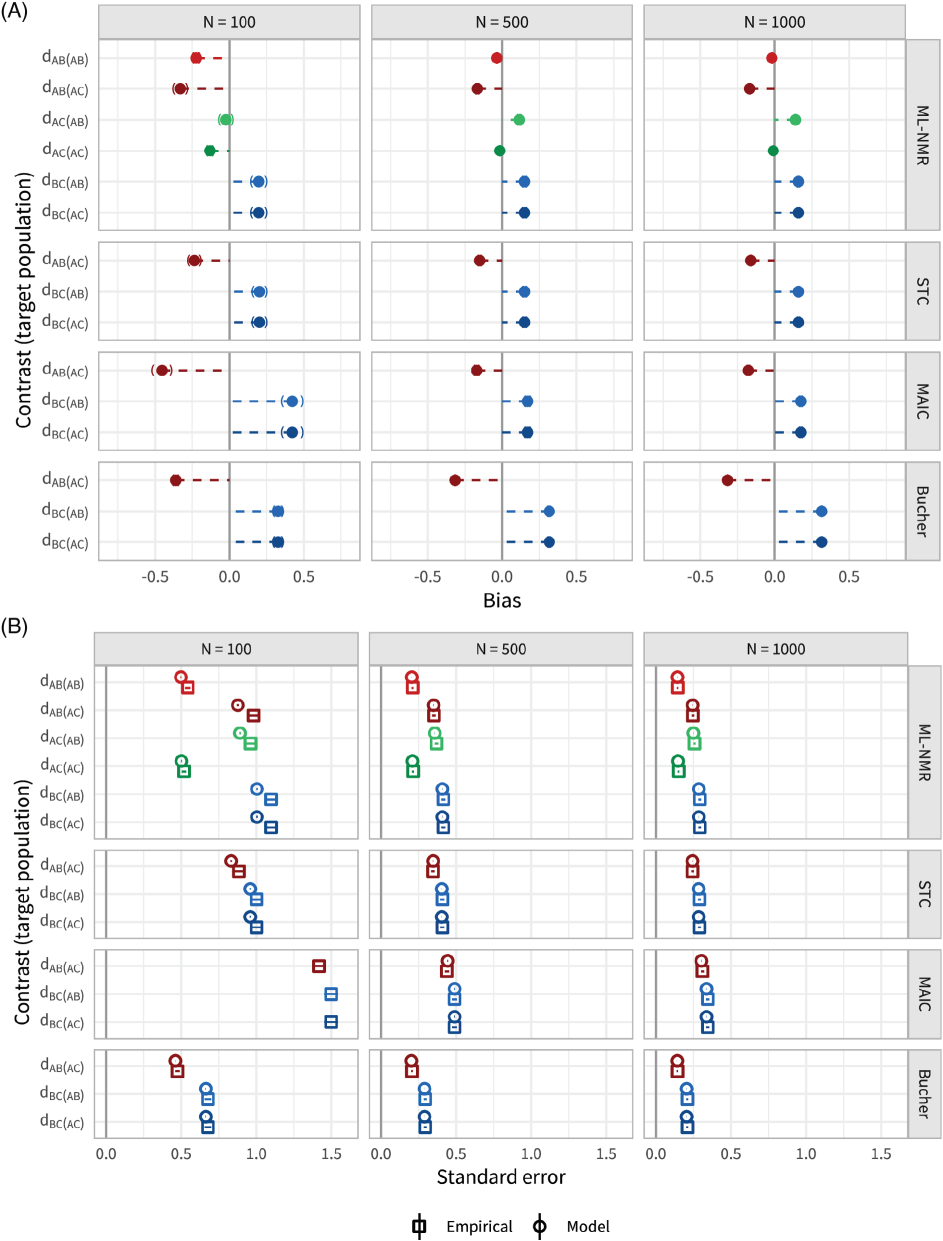
Bias, A, and standard errors, B, for the population‐average contrast estimates for scenario a, along with 95% Monte Carlo confidence intervals. One of the two effect modifiers was not adjusted for. Sample size is varied between 100, 500, and 1000. The points are colored by contrast, with lighter shades for the *AB* population and darker for the *AC* population [Colour figure can be viewed at wileyonlinelibrary.com]

When one of the two effect modifiers is not adjusted for, none of the methods are able to eliminate bias from the estimates (Figure 5A, also Table B2). ML‐NMR and STC show slightly reduced standard errors compared with the models with the full set of effect modifiers (Figure 5B). Again, the empirical standard errors are well estimated by the model standard errors for the larger sample sizes, with a slight underestimation for the smallest sample size. MAIC has markedly reduced standard errors compared with adjustment for both effect modifiers, and the bootstrap model standard errors are now stable for the larger sample sizes. This is because the overlap between studies is necessarily lower as the number of covariates increases. However, MAIC is still unstable for the smallest sample size. Coverage of all three population adjustment methods begins to drop from the nominal level as sample size increases, down to around 92% for sample size 1000 (Figure B2). This is due to the bias remaining in the estimates, as further evidenced by the rightward skew to the zip plots. However, coverage is still well above that of the standard indirect comparison, as bias is reduced. Similar results to Figure 5 are seen across the remaining scenarios when an effect modifier is missing; here onward, we discuss any notable differences in the main text and refer to Appendix B for figures and tabulated results.

### 6.2 Scenario b: Strength of effect modification

**FIGURE 6:**
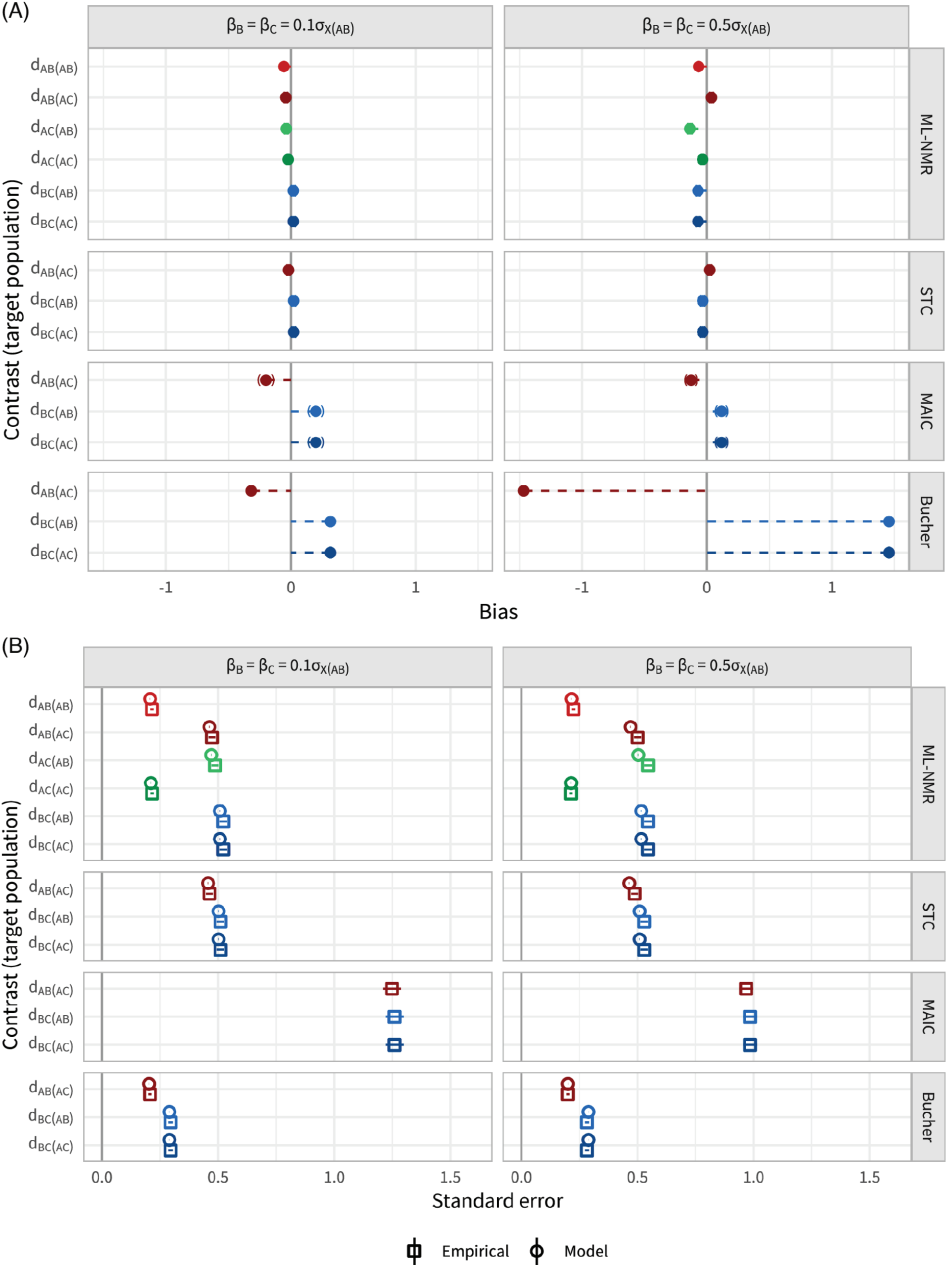
Bias, A, and standard errors, B, for the population‐average contrast estimates for scenario b, along with 95% Monte Carlo confidence intervals. Strength of effect modification is varied from weak (0.1 change in log odds ratio per covariate standard deviation in the *AB* study) to strong (0.5 change in log odds ratio per covariate standard deviation in the *AB* study). Each method (other than Bucher) adjusts for the full set of effect modifiers. The points are colored by contrast, with lighter shades for the *AB* population and darker for the *AC* population [Colour figure can be viewed at wileyonlinelibrary.com]

In scenario b, the strength of effect modification was varied from weak (0.1 change in log odds ratio per covariate standard deviation in the *AB* study) to strong (0.5 change in log odds ratio per covariate standard deviation in the *AB* study), and was the same for both treatments *B* and *C* (so the shared effect modifier assumption holds). Figure 6A shows the bias in the population‐average contrast estimates for each method, adjusting for all effect modifiers, along with 95% Monte Carlo confidence intervals. Figure 6B shows the corresponding empirical and model standard errors. (See also Table B3.) Both ML‐NMR and STC have successfully removed the bias from the standard indirect comparison, and display very similar standard errors. The standard errors are largely unaffected by the strength of the effect modification, although the model standard errors for ML‐NMR and STC slightly underestimate the empirical standard errors when the effect modification is strong. MAIC has also removed most of the bias, though some remains due to lack of overlap between the two studies, and appears to do better when the effect modification is stronger. Empirical standard errors for MAIC are higher than for ML‐NMR and STC, and again the bootstrap model standard errors are extremely unstable. Coverage for all three population adjustment methods is at the nominal level (Figure B3), although there is a small drop for ML‐NMR and STC to 93.0% (91.9, 94.2) and 93.9% (92.9, 94.9) when the effect modification is stronger due to the slight underestimation of the standard error. Coverage for the standard indirect comparison drops to zero when the effect modification is strong, as the incurred bias is so large.

When one of the two effect modifiers is not adjusted for, all population adjustment methods fail to remove the bias (Figure B4a, also Table B4). As expected, the amount of bias remaining is larger when the missing effect modifier is stronger. The standard errors of all population adjustment methods are reduced compared with adjustment for all effect modifiers, and again do not differ by the strength of effect modification (Figure B4b). The coverage for all population adjustment methods drops further when the missing effect modifier is stronger (Figure B5), due to the increased residual bias.

### 6.3 Scenario c: Shared effect modifier assumption

**FIGURE 7:**
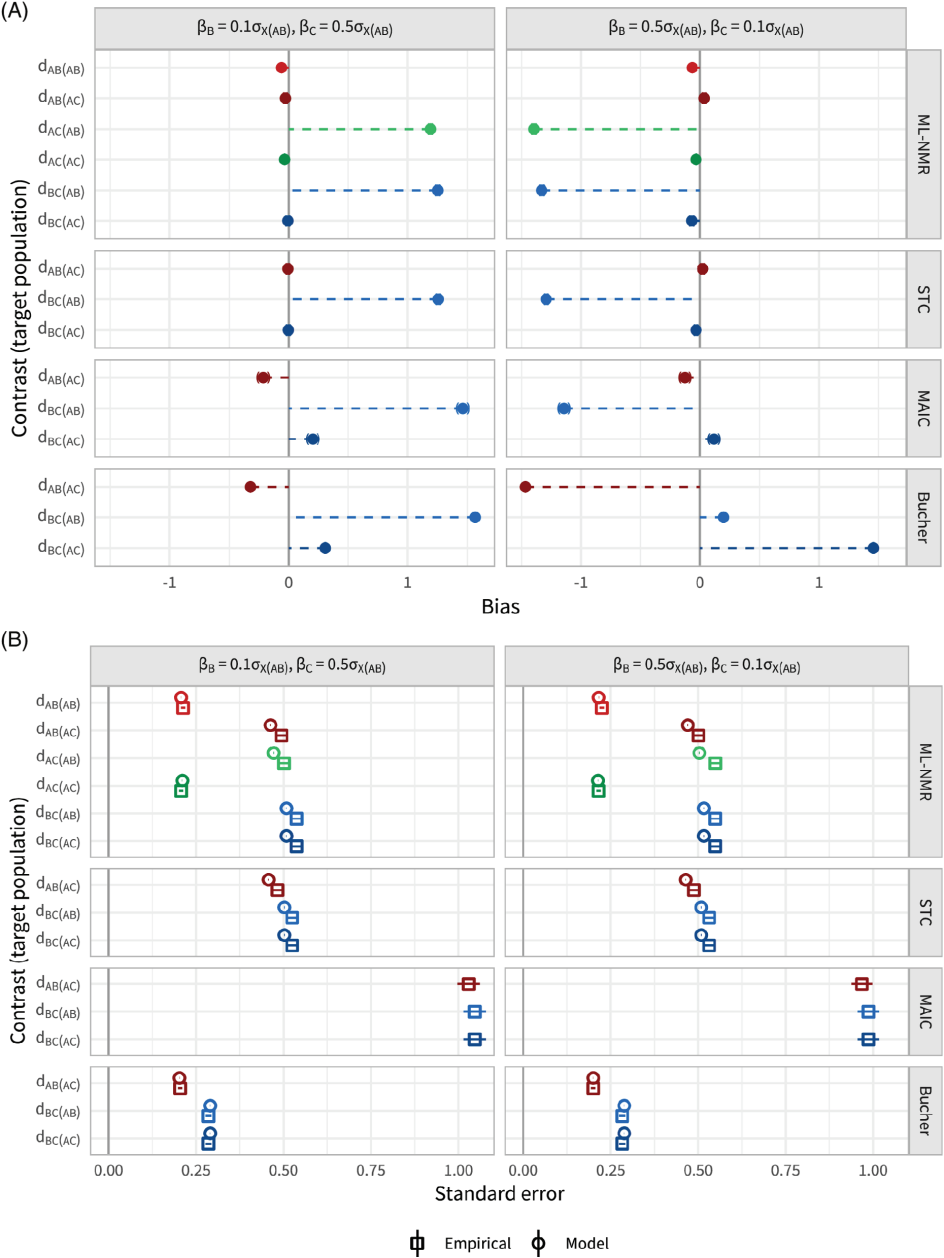
Bias, A, and standard error, B, for the population‐average contrast estimates for scenario c, along with 95% Monte Carlo confidence intervals. Each method (other than Bucher) adjusts for the full set of effect modifiers. The shared effect modifier assumption is broken, so that treatment *B* is subject to weak effect modification, while treatment *C* is subject to strong effect modification and vice versa. The points are colored by contrast, with lighter shades for the *AB* population and darker for the *AC* population [Colour figure can be viewed at wileyonlinelibrary.com]

In scenario c the shared effect modifier assumption is broken, so that treatment *B* is subject to weak effect modification, while treatment *C* is subject to strong effect modification and vice versa. We expect to see that all population adjustment methods are capable of producing unbiased estimates in the *AC* population, but that extrapolation into other populations is biased, and indeed this is what occurs (Figure 7A). Standard errors (Figure 7B) and coverage (Figure B6) are largely unchanged from scenario b (see also Table B5). Again, ML‐NMR and STC show slight underestimation of the empirical standard error when there is strong effect modification, leading to a small drop in coverage down to around 93%.

The increase in bias for a standard indirect comparison when treatment *B* is strongly modified compared with weakly modified (Figure 7A) is due to the focus on the *AC* population. Since a standard indirect comparison does not adjust for population differences, the estimates of *d*_*AB*_ , *d*_*AC*_ , and *d*_*BC*_ are the same regardless of population. However, when the target of inference is a comparison in the *AC* population, the estimate of *d*_*AC*(*AC*)_ is unbiased and bias in the estimate of *d*_*AB*(*AC*)_ (and thus *d*_*BC*(*AC*)_ ) is driven by the strength of effect modification of treatment *B*. If the target of inference was instead a comparison in the *AB* population, this pattern would be reversed (bias now being driven by the strength of effect modification of treatment *C*). A similar pattern, driven by the same mechanism, is observed in scenario b (see Figure 4A).

When one of the two effect modifiers is not adjusted for, further bias is introduced into the estimates, in all target populations (Figure B7a, see also Table B6). This bias is generally larger for estimates involving extrapolation of treatment effects affected by strong unobserved effect modifiers, for example, *d*_*AC*(*AB*)_ and *d*_*BC*(*AB*)_ when *C* is strongly modified by a missing effect modifier. Again, standard error is reduced when fewer effect modifiers are adjusted for (Figure B7b). Coverage for *d*_*BC*(*AC*)_ is close to nominal level for all population adjustment methods when the missing effect modifier for *B* is weak as the incurred bias is small, but drops severely when the missing effect modifier for *B* is strong as the incurred bias is large (Figure B8).

### 6.4 Scenario d: Correlation between covariates

**FIGURE 8:**
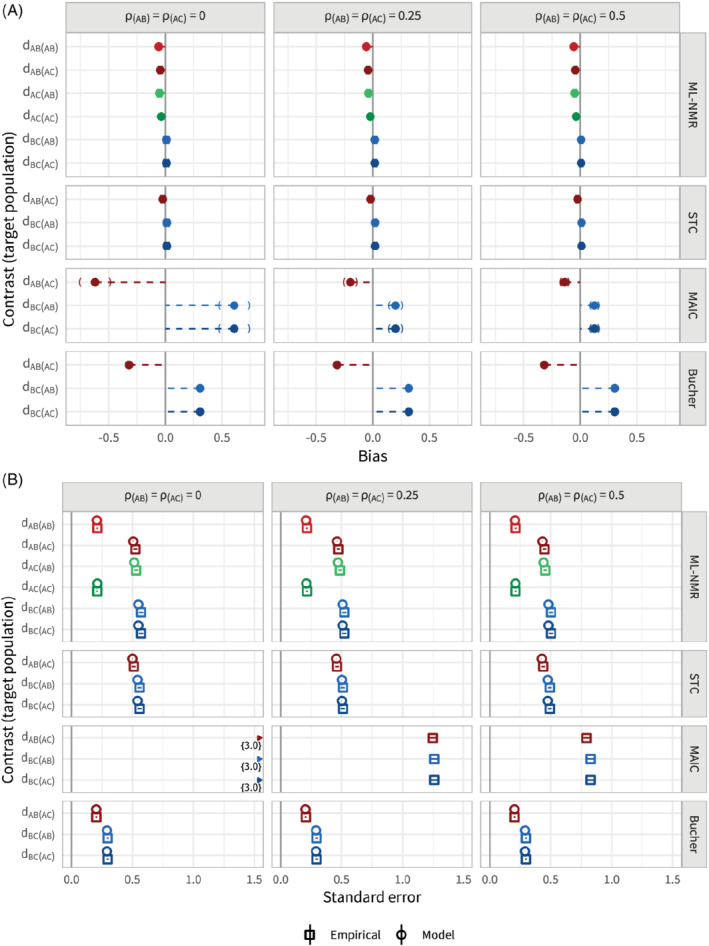
Bias, A, and standard errors, B, for the population‐average contrast estimates for scenario d, along with 95% Monte Carlo confidence intervals. The correlation between covariates is varied between 0, 0.25, and 0.5. Each method (other than Bucher) adjusts for the full set of effect modifiers. The points are colored by contrast, with lighter shades for the *AB* population and darker for the *AC* population [Colour figure can be viewed at wileyonlinelibrary.com]

In scenario d, the correlation between covariates is varied between 0, 0.25, and 0.5, and the correlation is the same in both study populations. Figure 8A shows that ML‐NMR and STC are unaffected by the correlation between covariates, achieving bias removal regardless of the correlation. Standard errors are similarly unaffected (Figure 8B), and coverage is at the nominal level (Figure B10). Table B7 shows the results in tabular format. For MAIC, both bias and standard error are reduced as the correlation between covariates increases. This is because, as the correlation increases, the effective number of covariates decreases and the overlap between study populations increases (Figure B9). The simulation parameter κ is only a proxy for overlap and does not account for correlation between covariates, so the true overlap changes with the correlation despite holding κ constant at 0.5. Since MAIC cannot eliminate the bias, coverage drops as the standard error decreases (as the correlation increases), down to 93.4% (92.3, 94.6) when the correlation is 0.5.

When one of the two effect modifiers is not adjusted for, all population adjustment methods produce biased estimates (Figure B11a, see also Table B8). However, as we expect, the amount of bias reduces as the correlation between the observed and missing effect modifiers increases. If we were to continue simulations with correlations tending closer to 1, the bias due to a missing effect modifier would disappear entirely. Again, standard error is reduced when fewer effect modifiers are adjusted for (Figure B11b). Coverage for all population adjustment methods was slightly below the nominal level (around 93%) due to the remaining bias (Figure B12).

### 6.5 Scenarios e and f: Between‐study overlap and covariate‐outcome relationship

In scenarios e and f, the between‐study overlap and covariate‐outcome relationship are varied jointly. The between‐study overlap is varied between 0 (no overlap, all of *AC* population outside of the *AB* population), 0.5 (approximately 50% of *AC* outside of *AB* ), and 1 (full overlap, all of *AC* within *AB* ), and the covariate‐outcome relationship is either linear or nonlinear (Equation (10)).

**FIGURE 9:**
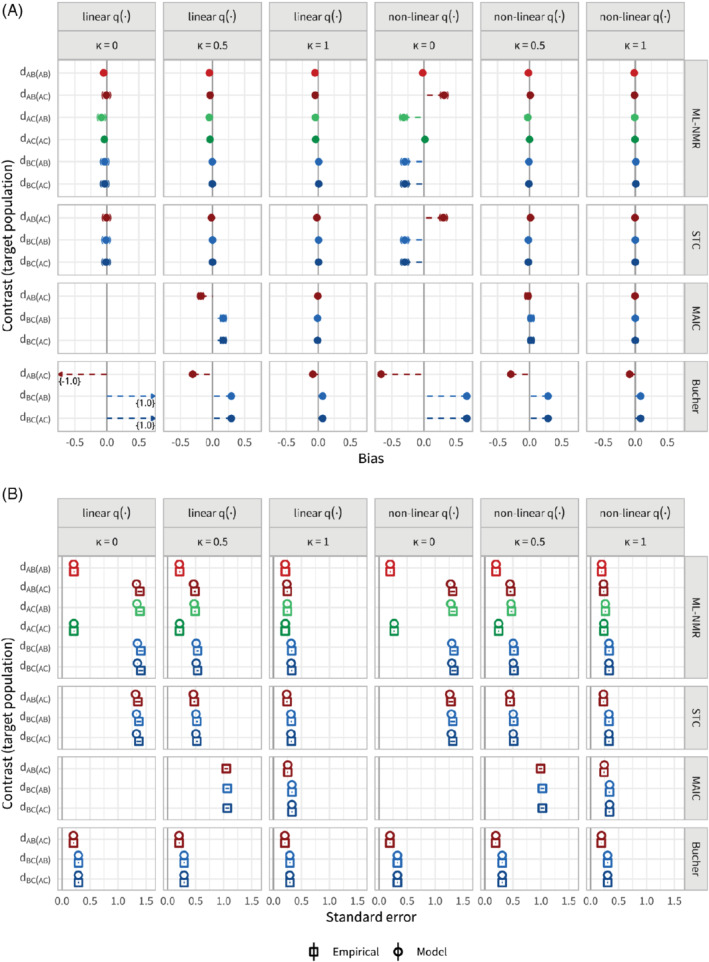
Bias, A, and standard errors, B, the population‐average contrast estimates for scenarios e and f, along with 95% Monte Carlo confidence intervals. Each method (other than Bucher) adjusts for the full set of effect modifiers. The between‐study overlap and covariate‐outcome relationship are varied jointly. The points are colored by contrast, with lighter shades for the *AB* population and darker for the *AC* population [Colour figure can be viewed at wileyonlinelibrary.com]

When the covariate‐outcome relationship is linear, both ML‐NMR and STC produce unbiased estimates (Figure 9A, Table B9). MAIC is unable to produce any estimates when there is no overlap between study populations, and remains biased when the overlap is 0.5. Only when the study populations overlap completely does MAIC produce unbiased estimates.

When the covariate‐outcome relationship is nonlinear, ML‐NMR and STC (set to fit a linear relationship) are both biased when there is no overlap between the study populations. There is no discernible bias when the overlap is 0.5 or 1, as Equation (10) behaves linearly within the range of the *AB* population. However, prediction into another target population with a greater difference in covariate values from the *AB* and *AC* populations would still result in bias. Again, MAIC cannot produce estimates when there is no overlap between study populations.

Standard errors for all population adjustment methods decrease as the level of overlap increases (Figure 9B). Empirical standard errors are well estimated by model standard errors for ML‐NMR and STC; however, MAIC only produced stable bootstrap model standard errors when the overlap was 1.

For the population adjustment methods, nominal coverage is achieved when the covariate‐outcome relationship is linear (Figure B13). With a nonlinear relationship, coverage drops slightly to 93.0% (91.9, 94.2) for ML‐NMR and to 93.4% (92.4, 94.5) when there is no overlap, but is unaffected at 0.5 and full overlap as there is no discernible bias. MAIC achieves nominal coverage for 0.5 and full overlap.

When one of the two effect modifiers is not adjusted for, all population adjustment methods produce biased estimates (Figure B14a, Table B10). As expected, the bias due to the missing effect modifier reduces as the overlap between studies increases, since the difference in the missing effect modifier between the study populations becomes smaller. Due to this, coverage shows the opposite relationship with overlap, dropping as the overlap decreases (Figure B15). Again, standard error is reduced when fewer effect modifiers are adjusted for (Figure B14b).

### 6.6 Scenarios g, h, and i: Covariate distributions and correlation structures

In scenarios g, h, and i, the covariate distributions and correlation structures in each study population are varied jointly. The covariate distributions are set to either Normal or Gamma in each of the study populations, and the correlation between covariates in the *AC* population was varied between 0, 0.25, and 0.5. The correlation between covariates in the *AB* population was held constant at 0.25.

We are particularly interested in the performance of ML‐NMR in this scenario, since ML‐NMR makes the assumption that distributional form and correlation structure are the same in the AgD studies as in the IPD. Scenarios g, h, and i break this assumption. Despite this, ML‐NMR is not seen to incur any bias when the distributional form and/or correlation structure are different in each population (Figure B16). The empirical standard errors, and their estimation by the model standard errors, are also unaffected (Figure B17). The bias and standard error of MAIC are slightly smaller when the covariates in the *AB* population are Gamma distributed compared to Normally distributed; however, this is due to the skew of the Gamma distribution slightly increasing the true overlap between the study populations (compare row 1 with 3, and 2 with 4, in Figure B18). Nominal coverage is achieved by all population adjustment methods, regardless of the covariate distributions or correlation structure (Figure B19). See Table B11 for a table of these results.

Once again, when one of the two effect modifiers is not adjusted for, all population adjustment methods produce biased estimates (Figure B20) but standard error is reduced (Figure B21). Coverage is slightly reduced for all population adjustment methods, to around 94%, as a result of the bias (Figure B19). The results are tabulated in Table B12.

## 7 DISCUSSION

In this simulation study, we have investigated the performance of ML‐NMR in comparison with current population adjustment methods (MAIC and STC) in a wide range of scenarios, including varying sample sizes, strength of effect modification, overlap between studies, and joint covariate distributions. Scenarios were chosen to be realistic, reflecting the issues raised by the applied example and the findings of two recent reviews of applications.^22^, ^25^ As well as validating the performance of the different methods when assumptions were met, we were particularly interested in how the methods fared when assumptions were broken in realistic ways. We investigated conditional constancy of relative effects (ie, no missing effect modifiers), the shared effect modifier assumption, validity of extrapolation beyond the IPD study population, and (for ML‐NMR) correctly specifying the form of the marginal distributions and correlations between covariates in the AgD study.

ML‐NMR and STC performed very similarly throughout the simulation study. This is to be expected, as the two methods are both regression adjustment methods. Both methods were seen to perform well when the requisite assumptions were met, largely eliminating the bias incurred by a standard indirect comparison and estimating standard errors well. ML‐NMR offers additional advantages over STC, including the ability to synthesize a larger network of treatments, and to produce estimates in any target population. ML‐NMR makes explicit assumptions about the covariate distributions and correlation structure in the aggregate population in order to derive the aggregate likelihood through integration, typically that they are the same as in the IPD population. However, the performance of ML‐NMR was not found to be sensitive to this assumption (see scenarios g, h, and i, Section 6.6). MAIC and STC typically ignore covariate correlations (Section 4.5), but the performance of these methods was also not seen to be sensitive to the implicit assumptions that this invokes.

Serious questions must be raised about the use of MAIC for population adjustment. MAIC performed poorly in all simulation scenarios, and in some cases even increased the bias compared with a standard indirect comparison. Bootstrap error estimation was also extremely unstable, except for the largest sample sizes. The issues with MAIC stem from the fact that it is a reweighting method, and therefore cannot extrapolate. As such, bias can only be completely removed when the population of the AgD study is entirely contained within the population of the IPD study (see scenario e, Section 6.5). However, when the two study populations overlap entirely there is unlikely to be much bias in a standard indirect comparison as the imbalance in effect modifiers is necessarily small, and thus population adjustment methods may not be needed. Furthermore, this means that MAIC is only valid from at most one company's perspective of the indirect comparison—that of the company whose study (for which they have IPD) has a broader distribution of covariates. MAIC analysis from the other company's perspective (who has IPD on the other study, with a more restricted covariate distribution) will result in estimates that are biased, possibly by more than a standard indirect comparison (as well as being produced for a different target population). If the two study populations do not overlap at all, then no MAIC analyses are possible. The simulation study therefore raises serious questions regarding whether MAIC is fit for purpose. Previous simulation studies investigating MAIC have not observed this issue regarding overlap.^26^, ^27^, ^42^, ^43^, ^44^, ^45^ For some studies,^26^, ^27^ this is because they were not designed to vary the overlap of continuous covariates between studies, instead basing simulations on scenarios with good overlap where, as we found, MAIC works well. For others,^44^, ^45^ this is because they were focused on binary covariates, where issues only arise when covariate proportions are close to zero or one in the IPD study. (The remaining studies^42^, ^43^ were available only as conference abstracts, and full details of the simulation scenarios were not available.) It is particularly worrying that, in practical applications of MAIC, study sample sizes are typically small and there is often a lack of overlap between studies (as evidenced by large reductions in effective sample size)^25^—situations where we have shown MAIC to perform poorly. Given the severe issues with MAIC surrounding covariate overlap, we find it unlikely that there are realistic scenarios where MAIC may consistently outperform STC and ML‐NMR.

In this simulation study, we focused on continuous covariates only. However, the underlying assumptions made by each of the methods are unchanged whether covariates are discrete or continuous, and so we expect the conclusions of this simulation study to apply regardless of the type of covariates. Continuous covariates were chosen for this simulation study since issues with between‐study overlap can arise in much less extreme situations with continuous covariates than with discrete covariates. In the binary covariate case, issues only appear as the covariate proportions in the IPD study approach 0 or 1, at which point no methods will work (there is no information to estimate the interaction term for ML‐NMR or STC, and no individuals to reweight for MAIC). When covariate proportions are not close to 0 or 1 there can be no overlap issues—estimates may be imprecise if groups are small, but they should not be biased. We would expect the performance of all methods with binary covariates to be similar to that seen in the full overlap scenario in this simulation study, except when proportions are close to 0 or 1 (when no methods will work). This also aligns with the results of previous simulation studies based on binary covariates,^44^, ^45^ which have observed no bias with nonextreme covariate proportions. The continuous covariate scenario is of greater practical concern, as there is greater potential for methods to break down. Practical applications often involve a mix of discrete and continuous covariates, as demonstrated in the plaque psoriasis example (Section 3). As we have seen with continuous covariates bias can occur for MAIC even when the overlap is moderate, and ML‐NMR and STC must be concerned with the validity of extrapolation (Section 6.5, scenarios e and f).

We have not considered including correlations between covariates in the MAIC matching in this simulation study, as these are typically unavailable and we are unaware of any practical applications where this has been done (see Section 4.5). Nevertheless, it is possible to also match covariate correlations to the aggregate study in MAIC if these are known or perhaps imputed from the IPD study. However, we did not find any notable impact of correlations on the performance of MAIC (or indeed ML‐NMR or STC) in the scenarios considered, except through the impact on effective overlap (see scenarios d, g, h, and i, Sections 6.4 and 6.6).

For this simulation study the estimands of interest are adjusted population‐average treatment effects in a given study population, as would be estimated by a “gold‐standard” IPD network meta‐regression with a correctly specified regression model. Due to the noncollapsibility of the log odds ratio, these estimands differ from the unadjusted population‐average treatment effects (ie, the unadjusted crude log odds ratios in each study population). MAIC, as it is typically implemented (see Section 2.3), targets this latter estimand since unadjusted log odds ratios (after weighting) are combined. This mismatch of estimands has the potential to introduce additional bias into our evaluation of MAIC; however, this does not seem to be the case in the scenarios considered here, as MAIC is seen to estimate an unbiased treatment effect when there is full overlap between study populations (Section 6.5). The typical implementation of STC which we have used^22^ is flawed for noncollapsible effect measures since it combines an adjusted effect estimate ( *d*_*AB*(*AC*)_ from the STC model) with an unadjusted effect estimate ( *d*_*AC*(*AC*)_ from the *AC* trial). We assessed this version of STC, since it seems to be the most prevalent in the literature. Moreover, a corrected version of STC combining two adjusted estimates requires that the aggregate *AC* study reports a *d*_*AC*(*AC*)_ treatment effect estimate that has been adjusted in the same manner as the STC regression model, which seems unlikely to be available in practice. Our simulations did not show any noticeable bias for STC when the requisite assumptions were met, despite the flawed combination of estimands; however, other simulation studies have demonstrated bias for STC due to this issue.^45^ Both MAIC and STC (and indeed standard indirect comparisons and NMA) may incur bias if incompatible estimates are combined. ML‐NMR correctly combines adjusted and unadjusted effect measures at each level of the model through the integration in Equation (8d). Furthermore, ML‐NMR can produce estimates of any population‐average quantity of interest, including unadjusted treatment effects, through integration over the corresponding covariate distribution.^16^

Although MAIC and STC are designed with a two‐study indirect comparison scenario in mind, it is commonplace for larger networks of treatments and studies to be available in practice (as demonstrated in the plaque psoriasis example, Section 3). In the review of applied literature, 73% of applications involved multiple IPD or AgD studies and/or additional treatments,^22^ and 56% of technology appraisals had larger networks present.^25^ ML‐NMR extends naturally to larger networks of studies and treatments, as we describe in Section 2.4. While this simulation study focused on the two‐study scenario for comparison with MAIC, STC, and standard indirect comparison, we expect the conclusions regarding ML‐NMR to extend to larger networks since the underlying assumptions remain the same. Further research could explore the properties of ML‐NMR in larger networks in more detail.

Although ML‐NMR offers the possibility to analyze larger networks of studies and treatments, with larger networks of evidence there is increased potential for heterogeneity and inconsistency—and also the possibility to check for such issues. When ML‐NMR is used in larger networks, Phillippo et al^16^ suggest performing standard checks for heterogeneity and inconsistency.^46^ The presence of residual heterogeneity or inconsistency may indicate a failure of assumptions, such as unobserved effect modifiers or an invalid shared effect modifier assumption.

The ML‐NMR model described in Section 2.4 assumes that aggregate studies provide outcome data on each arm (eg, event counts or mean outcomes). However, this can be extended to incorporate contrast‐based aggregate data (eg, log odds ratios or mean differences) by using a Normal likelihood for studies providing evidence as contrasts (multivariate Normal for multi‐arm studies), with linear predictor given by Equation (9).^15^ This is analogous to the usual approach to handling contrast‐based data in NMA.^6^, ^47^, ^48^

MAIC and STC require the shared effect modifier assumption in order to produce estimates outside of the *AC* study population. We also made this assumption for ML‐NMR to identify the model in the two‐study scenario. This assumption may be relaxed for ML‐NMR in larger networks if sufficient data are available, but this is data‐intensive.^15^ If additional information is available from aggregate studies such as reported regression coefficients or subgroup analyses, this can be used to aid estimation of a ML‐NMR model. For example, if regression coefficients and their covariance matrix are reported then these may be used as an informative prior distribution, and factorial subgroup analyses may be incorporated through a simple extension of the numerical integration approach.^15^ However, further research is necessary to consider scenarios where regression coefficients are only univariate or reported without correlations, or where multiple univariate subgroup analyses are reported. Moreover, reporting bias may be introduced if “significant” regression coefficients or subgroup analyses are selectively reported, and care should be taken to only consider evidence from prespecified analyses.

Practical applications of population adjustment include a large majority of “unanchored” comparisons, involving single arm studies or where there is no common comparator.^22^, ^25^ In this case, the expected outcomes *Y*_*B*(*P* )_ and *Y*_*C*(*P* )_ on each treatment are compared directly

(11) $$d_{BC(P)}=g(Y_{C(P)})-g(Y_{B(P)}),$$

on a suitable transformed scale via the link function *g*(·). Unanchored indirect comparisons rely on the much stronger assumption of *conditional constancy of absolute effects*.^22^, ^23^ This requires that all prognostic variables as well as all effect modifiers are suitably adjusted for, in order to reliably predict absolute outcomes across populations. Quantifying the magnitude of residual bias due to unobserved effect modifiers or prognostic factors is crucial; some approaches are suggested by Phillippo et al,^22^ and this is an area for further research. A common comparator with which to make an anchored comparison was available in 64% of the applications in the published literature;^25^ however, only 11% of technology appraisals employing population adjustment methods used anchored comparisons. While we have focused on simulation scenarios involving “anchored” indirect comparisons of randomized controlled trials with a common comparator treatment, we expect the results and conclusions to apply broadly to unanchored comparisons also. The key difference is that bias will be present if any prognostic variables, not just effect modifiers, are also missing from the analysis. As a result, the required number of adjustment variables will be higher, and this implies that between‐study overlap will be further reduced and that larger sample sizes may be necessary to achieve good bias correction. Ongoing research looks to extend ML‐NMR to incorporate evidence from single‐arm or observational studies, for example, by extending approaches previously used for NMA,^49^, ^50^, ^51^, ^52^ and to investigate methods of verifying the strong assumptions required.

Standard model fit and model comparison techniques may be employed for the ML‐NMR model described in Section 2.4, such as the Deviance Information Criterion.^53^ With more general individual‐level likelihoods where the aggregate likelihood has no closed form (eg, for time‐to‐event data), alternative model comparison techniques are required,^15^ and this is an area for further research. However, we recommend that (at least for technology assessment purposes) applicable guidance on covariate selection is followed; that is, effect‐modifying covariates should be prespecified.^22^

As regression methods, ML‐NMR and STC are able to extrapolate beyond the range of the IPD, producing estimates even when there is no overlap between study populations. However, when extrapolation occurs, estimates will only be unbiased if such extrapolation is valid. For example, in scenarios e and f (Section 6.5), ML‐NMR and STC produced biased estimates when there was no overlap between populations and the true covariate‐outcome relationship was nonlinear outside of the range of the IPD, but only a linear relationship was accounted for (Figure 3).

Notably, all population adjustment methods are susceptible to bias (and as a result, undercoverage) when an effect modifier is missing from the adjustment, and thus the conditional constancy of relative effects assumption is invalid. This highlights the necessity of careful and considered selection of potential effect modifiers prior to analysis.^22^, ^23^ When all effect modifiers have been identified and included in the adjustment model, ML‐NMR and STC are both robust techniques for obtaining population‐adjusted indirect comparisons. ML‐NMR offers additional advantages over MAIC and STC, including extending naturally to incorporate larger networks of evidence and producing estimates in any target population of interest,^16^ making this an attractive choice for population adjustment in a wide variety of scenarios.

## CONFLICT OF INTEREST

David M. Phillippo reports personal fees from UCB outside of the submitted work.

## Supporting information

Data S1 Appendix B

Appendix

## Data Availability

R code used to perform and analyze the simulation study is available in the online supporting materials. Data and R code for the plaque psoriasis example are openly available from https://doi.org/10.1111/rssa.12579 accompanying the paper by Phillippo et al, reference.^16^

## Appendix A 1

### A1 Defining between‐study overlap

There is no standard definition of the “overlap” between study populations. In this article, we define the overlap between the study populations as the proportion of the *AC* joint density $\phi_{\left(AC\right)}$ contained within the 95% highest density region (HDR) of the *AB* joint density $\phi_{\left(AB\right)}$. Thus the overlap is given by the integral

(A1)
$$\int_{X}𝕀\left(x\in R_{(AB),0.05}\right)\phi_{\left(AC\right)}(x)dx,$$

where the (1−α)% HDR is the region $R_{(AB),\alpha }=\{x:\phi_{\left(AB\right)}(x)\geq c_{(AB),\alpha }\}$, with $c_{(AB),\alpha }$ the largest constant satisfying $\mathbb{P}(x\in R_{(AB),\alpha })\geq 1-\alpha$. Clearly the exact relationship between the parameters of the joint distribution in each study and the true overlap is highly complex, and so instead we vary the proxy parameter κ in our simulation study. The “true” overlap can be calculated using numerical integration to evaluate the integral (A1).

## References

[1] Bucher HC , Guyatt GH , Griffith LE , Walter SD . The results of direct and indirect treatment comparisons in meta‐analysis of randomized controlled trials. J Clin Epidemiol. 1997;50(6):683‐691. 10.1016/s0895-4356(97)00049-8. 9250266

[2] Higgins JPT , Whitehead A . Borrowing strength from external trials in a meta‐analysis. Stat Med. 1996;15(24):2733‐2749. 10.1002/(sici)1097-0258(19961230)15:24<2733::aid-sim562>3.0.co;2-0. 8981683

[3] Ades AE . A chain of evidence with mixed comparisons: models for multi‐parameter synthesis and consistency of evidence. Stat Med. 2003;22(19):2995‐3016. 10.1002/sim.1566. 12973783

[4] Lu GB , Ades AE . Combination of direct and indirect evidence in mixed treatment comparisons. Stat Med. 2004;23(20):3105‐3124. 10.1002/sim.1875. 15449338

[5] Lu GB , Ades AE . Assessing evidence inconsistency in mixed treatment comparisons. J Am Stat Assoc. 2006;101(474):447‐459. 10.1198/016214505000001302.

[6] Dias S , Welton NJ , Sutton AJ , Ades AE . NICE DSU Technical Support Document 2: A Generalised Linear Modelling Framework for Pairwise and Network Meta‐Analysis of Randomised Controlled Trials. London, UK: National Institute for Health and Care Excellence; 2011. http://www.nicedsu.org.uk/. 27466657

[7] Dias S , Sutton AJ , Welton NJ , Ades AE . NICE DSU Technical Support Document 3: Heterogeneity: Subgroups, Meta‐Regression, Bias and Bias‐Adjustment. London, UK: National Institute for Health and Care Excellence; 2011. http://www.nicedsu.org.uk/ 27905717

[8] Berlin JA , Santanna J , Schmid CH , Szczech LA , Feldman HI . Individual patient‐ versus group‐level data meta‐regressions for the investigation of treatment effect modifiers: ecological bias rears its ugly head. Stat Med. 2002;21(3):371‐387. 10.1002/sim.1023. 11813224

[9] Lambert PC , Sutton AJ , Abrams KR , Jones DR . A comparison of summary patient‐level covariates in meta‐regression with individual patient data meta‐analysis. J Clin Epidemiol. 2002;55(1):86‐94. 10.1016/S0895-4356(01)00414-0. 11781126

[10] Tudur SC , Williamson PR , Marson AG . Investigating heterogeneity in an individual patient data meta‐analysis of time to event outcomes. Stat Med. 2005;24(9):1307‐1319. 10.1002/sim.2050. 15685717

[11] Riley RD , Lambert PC , Abo‐Zaid G . Meta‐analysis of individual participant data: rationale, conduct, and reporting. Br Med J. 2010;340: c221. 10.1136/bmj.c221. 20139215

[12] Signorovitch JE , Wu EQ , Yu AP , et al. Comparative effectiveness without head‐to‐head trials a method for matching‐adjusted indirect comparisons applied to psoriasis treatment with adalimumab or etanercept. Pharmaceconomics. 2010;28(10):935‐945. 10.2165/11538370-000000000-00000. 20831302

[13] Ishak KJ , Proskorovsky I , Benedict A . Simulation and matching‐based approaches for indirect comparison of treatments. Pharmacoeconomics. 2015;33(6):537‐549. 10.1007/s40273-015-0271-1. 25795232

[14] Caro JJ , Ishak KJ . No head‐to‐head trial? Simulate the missing arms. Pharmaceconomics. 2010;28(10):957‐967. 10.2165/11537420-000000000-00000 20831304

[15] Phillippo DM . Calibration of Treatment Effects in Network Meta‐Analysis Using Individual Patient Data [PhD thesis]. University of Bristol; 2019. https://research‐information.bris.ac.uk/.

[16] Phillippo DM , Dias S , Ades AE , et al. Multilevel network meta‐regression for population‐adjusted treatment comparisons. J R Stat Soc A Stat Soc. 2020;183(3):1189‐1210. 10.1111/rssa.12579. PMC7362893 32684669

[17] Donegan S , Williamson P , D'Alessandro U , Garner P , Tudur SC . Combining individual patient data and aggregate data in mixed treatment comparison meta‐analysis: individual patient data may be beneficial if only for a subset of trials. Stat Med. 2013;32(6):914‐930. 10.1002/sim.5584. 22987606

[18] Saramago P , Sutton AJ , Cooper NJ , Manca A . Mixed treatment comparisons using aggregate and individual participant level data. Stat Med. 2012;31(28):3516‐3536. 10.1002/sim.5442. 22764016

[19] Sutton AJ , Kendrick D , Coupland CAC . Meta‐analysis of individual‐ and aggregate‐level data. Stat Med. 2008;27(5):651‐669. 10.1002/sim.2916. 17514698

[20] Riley RD , Lambert PC , Staessen JA , et al. Meta‐analysis of continuous outcomes combining individual patient data and aggregate data. Stat Med. 2008;27(11):1870‐1893. 10.1002/sim.3165. 18069721

[21] Riley RD , Steyerberg EW . Meta‐analysis of a binary outcome using individual participant data and aggregate data. Res Synth Methods. 2010;1(1):2‐19. 10.1002/jrsm.4. 26056090

[22] Phillippo DM , Ades AE , Dias S , Palmer S , Abrams KR , Welton NJ . NICE DSU Technical Support Document 18: Methods for Population‐Adjusted Indirect Comparisons in Submission to NICE. London, UK: National Institute for Health and Care Excellence; 2016. http://www.nicedsu.org.uk/.

[23] Phillippo DM , Ades AE , Dias S , Palmer S , Abrams KR , Welton NJ . Methods for population‐adjusted indirect comparisons in health technology appraisal. Med Decis Mak. 2018;38(2):200‐211. 10.1177/0272989x17725740. PMC5774635 28823204

[24] Veroniki AA , Straus SE , Soobiah C , Elliott MJ , Tricco AC . A scoping review of indirect comparison methods and applications using individual patient data. BMC Med Res Methodol. 2016;16(1):1‐14. 10.1186/s12874-016-0146-y. 27116943 PMC4847203

[25] Phillippo DM , Dias S , Elsada A , Ades AE , Welton NJ . Population adjustment methods for indirect comparisons: a review of national institute for health and care excellence technology appraisals. Int J Technol Assess Health Care. 2019;35(03):221‐228. 10.1017/S0266462319000333. 31190671 PMC6650293

[26] Belger M , Brnabic A , Kadziola Z , Petto H , Faries D . Inclusion of multiple studies in matching adjusted indirect comparisons (MAIC). Paper presented at: Proceedings of the ISPOR 20th Annual International Meeting; 2015; Philadelphia, PA.

[27] Belger M , Brnabic A , Kadziola Z , Petto H , Faries D . Alternative weighting approaches for matching adjusted indirect comparisons (MAIC). Paper presented at: Proceedings of the ISPOR 20th Annual International Meeting; 2015; Philadelphia, PA.

[28] Hainmueller J . Entropy balancing for causal effects: a multivariate reweighting method to produce balanced samples in observational studies. Polit Anal. 2012;20(1):25‐46. 10.1093/pan/mpr025.

[29] Phillippo DM , Dias S , Ades AE , Welton NJ . Equivalence of entropy balancing and the method of moments for matching‐adjusted indirect comparison. Res Synth Methods. 2020;11(4):568‐572. 10.1002/jrsm.1416. PMC7384548 32395870

[30] Griffiths CEM , Reich K , Lebwohl M , et al. Comparison of ixekizumab with etanercept or placebo in moderate‐to‐severe psoriasis (UNCOVER‐2 and UNCOVER‐3): results from two phase 3 randomised trials. Lancet. 2015;386(9993):541‐551. 10.1016/s0140-6736(15)60125-8. 26072109

[31] Gordon KB , Blauvelt A , Papp KA , et al. Phase 3 Trials of ixekizumab in moderate‐to‐severe plaque psoriasis. N Engl J Med. 2016;375(4):345‐356. 10.1056/nejmoa1512711. 27299809

[32] Langley RG , Elewski BE , Lebwohl M , et al. Secukinumab in plaque psoriasis — results of two phase 3 Trials. N Engl J Med. 2014;371(4):326‐338. 10.1056/nejmoa1314258. 25007392

[33] Strober B , Brnabic A , Schacht A , et al. Indirect comparison of ixekizumab and secukinumab using matched‐adjusted indirect comparisons. Paper presented at: Proceedings of the 25th Congress of the European Academy of Dermatology and Venereology; Vienna, Austria, 2016.

[34] Carpenter B , Gelman A , Hoffman MD , et al. Stan: a probabilistic programming language. J Stat Softw. 2017;76(1):1‐32. 10.18637/jss.v076.i01. PMC9788645 36568334

[35] Gelman A , Carlin JB , Stern HS , Dunson DB , Vehtari A , Rubin DB . Bayesian Data Analysis. Texts in Statistical Science. 3rd ed. Boca Raton, FL: Chapman & Hall/CRC Press; 2013.

[36] Gail MH , Wieand S , Piantadosi S . Biased estimates of treatment effect in randomized experiments with nonlinear regressions and omitted covariates. Biometrika. 1984;71(3):431‐444. 10.1093/biomet/71.3.431.

[37] Greenland S , Robins JM , Pearl J . Confounding and collapsibility in causal inference. Stat Sci. 1999;14(1):29‐46.

[38] Morris TP , White IR , Crowther MJ . Using simulation studies to evaluate statistical methods. Stat Med. 2019;38(11):2074‐2102. 10.1002/sim.8086. 30652356 PMC6492164

[39] R Core Team . R: A Language and Environment for Statistical Computing. Vienna, Austria: R Foundation for Statistical Computing; 2017.

[40] Hofert M , Mächler M . Parallel and other simulations in R made easy: an end‐to‐end study. J Stat Softw. 2016;69(4):1‐44. 10.18637/jss.v069.i04.

[41] Vittinghoff E , McCulloch CE . Relaxing the rule of ten events per variable in logistic and Cox regression. Am J Epidemiol. 2007;165(6):710‐718. 10.1093/aje/kwk052. 17182981

[42] Signorovitch JE , Ayyagari R , Cheng D , Wu EQ . Matching‐adjusted indirect comparisons: a simulation study of statistical performance. Paper presented at: Proceedings of the ISPOR 18th Annual International Meeting; 2013; New Orleans, LA.

[43] Hatswell AJ , Freemantle N , Baio G . Does matching adjusted indirect comparison (MAIC) work? results from a simulation study. Paper presented at: Proceedings of the ISPOR European Meeting; 2018; Barcelona, Spain.

[44] Leahy J , Walsh C . Assessing the impact of a matching‐adjusted indirect comparison in a Bayesian network meta‐analysis. Res Synth Methods. 2019;10(4):546‐568. 10.1002/jrsm.1372. 31368653

[45] Remiro‐Azócar A , Heath A , Baio G . Methods for population adjustment with limited access to individual patient data: a simulation study; 2020. arXiv: 2004.14800v1 [stat.AP]. 10.1002/jrsm.1511 34196111

[46] Dias S , Welton NJ , Sutton AJ , Caldwell DM , Lu G , Ades AE . NICE DSU Technical Support Document 4: Inconsistency in Networks of Evidence Based on Randomised Controlled Trials. London, UK: National Institute for Health and Care Excellence; 2011. http://www.nicedsu.org.uk/. 27466656

[47] Salanti G , Higgins JPT , Ades AE , Ioannidis JPA . Evaluation of networks of randomized trials. Stat Methods Med Res. 2007;17(3):279‐301. 10.1177/0962280207080643. 17925316

[48] Woods BS , Hawkins N , Scott DA . Network meta‐analysis on the log‐hazard scale, combining count and hazard ratio statistics accounting for multi‐arm trials: a tutorial. BMC Med Res Methodol. 2010;10(1). 10.1186/1471-2288-10-54. PMC2906500 20537177

[49] Li Z , Begg CB . Random effects models for combining results from controlled and uncontrolled studies in a meta‐analysis. J Am Stat Assoc. 1994;89(428):1523‐1527. 10.1080/01621459.1994.10476892.

[50] Thom HZ , Capkun G , Cerulli A , Nixon RM , Howard LS . Network meta‐analysis combining individual patient and aggregate data from a mixture of study designs with an application to pulmonary arterial hypertension. BMC Med Res Methodol. 2015;15(1):34. 10.1186/s12874-015-0007-0. 25887646 PMC4403724

[51] Schmitz S , Adams R , Walsh C . Incorporating data from various trial designs into a mixed treatment comparison model. Stat Med. 2013;32(17):2935‐2949. 10.1002/sim.5764. 23440610

[52] Prevost TC , Abrams KR , Jones DR . Hierarchical models in generalized synthesis of evidence: an example based on studies of breast cancer screening. Stat Med. 2000;19(24):3359‐3376. 10.1002/1097-0258(20001230)19:24<3359::aid-sim710>3.0.co;2-n. 11122501

[53] Spiegelhalter DJ , Best NG , Carlin BP , Linde A . Bayesian measures of model complexity and fit. J Royal Stat Soc Ser B (Stat Methodol). 2002;64(4):583‐639. 10.1111/1467-9868.00353.

