# Supplementary material for: Assessing the performance of population adjustment methods for anchored indirect comparisons: A simulation study
Source: Stat Med. 2020 Oct 4;39(30):4885–911. doi: 10.1002/sim.8759 (PMC8690023; doi:10.1002/sim.8759)
Supplement: Supplementary file 1 — Data S1 Appendix B [file SIM-39-4885-s001.pdf]

B ADDITIONAL SIMULATION STUDY RESULTS

B.1 Scenario a

Figure B1 shows coverage zip plots[38] for the $d_{BC(AC)}$ contrast estimate, centred around zero bias. These display the 95% confidence or credible intervals for each repetition, centile-ranked on the vertical axis by

$$\left| \frac{(\hat{d}_i - d)}{\sqrt{\widehat{\text{var}}(\hat{d}_i)}} \right|, \tag{B1}$$

and coloured according to whether the interval includes the truth ("coverers", in green, at the bottom) or not ("non-coverers", in purple, at the top). The location of the colour change on the vertical axis is thus the estimated coverage (i.e. the proportion of coverers). If the intervals have nominal coverage, the colour change will occur at the 95th centile (i.e. at the nominal level). The horizontal dashed lines give the 95% Monte Carlo confidence interval for the coverage. The zip plot will appear symmetrical around zero (with a Y or zip shape) if the estimates are unbiased, otherwise the zip plot will be skewed to one side.

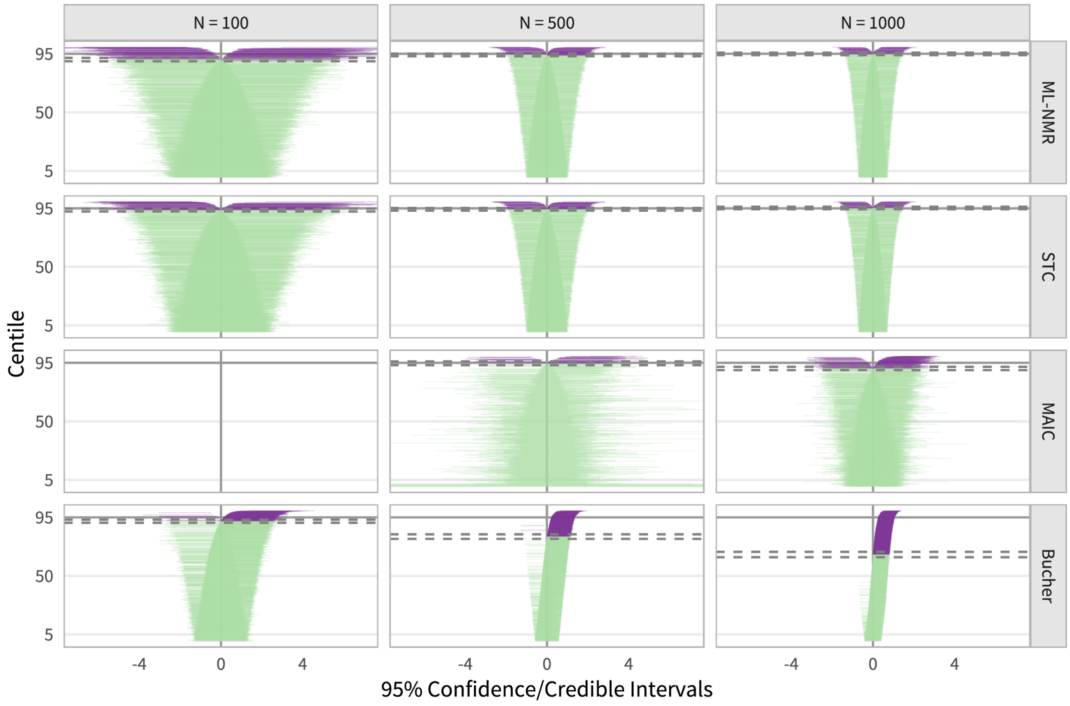

FIGURE B1 Coverage zip plots for the $d_{BC(AC)}$ contrast estimate for scenario a. Each method (other than Bucher) adjusts for the full set of effect modifiers. Sample size is varied between 100, 500, and 1000. The 95% confidence/credible intervals are coloured as coverers (green) or non-coverers (purple), and the colour change should occur at the 95th centile (i.e. nominal coverage). The horizontal dashed lines are 95% Monte Carlo confidence intervals for the coverage.

TABLE B1 Simulation results for scenario a, adjusting for all effect modifiers. Sample size N is varied between 100, 500, and 1000. Monte Carlo standard errors for each statistic are shown in brackets.

Method	Contrast	Scenario	Bias	Empirical SE	Model SE	Coverage
ML-NMR	$d_{AB(AB)}$	$N = 100$	-0.356 (0.013)	0.603 (0.010)	0.528 (0.002)	87.5 (0.7)
		$N = 500$	-0.056 (0.005)	0.215 (0.003)	0.207 (<0.001)	93.8 (0.5)
		$N = 1000$	-0.032 (0.003)	0.148 (0.002)	0.145 (<0.001)	94.4 (0.5)
	$d_{AC(AB)}$	$N = 100$	-0.286 (0.032)	1.420 (0.022)	1.219 (0.004)	91.0 (0.6)
		$N = 500$	-0.038 (0.011)	0.487 (0.008)	0.471 (<0.001)	94.6 (0.5)
		$N = 1000$	-0.005 (0.008)	0.336 (0.005)	0.328 (<0.001)	94.1 (0.5)
	$d_{BC(AB)}$	$N = 100$	0.070 (0.034)	1.513 (0.024)	1.301 (0.003)	90.5 (0.7)
		$N = 500$	0.019 (0.012)	0.522 (0.008)	0.507 (<0.001)	94.1 (0.5)
		$N = 1000$	0.026 (0.008)	0.361 (0.006)	0.354 (<0.001)	95.0 (0.5)
	$d_{AB(AC)}$	$N = 100$	-0.258 (0.032)	1.416 (0.022)	1.204 (0.004)	90.2 (0.7)
		$N = 500$	-0.041 (0.011)	0.474 (0.007)	0.462 (<0.001)	94.7 (0.5)
		$N = 1000$	-0.040 (0.007)	0.323 (0.005)	0.322 (<0.001)	95.0 (0.5)
	$d_{AC(AC)}$	$N = 100$	-0.188 (0.012)	0.542 (0.009)	0.517 (0.001)	93.1 (0.6)
		$N = 500$	-0.023 (0.005)	0.216 (0.003)	0.210 (<0.001)	94.3 (0.5)
		$N = 1000$	-0.014 (0.003)	0.153 (0.002)	0.148 (<0.001)	94.1 (0.5)
	$d_{BC(AC)}$	$N = 100$	0.070 (0.034)	1.513 (0.024)	1.301 (0.003)	90.5 (0.7)
		$N = 500$	0.019 (0.012)	0.522 (0.008)	0.507 (<0.001)	94.1 (0.5)
		$N = 1000$	0.026 (0.008)	0.361 (0.006)	0.354 (<0.001)	95.0 (0.5)
STC	$d_{AB(AC)}$	$N = 100$	-0.124 (0.028)	1.232 (0.019)	1.123 (0.003)	94.0 (0.5)
		$N = 500$	-0.020 (0.010)	0.463 (0.007)	0.457 (<0.001)	95.3 (0.5)
		$N = 1000$	-0.029 (0.007)	0.319 (0.005)	0.320 (<0.001)	95.2 (0.5)
	$d_{BC(AC)}$	$N = 100$	0.092 (0.030)	1.326 (0.021)	1.220 (0.003)	93.7 (0.5)
		$N = 500$	0.021 (0.011)	0.510 (0.008)	0.502 (<0.001)	94.2 (0.5)
		$N = 1000$	0.027 (0.008)	0.357 (0.006)	0.352 (<0.001)	95.2 (0.5)
MAIC	$d_{AB(AC)}$	$N = 100$	-3.215 (0.228)	10.203 (0.161)	- (-)	79.1 (0.9)
		$N = 500$	-0.199 (0.028)	1.249 (0.020)	- (-)	88.3 (0.7)
		$N = 1000$	-0.101 (0.015)	0.683 (0.011)	0.666 (0.005)	89.5 (0.7)
	$d_{BC(AC)}$	$N = 100$	3.176 (0.228)	10.214 (0.162)	- (-)	- (-)
		$N = 500$	0.200 (0.028)	1.260 (0.020)	- (-)	94.6 (0.5)
		$N = 1000$	0.099 (0.016)	0.699 (0.011)	0.682 (0.005)	90.7 (0.7)
Bucher	$d_{AB(AC)}$	$N = 100$	-0.359 (0.011)	0.475 (0.008)	0.461 (<0.001)	87.5 (0.7)
		$N = 500$	-0.316 (0.005)	0.207 (0.003)	0.202 (<0.001)	65.0 (1.1)
		$N = 1000$	-0.317 (0.003)	0.145 (0.002)	0.143 (<0.001)	39.7 (1.1)
	$d_{BC(AC)}$	$N = 100$	0.327 (0.015)	0.678 (0.011)	0.664 (<0.001)	92.0 (0.6)
		$N = 500$	0.318 (0.007)	0.295 (0.005)	0.290 (<0.001)	80.2 (0.9)
		$N = 1000$	0.315 (0.005)	0.210 (0.003)	0.205 (<0.001)	66.5 (1.1)

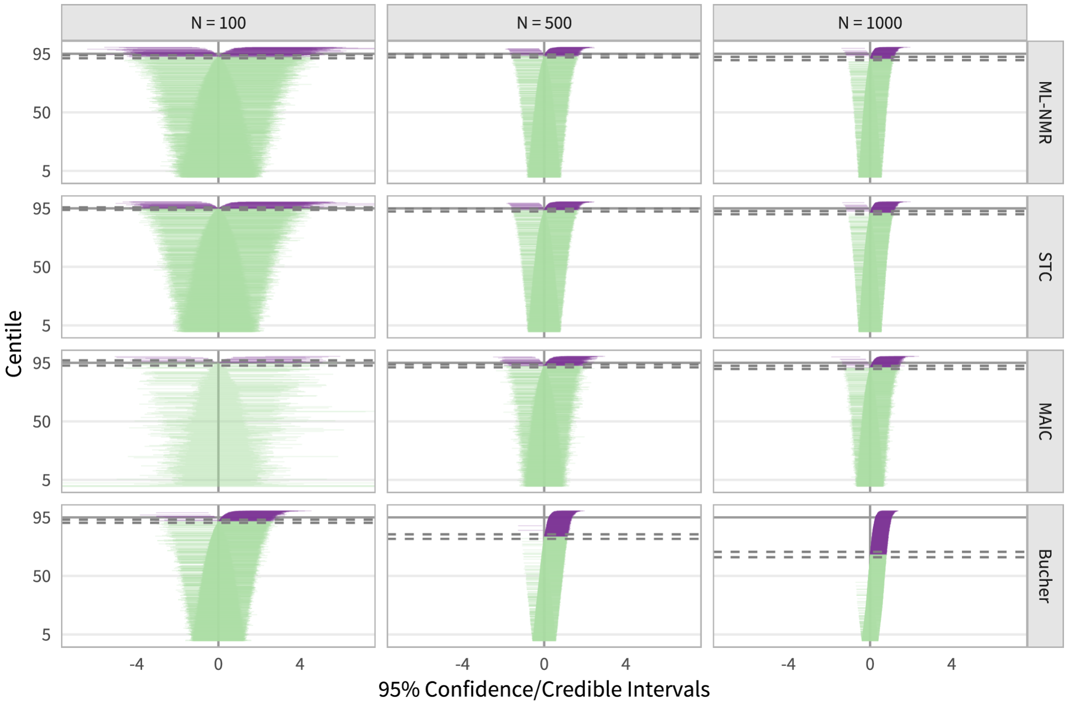

FIGURE B2 Coverage zip plots for the $d_{BC(AC)}$ contrast estimate for scenario a. One of the two effect modifiers was not adjusted for. Sample size is varied between 100, 500, and 1000. The 95% confidence/credible intervals are coloured as coverers (green) or non-coverers (purple), and the colour change should occur at the 95th centile (i.e. nominal coverage). The horizontal dashed lines are 95% Monte Carlo confidence intervals for the coverage.

TABLE B2 Simulation results for scenario a, only adjusting for one of two effect modifiers. Sample size N is varied between 100, 500, and 1000. Monte Carlo standard errors for each statistic are shown in brackets.

Method	Contrast	Scenario	Bias	Empirical SE	Model SE	Coverage
ML-NMR	$d_{AB(AB)}$	$N = 100$	-0.223 (0.012)	0.544 (0.009)	0.498 (0.001)	92.2 (0.6)
		$N = 500$	-0.035 (0.005)	0.212 (0.003)	0.205 (<0.001)	94.3 (0.5)
		$N = 1000$	-0.020 (0.003)	0.146 (0.002)	0.144 (<0.001)	94.4 (0.5)
	$d_{AC(AB)}$	$N = 100$	-0.025 (0.022)	0.962 (0.015)	0.892 (0.002)	93.2 (0.6)
		$N = 500$	0.115 (0.008)	0.370 (0.006)	0.358 (<0.001)	92.5 (0.6)
		$N = 1000$	0.140 (0.006)	0.258 (0.004)	0.251 (<0.001)	90.8 (0.6)
	$d_{BC(AB)}$	$N = 100$	0.198 (0.025)	1.098 (0.017)	1.004 (0.002)	92.7 (0.6)
		$N = 500$	0.150 (0.009)	0.416 (0.007)	0.408 (<0.001)	93.3 (0.6)
		$N = 1000$	0.160 (0.007)	0.293 (0.005)	0.286 (<0.001)	91.3 (0.6)
	$d_{AB(AC)}$	$N = 100$	-0.329 (0.022)	0.982 (0.016)	0.877 (0.003)	91.7 (0.6)
		$N = 500$	-0.165 (0.008)	0.353 (0.006)	0.351 (<0.001)	93.0 (0.6)
		$N = 1000$	-0.170 (0.006)	0.247 (0.004)	0.245 (<0.001)	90.1 (0.7)
	$d_{AC(AC)}$	$N = 100$	-0.131 (0.012)	0.519 (0.008)	0.502 (0.001)	93.8 (0.5)
		$N = 500$	-0.015 (0.005)	0.215 (0.003)	0.209 (<0.001)	94.2 (0.5)
		$N = 1000$	-0.010 (0.003)	0.152 (0.002)	0.147 (<0.001)	93.9 (0.5)
	$d_{BC(AC)}$	$N = 100$	0.198 (0.025)	1.098 (0.017)	1.004 (0.002)	92.7 (0.6)
		$N = 500$	0.150 (0.009)	0.416 (0.007)	0.408 (<0.001)	93.3 (0.6)
		$N = 1000$	0.160 (0.007)	0.293 (0.005)	0.286 (<0.001)	91.3 (0.6)
STC	$d_{AB(AC)}$	$N = 100$	-0.235 (0.020)	0.885 (0.014)	0.833 (0.003)	94.9 (0.5)
		$N = 500$	-0.149 (0.008)	0.347 (0.005)	0.347 (<0.001)	94.3 (0.5)
		$N = 1000$	-0.162 (0.005)	0.245 (0.004)	0.244 (<0.001)	91.0 (0.6)
	$d_{BC(AC)}$	$N = 100$	0.203 (0.022)	1.002 (0.016)	0.960 (0.002)	94.8 (0.5)
		$N = 500$	0.151 (0.009)	0.409 (0.006)	0.405 (<0.001)	93.7 (0.5)
		$N = 1000$	0.160 (0.007)	0.291 (0.005)	0.285 (<0.001)	91.6 (0.6)
MAIC	$d_{AB(AC)}$	$N = 100$	-0.454 (0.032)	1.417 (0.022)	- (-)	83.3 (0.8)
		$N = 500$	-0.169 (0.010)	0.439 (0.007)	0.444 (0.001)	91.0 (0.6)
		$N = 1000$	-0.178 (0.007)	0.311 (0.005)	0.303 (<0.001)	89.3 (0.7)
	$d_{BC(AC)}$	$N = 100$	0.422 (0.034)	1.498 (0.024)	- (-)	94.9 (0.5)
		$N = 500$	0.171 (0.011)	0.490 (0.008)	0.490 (0.001)	92.7 (0.6)
		$N = 1000$	0.176 (0.008)	0.346 (0.005)	0.337 (<0.001)	91.5 (0.6)
Bucher	$d_{AB(AC)}$	$N = 100$	-0.359 (0.011)	0.475 (0.008)	0.461 (<0.001)	87.5 (0.7)
		$N = 500$	-0.316 (0.005)	0.207 (0.003)	0.202 (<0.001)	65.0 (1.1)
		$N = 1000$	-0.317 (0.003)	0.145 (0.002)	0.143 (<0.001)	39.7 (1.1)
	$d_{BC(AC)}$	$N = 100$	0.327 (0.015)	0.678 (0.011)	0.664 (<0.001)	92.0 (0.6)
		$N = 500$	0.318 (0.007)	0.295 (0.005)	0.290 (<0.001)	80.2 (0.9)
		$N = 1000$	0.315 (0.005)	0.210 (0.003)	0.205 (<0.001)	66.5 (1.1)

B.2 Scenario b

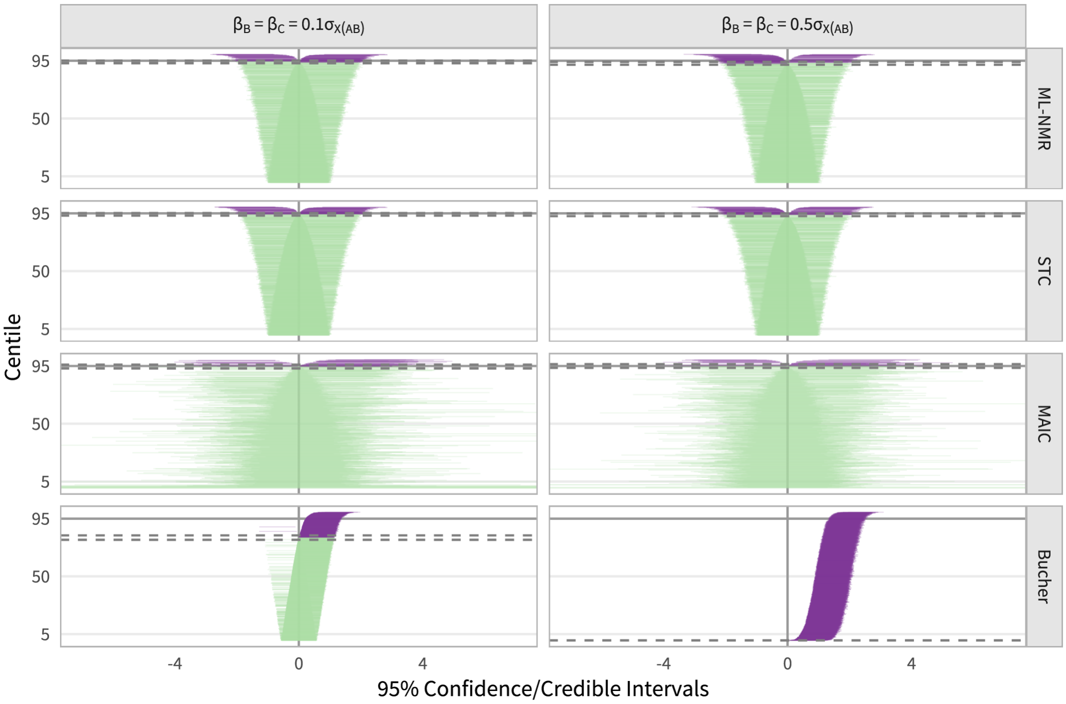

FIGURE B3 Coverage zip plots for the $d_{BC(AC)}$ contrast estimate for scenario b. Strength of effect modification is varied from weak (0.1 change in log odds ratio per covariate standard deviation in the AB study) to strong (0.5 change in log odds ratio per covariate standard deviation in the AB study). Each method (other than Bucher) adjusts for the full set of effect modifiers. The 95% confidence/credible intervals are coloured as coverers (green) or non-coverers (purple), and the colour change should occur at the 95th centile (i.e. nominal coverage). The horizontal dashed lines are 95% Monte Carlo confidence intervals for the coverage.

TABLE B3 Simulation results for scenario b, adjusting for all effect modifiers. Strength of effect modification is varied from weak to strong. Monte Carlo standard errors for each statistic are shown in brackets.

Method	Contrast	Scenario	Bias	Empirical SE	Model SE	Coverage
ML-NMR	$d_{AB(AB)}$	$\beta_B = \beta_C = 0.1\sigma_{X(AB)}$	-0.056 (0.005)	0.215 (0.003)	0.207 (<0.001)	93.8 (0.5)
		$\beta_B = \beta_C = 0.5\sigma_{X(AB)}$	-0.066 (0.005)	0.225 (0.004)	0.216 (<0.001)	93.2 (0.6)
	$d_{AC(AB)}$	$\beta_B = \beta_C = 0.1\sigma_{X(AB)}$	-0.038 (0.011)	0.487 (0.008)	0.471 (<0.001)	94.6 (0.5)
		$\beta_B = \beta_C = 0.5\sigma_{X(AB)}$	-0.137 (0.012)	0.545 (0.009)	0.503 (<0.001)	92.2 (0.6)
	$d_{BC(AB)}$	$\beta_B = \beta_C = 0.1\sigma_{X(AB)}$	0.019 (0.012)	0.522 (0.008)	0.507 (<0.001)	94.1 (0.5)
		$\beta_B = \beta_C = 0.5\sigma_{X(AB)}$	-0.071 (0.012)	0.545 (0.009)	0.516 (<0.001)	93.0 (0.6)
	$d_{AB(AC)}$	$\beta_B = \beta_C = 0.1\sigma_{X(AB)}$	-0.041 (0.011)	0.474 (0.007)	0.462 (<0.001)	94.7 (0.5)
		$\beta_B = \beta_C = 0.5\sigma_{X(AB)}$	0.037 (0.011)	0.501 (0.008)	0.470 (<0.001)	93.5 (0.5)
	$d_{AC(AC)}$	$\beta_B = \beta_C = 0.1\sigma_{X(AB)}$	-0.023 (0.005)	0.216 (0.003)	0.210 (<0.001)	94.3 (0.5)
		$\beta_B = \beta_C = 0.5\sigma_{X(AB)}$	-0.034 (0.005)	0.213 (0.003)	0.214 (<0.001)	94.8 (0.5)
	$d_{BC(AC)}$	$\beta_B = \beta_C = 0.1\sigma_{X(AB)}$	0.019 (0.012)	0.522 (0.008)	0.507 (<0.001)	94.1 (0.5)
		$\beta_B = \beta_C = 0.5\sigma_{X(AB)}$	-0.071 (0.012)	0.545 (0.009)	0.516 (<0.001)	93.0 (0.6)
STC	$d_{AB(AC)}$	$\beta_B = \beta_C = 0.1\sigma_{X(AB)}$	-0.020 (0.010)	0.463 (0.007)	0.457 (<0.001)	95.3 (0.5)
		$\beta_B = \beta_C = 0.5\sigma_{X(AB)}$	0.023 (0.011)	0.488 (0.008)	0.464 (<0.001)	94.2 (0.5)
	$d_{BC(AC)}$	$\beta_B = \beta_C = 0.1\sigma_{X(AB)}$	0.021 (0.011)	0.510 (0.008)	0.502 (<0.001)	94.2 (0.5)
		$\beta_B = \beta_C = 0.5\sigma_{X(AB)}$	-0.033 (0.012)	0.529 (0.008)	0.509 (<0.001)	93.9 (0.5)
MAIC	$d_{AB(AC)}$	$\beta_B = \beta_C = 0.1\sigma_{X(AB)}$	-0.199 (0.028)	1.249 (0.020)	- (-)	88.3 (0.7)
		$\beta_B = \beta_C = 0.5\sigma_{X(AB)}$	-0.128 (0.022)	0.968 (0.015)	- (-)	92.4 (0.6)
	$d_{BC(AC)}$	$\beta_B = \beta_C = 0.1\sigma_{X(AB)}$	0.200 (0.028)	1.260 (0.020)	- (-)	94.6 (0.5)
		$\beta_B = \beta_C = 0.5\sigma_{X(AB)}$	0.118 (0.022)	0.985 (0.016)	- (-)	95.0 (0.5)
Bucher	$d_{AB(AC)}$	$\beta_B = \beta_C = 0.1\sigma_{X(AB)}$	-0.316 (0.005)	0.207 (0.003)	0.202 (<0.001)	65.0 (1.1)
		$\beta_B = \beta_C = 0.5\sigma_{X(AB)}$	-1.468 (0.004)	0.200 (0.003)	0.200 (<0.001)	0.0 (0.0)
	$d_{BC(AC)}$	$\beta_B = \beta_C = 0.1\sigma_{X(AB)}$	0.318 (0.007)	0.295 (0.005)	0.290 (<0.001)	80.2 (0.9)
		$\beta_B = \beta_C = 0.5\sigma_{X(AB)}$	1.458 (0.006)	0.283 (0.004)	0.289 (<0.001)	<0.1 (<0.1)

TABLE B4 Simulation results for scenario b, only adjusting for one of two effect modifiers. Strength of effect modification is varied from weak to strong. Monte Carlo standard errors for each statistic are shown in brackets.

Method	Contrast	Scenario	Bias	Empirical SE	Model SE	Coverage
ML-NMR	$d_{AB(AB)}$	$\beta_B = \beta_C = 0.1\sigma_{X(AB)}$	-0.035 (0.005)	0.212 (0.003)	0.205 (<0.001)	94.3 (0.5)
		$\beta_B = \beta_C = 0.5\sigma_{X(AB)}$	0.006 (0.005)	0.213 (0.003)	0.209 (<0.001)	94.7 (0.5)
	$d_{AC(AB)}$	$\beta_B = \beta_C = 0.1\sigma_{X(AB)}$	0.115 (0.008)	0.370 (0.006)	0.358 (<0.001)	92.5 (0.6)
		$\beta_B = \beta_C = 0.5\sigma_{X(AB)}$	0.670 (0.008)	0.377 (0.006)	0.370 (<0.001)	56.4 (1.1)
	$d_{BC(AB)}$	$\beta_B = \beta_C = 0.1\sigma_{X(AB)}$	0.150 (0.009)	0.416 (0.007)	0.408 (<0.001)	93.3 (0.6)
		$\beta_B = \beta_C = 0.5\sigma_{X(AB)}$	0.664 (0.009)	0.402 (0.006)	0.405 (<0.001)	61.9 (1.1)
	$d_{AB(AC)}$	$\beta_B = \beta_C = 0.1\sigma_{X(AB)}$	-0.165 (0.008)	0.353 (0.006)	0.351 (<0.001)	93.0 (0.6)
		$\beta_B = \beta_C = 0.5\sigma_{X(AB)}$	-0.690 (0.008)	0.346 (0.005)	0.346 (<0.001)	46.9 (1.1)
	$d_{AC(AC)}$	$\beta_B = \beta_C = 0.1\sigma_{X(AB)}$	-0.015 (0.005)	0.215 (0.003)	0.209 (<0.001)	94.2 (0.5)
		$\beta_B = \beta_C = 0.5\sigma_{X(AB)}$	-0.026 (0.005)	0.210 (0.003)	0.212 (<0.001)	95.2 (0.5)
	$d_{BC(AC)}$	$\beta_B = \beta_C = 0.1\sigma_{X(AB)}$	0.150 (0.009)	0.416 (0.007)	0.408 (<0.001)	93.3 (0.6)
		$\beta_B = \beta_C = 0.5\sigma_{X(AB)}$	0.664 (0.009)	0.402 (0.006)	0.405 (<0.001)	61.9 (1.1)
STC	$d_{AB(AC)}$	$\beta_B = \beta_C = 0.1\sigma_{X(AB)}$	-0.149 (0.008)	0.347 (0.005)	0.347 (<0.001)	94.3 (0.5)
		$\beta_B = \beta_C = 0.5\sigma_{X(AB)}$	-0.690 (0.008)	0.339 (0.005)	0.343 (<0.001)	46.7 (1.1)
	$d_{BC(AC)}$	$\beta_B = \beta_C = 0.1\sigma_{X(AB)}$	0.151 (0.009)	0.409 (0.006)	0.405 (<0.001)	93.7 (0.5)
		$\beta_B = \beta_C = 0.5\sigma_{X(AB)}$	0.679 (0.009)	0.393 (0.006)	0.401 (<0.001)	59.8 (1.1)
MAIC	$d_{AB(AC)}$	$\beta_B = \beta_C = 0.1\sigma_{X(AB)}$	-0.169 (0.010)	0.439 (0.007)	0.444 (0.001)	91.0 (0.6)
		$\beta_B = \beta_C = 0.5\sigma_{X(AB)}$	-0.708 (0.009)	0.417 (0.007)	0.426 (0.001)	56.7 (1.1)
	$d_{BC(AC)}$	$\beta_B = \beta_C = 0.1\sigma_{X(AB)}$	0.171 (0.011)	0.490 (0.008)	0.490 (0.001)	92.7 (0.6)
		$\beta_B = \beta_C = 0.5\sigma_{X(AB)}$	0.698 (0.010)	0.456 (0.007)	0.474 (0.001)	69.4 (1.0)
Bucher	$d_{AB(AC)}$	$\beta_B = \beta_C = 0.1\sigma_{X(AB)}$	-0.316 (0.005)	0.207 (0.003)	0.202 (<0.001)	65.0 (1.1)
		$\beta_B = \beta_C = 0.5\sigma_{X(AB)}$	-1.468 (0.004)	0.200 (0.003)	0.200 (<0.001)	0.0 (0.0)
	$d_{BC(AC)}$	$\beta_B = \beta_C = 0.1\sigma_{X(AB)}$	0.318 (0.007)	0.295 (0.005)	0.290 (<0.001)	80.2 (0.9)
		$\beta_B = \beta_C = 0.5\sigma_{X(AB)}$	1.458 (0.006)	0.283 (0.004)	0.289 (<0.001)	<0.1 (<0.1)

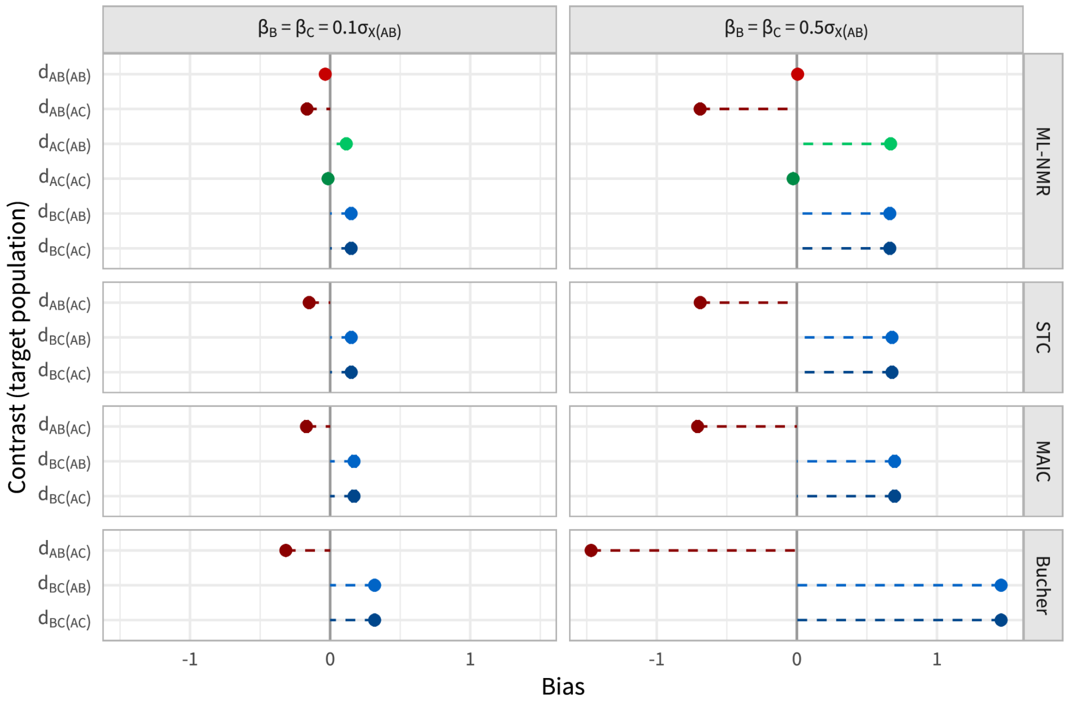

(a)

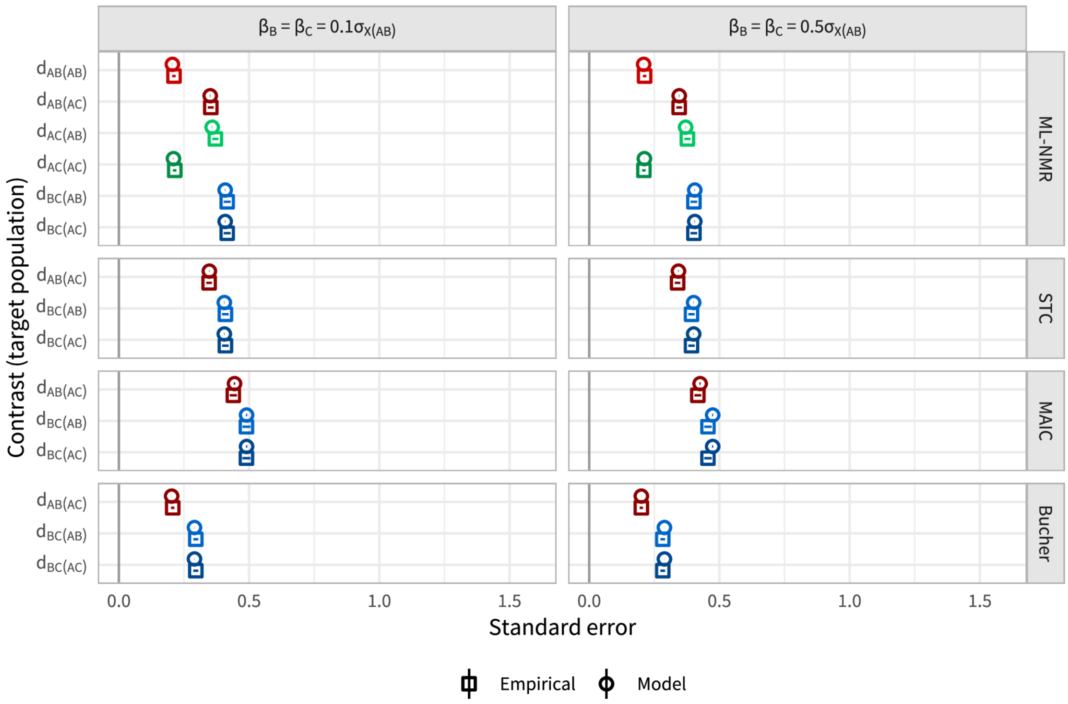

(b)

FIGURE B4 Bias (a) and standard errors (b) for the population-average contrast estimates for scenario b, along with 95% Monte Carlo confidence intervals. Strength of effect modification is varied from weak (0.1 change in log odds ratio per covariate standard deviation in the AB study) to strong (0.5 change in log odds ratio per covariate standard deviation in the AB study). One of the two effect modifiers was not adjusted for. The points are coloured by contrast, with lighter shades for the AB population and darker for the AC population.

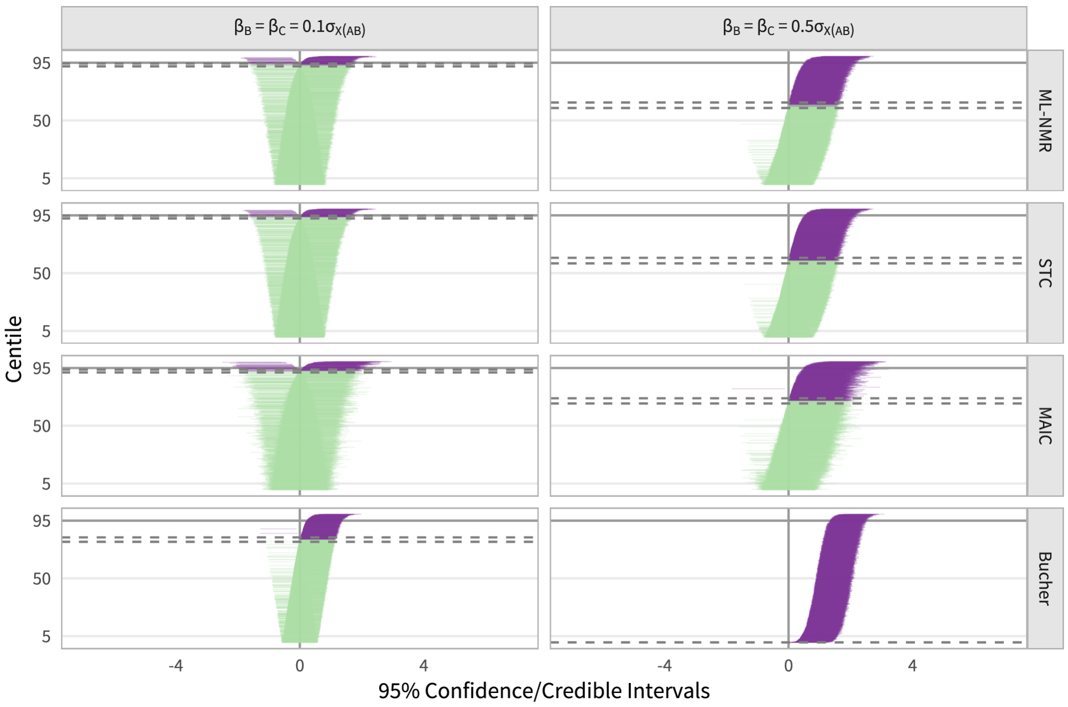

FIGURE B5 Coverage zip plots for the $d_{BC(AC)}$ contrast estimate for scenario b. Strength of effect modification is varied from weak (0.1 change in log odds ratio per covariate standard deviation in the AB study) to strong (0.5 change in log odds ratio per covariate standard deviation in the AB study). One of the two effect modifiers was not adjusted for. The 95% confidence/credible intervals are coloured as coverers (green) or non-coverers (purple), and the colour change should occur at the 95th centile (i.e. nominal coverage). The horizontal dashed lines are 95% Monte Carlo confidence intervals for the coverage.

B.3 Scenario c

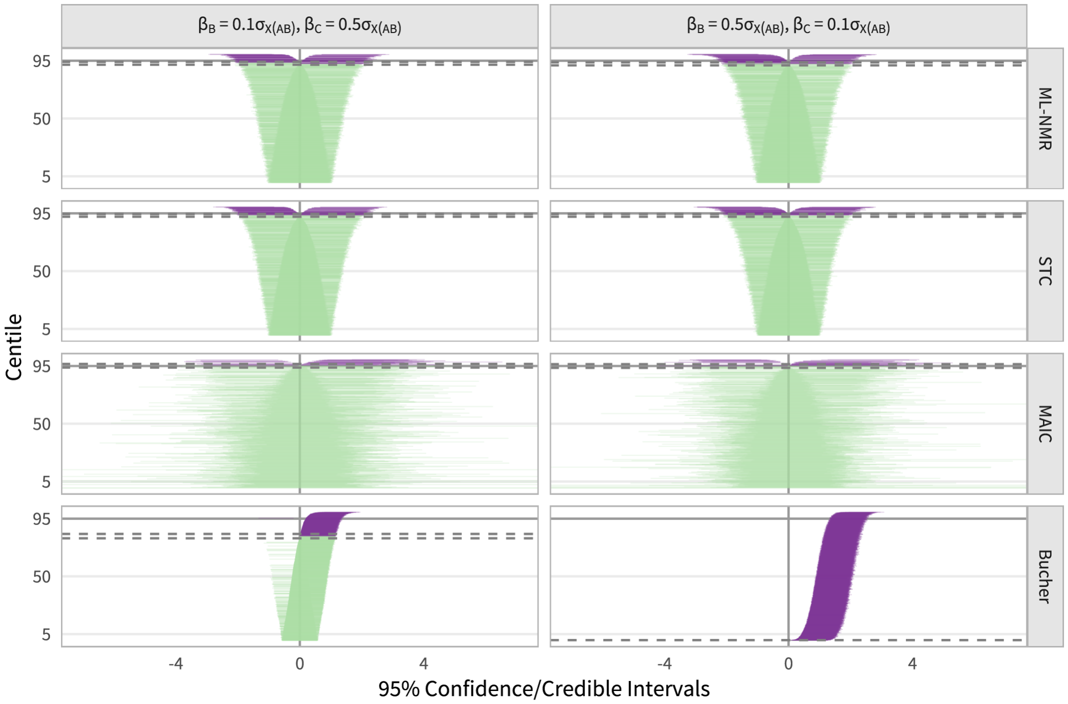

FIGURE B6 Coverage zip plots for the $d_{BC(AC)}$ contrast estimate for scenario c. Each method (other than Bucher) adjusts for the full set of effect modifiers. The shared effect modifier assumption is broken, so that treatment B is subject to weak effect modification whilst treatment C is subject to strong effect modification and vice versa. The 95% confidence/credible intervals are coloured as coverers (green) or non-coverers (purple), and the colour change should occur at the 95th centile (i.e. nominal coverage). The horizontal dashed lines are 95% Monte Carlo confidence intervals for the coverage.

TABLE B5 Simulation results for scenario c, adjusting for all effect modifiers. The shared effect modifier assumption is broken. Monte Carlo standard errors for each statistic are shown in brackets.

Method	Contrast	Scenario	Bias	Empirical SE	Model SE	Coverage
ML-NMR	$d_{AB(AB)}$	$\beta_B = 0.1\sigma_{X(AB)}, \beta_C = 0.5\sigma_{X(AB)}$	-0.061 (0.005)	0.213 (0.003)	0.207 (<0.001)	93.2 (0.6)
		$\beta_B = 0.5\sigma_{X(AB)}, \beta_C = 0.1\sigma_{X(AB)}$	-0.066 (0.005)	0.225 (0.004)	0.216 (<0.001)	93.1 (0.6)
	$d_{AC(AB)}$	$\beta_B = 0.1\sigma_{X(AB)}, \beta_C = 0.5\sigma_{X(AB)}$	1.192 (0.011)	0.502 (0.008)	0.472 (<0.001)	30.2 (1.0)
		$\beta_B = 0.5\sigma_{X(AB)}, \beta_C = 0.1\sigma_{X(AB)}$	-1.397 (0.012)	0.549 (0.009)	0.503 (<0.001)	19.8 (0.9)
	$d_{BC(AB)}$	$\beta_B = 0.1\sigma_{X(AB)}, \beta_C = 0.5\sigma_{X(AB)}$	1.253 (0.012)	0.537 (0.008)	0.508 (<0.001)	30.9 (1.0)
		$\beta_B = 0.5\sigma_{X(AB)}, \beta_C = 0.1\sigma_{X(AB)}$	-1.331 (0.012)	0.549 (0.009)	0.516 (<0.001)	27.2 (1.0)
	$d_{AB(AC)}$	$\beta_B = 0.1\sigma_{X(AB)}, \beta_C = 0.5\sigma_{X(AB)}$	-0.028 (0.011)	0.494 (0.008)	0.463 (<0.001)	93.3 (0.6)
		$\beta_B = 0.5\sigma_{X(AB)}, \beta_C = 0.1\sigma_{X(AB)}$	0.037 (0.011)	0.501 (0.008)	0.470 (<0.001)	93.5 (0.5)
	$d_{AC(AC)}$	$\beta_B = 0.1\sigma_{X(AB)}, \beta_C = 0.5\sigma_{X(AB)}$	-0.035 (0.005)	0.207 (0.003)	0.211 (<0.001)	94.8 (0.5)
		$\beta_B = 0.5\sigma_{X(AB)}, \beta_C = 0.1\sigma_{X(AB)}$	-0.034 (0.005)	0.216 (0.003)	0.214 (<0.001)	95.0 (0.5)
	$d_{BC(AC)}$	$\beta_B = 0.1\sigma_{X(AB)}, \beta_C = 0.5\sigma_{X(AB)}$	-0.007 (0.012)	0.537 (0.008)	0.508 (<0.001)	93.0 (0.6)
		$\beta_B = 0.5\sigma_{X(AB)}, \beta_C = 0.1\sigma_{X(AB)}$	-0.071 (0.012)	0.549 (0.009)	0.516 (<0.001)	92.6 (0.6)
STC	$d_{AB(AC)}$	$\beta_B = 0.1\sigma_{X(AB)}, \beta_C = 0.5\sigma_{X(AB)}$	-0.007 (0.011)	0.483 (0.008)	0.457 (<0.001)	93.5 (0.6)
		$\beta_B = 0.5\sigma_{X(AB)}, \beta_C = 0.1\sigma_{X(AB)}$	0.023 (0.011)	0.488 (0.008)	0.464 (<0.001)	94.2 (0.5)
	$d_{BC(AC)}$	$\beta_B = 0.1\sigma_{X(AB)}, \beta_C = 0.5\sigma_{X(AB)}$	-0.003 (0.012)	0.525 (0.008)	0.502 (<0.001)	93.5 (0.6)
		$\beta_B = 0.5\sigma_{X(AB)}, \beta_C = 0.1\sigma_{X(AB)}$	-0.033 (0.012)	0.531 (0.008)	0.509 (<0.001)	93.5 (0.6)
MAIC	$d_{AB(AC)}$	$\beta_B = 0.1\sigma_{X(AB)}, \beta_C = 0.5\sigma_{X(AB)}$	-0.212 (0.023)	1.029 (0.016)	- (-)	88.0 (0.7)
		$\beta_B = 0.5\sigma_{X(AB)}, \beta_C = 0.1\sigma_{X(AB)}$	-0.128 (0.022)	0.968 (0.015)	- (-)	92.4 (0.6)
	$d_{BC(AC)}$	$\beta_B = 0.1\sigma_{X(AB)}, \beta_C = 0.5\sigma_{X(AB)}$	0.202 (0.023)	1.047 (0.017)	- (-)	95.2 (0.5)
		$\beta_B = 0.5\sigma_{X(AB)}, \beta_C = 0.1\sigma_{X(AB)}$	0.118 (0.022)	0.987 (0.016)	- (-)	95.0 (0.5)
Bucher	$d_{AB(AC)}$	$\beta_B = 0.1\sigma_{X(AB)}, \beta_C = 0.5\sigma_{X(AB)}$	-0.320 (0.005)	0.205 (0.003)	0.202 (<0.001)	66.1 (1.1)
		$\beta_B = 0.5\sigma_{X(AB)}, \beta_C = 0.1\sigma_{X(AB)}$	-1.468 (0.004)	0.200 (0.003)	0.200 (<0.001)	0.0 (0.0)
	$d_{BC(AC)}$	$\beta_B = 0.1\sigma_{X(AB)}, \beta_C = 0.5\sigma_{X(AB)}$	0.309 (0.006)	0.286 (0.005)	0.290 (<0.001)	81.3 (0.9)
		$\beta_B = 0.5\sigma_{X(AB)}, \beta_C = 0.1\sigma_{X(AB)}$	1.459 (0.006)	0.283 (0.004)	0.289 (<0.001)	0.1 (<0.1)

TABLE B6 Simulation results for scenario c, only adjusting for one of two effect modifiers. The shared effect modifier assumption is broken. Monte Carlo standard errors for each statistic are shown in brackets.

Method	Contrast	Scenario	Bias	Empirical SE	Model SE	Coverage
ML-NMR	$d_{AB(AB)}$	$\beta_B = 0.1\sigma_{X(AB)}, \beta_C = 0.5\sigma_{X(AB)}$	-0.038 (0.005)	0.209 (0.003)	0.205 (<0.001)	93.9 (0.5)
		$\beta_B = 0.5\sigma_{X(AB)}, \beta_C = 0.1\sigma_{X(AB)}$	0.006 (0.005)	0.213 (0.003)	0.209 (<0.001)	95.0 (0.5)
	$d_{AC(AB)}$	$\beta_B = 0.1\sigma_{X(AB)}, \beta_C = 0.5\sigma_{X(AB)}$	1.355 (0.008)	0.368 (0.006)	0.359 (<0.001)	4.3 (0.5)
		$\beta_B = 0.5\sigma_{X(AB)}, \beta_C = 0.1\sigma_{X(AB)}$	-0.590 (0.009)	0.381 (0.006)	0.370 (<0.001)	64.4 (1.1)
	$d_{BC(AB)}$	$\beta_B = 0.1\sigma_{X(AB)}, \beta_C = 0.5\sigma_{X(AB)}$	1.394 (0.009)	0.414 (0.007)	0.409 (<0.001)	7.8 (0.6)
		$\beta_B = 0.5\sigma_{X(AB)}, \beta_C = 0.1\sigma_{X(AB)}$	-0.596 (0.009)	0.405 (0.006)	0.405 (<0.001)	68.3 (1.0)
	$d_{AB(AC)}$	$\beta_B = 0.1\sigma_{X(AB)}, \beta_C = 0.5\sigma_{X(AB)}$	-0.160 (0.008)	0.362 (0.006)	0.351 (<0.001)	92.0 (0.6)
		$\beta_B = 0.5\sigma_{X(AB)}, \beta_C = 0.1\sigma_{X(AB)}$	-0.690 (0.008)	0.346 (0.005)	0.346 (<0.001)	46.9 (1.1)
	$d_{AC(AC)}$	$\beta_B = 0.1\sigma_{X(AB)}, \beta_C = 0.5\sigma_{X(AB)}$	-0.026 (0.005)	0.206 (0.003)	0.210 (<0.001)	95.0 (0.5)
		$\beta_B = 0.5\sigma_{X(AB)}, \beta_C = 0.1\sigma_{X(AB)}$	-0.026 (0.005)	0.211 (0.003)	0.212 (<0.001)	95.0 (0.5)
	$d_{BC(AC)}$	$\beta_B = 0.1\sigma_{X(AB)}, \beta_C = 0.5\sigma_{X(AB)}$	0.134 (0.009)	0.414 (0.007)	0.409 (<0.001)	93.9 (0.5)
		$\beta_B = 0.5\sigma_{X(AB)}, \beta_C = 0.1\sigma_{X(AB)}$	0.664 (0.009)	0.405 (0.006)	0.405 (<0.001)	62.0 (1.1)
STC	$d_{AB(AC)}$	$\beta_B = 0.1\sigma_{X(AB)}, \beta_C = 0.5\sigma_{X(AB)}$	-0.145 (0.008)	0.355 (0.006)	0.348 (<0.001)	93.0 (0.6)
		$\beta_B = 0.5\sigma_{X(AB)}, \beta_C = 0.1\sigma_{X(AB)}$	-0.690 (0.008)	0.339 (0.005)	0.343 (<0.001)	46.7 (1.1)
	$d_{BC(AC)}$	$\beta_B = 0.1\sigma_{X(AB)}, \beta_C = 0.5\sigma_{X(AB)}$	0.134 (0.009)	0.407 (0.006)	0.405 (<0.001)	94.5 (0.5)
		$\beta_B = 0.5\sigma_{X(AB)}, \beta_C = 0.1\sigma_{X(AB)}$	0.680 (0.009)	0.396 (0.006)	0.401 (<0.001)	60.1 (1.1)
MAIC	$d_{AB(AC)}$	$\beta_B = 0.1\sigma_{X(AB)}, \beta_C = 0.5\sigma_{X(AB)}$	-0.163 (0.010)	0.441 (0.007)	0.445 (0.001)	91.1 (0.6)
		$\beta_B = 0.5\sigma_{X(AB)}, \beta_C = 0.1\sigma_{X(AB)}$	-0.708 (0.009)	0.417 (0.007)	0.426 (0.001)	56.7 (1.1)
	$d_{BC(AC)}$	$\beta_B = 0.1\sigma_{X(AB)}, \beta_C = 0.5\sigma_{X(AB)}$	0.153 (0.011)	0.479 (0.008)	0.491 (0.001)	93.9 (0.5)
		$\beta_B = 0.5\sigma_{X(AB)}, \beta_C = 0.1\sigma_{X(AB)}$	0.698 (0.010)	0.459 (0.007)	0.474 (0.001)	69.0 (1.0)
Bucher	$d_{AB(AC)}$	$\beta_B = 0.1\sigma_{X(AB)}, \beta_C = 0.5\sigma_{X(AB)}$	-0.320 (0.005)	0.205 (0.003)	0.202 (<0.001)	66.1 (1.1)
		$\beta_B = 0.5\sigma_{X(AB)}, \beta_C = 0.1\sigma_{X(AB)}$	-1.468 (0.004)	0.200 (0.003)	0.200 (<0.001)	0.0 (0.0)
	$d_{BC(AC)}$	$\beta_B = 0.1\sigma_{X(AB)}, \beta_C = 0.5\sigma_{X(AB)}$	0.309 (0.006)	0.286 (0.005)	0.290 (<0.001)	81.3 (0.9)
		$\beta_B = 0.5\sigma_{X(AB)}, \beta_C = 0.1\sigma_{X(AB)}$	1.459 (0.006)	0.283 (0.004)	0.289 (<0.001)	0.1 (<0.1)

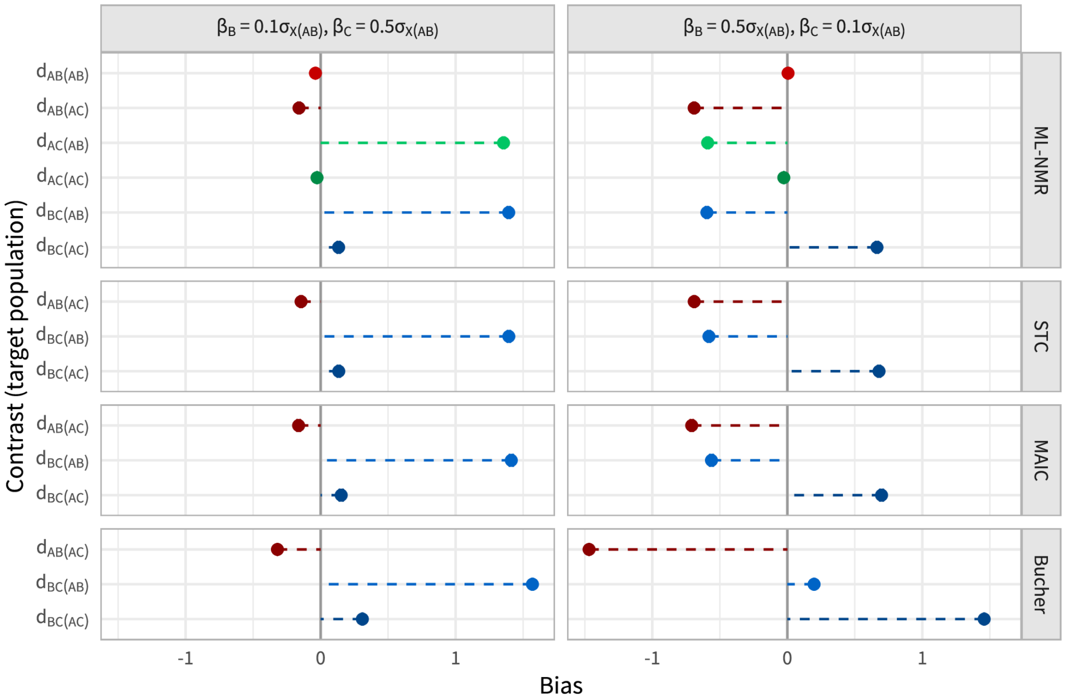

(a)

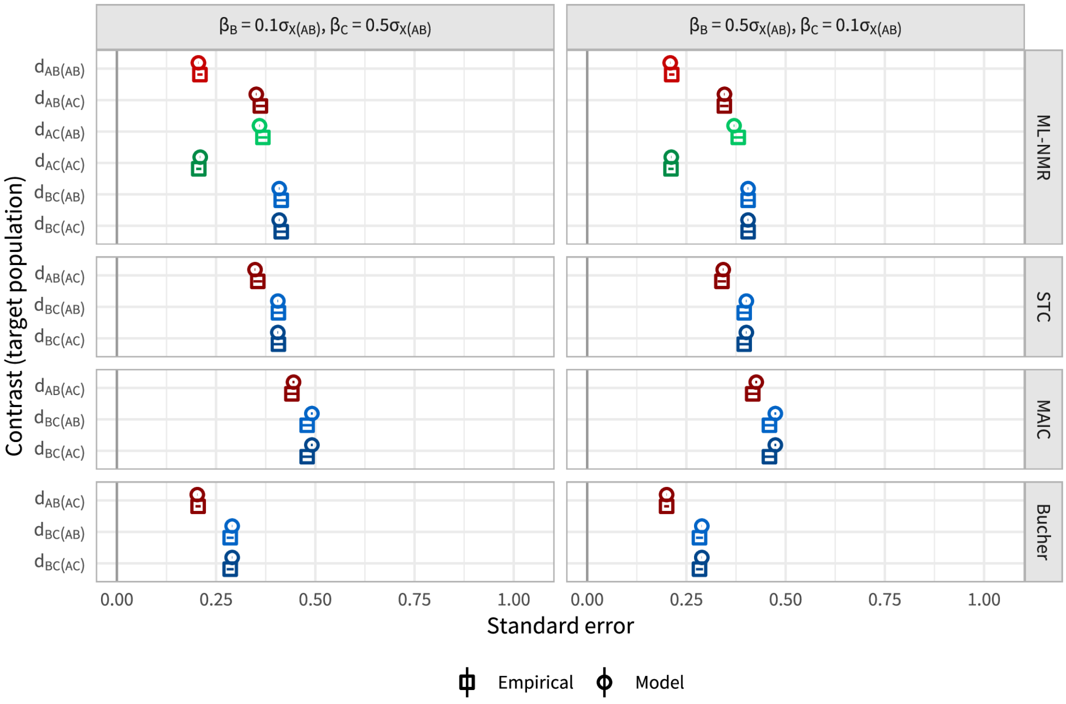

(b)

FIGURE B7 Bias (a) and standard errors (b) for the population-average contrast estimates for scenario c, along with 95% Monte Carlo confidence intervals. One of the two effect modifiers was not adjusted for. The shared effect modifier assumption is broken, so that treatment B is subject to weak effect modification whilst treatment C is subject to strong effect modification and vice versa. The points are coloured by contrast, with lighter shades for the AB population and darker for the AC population.

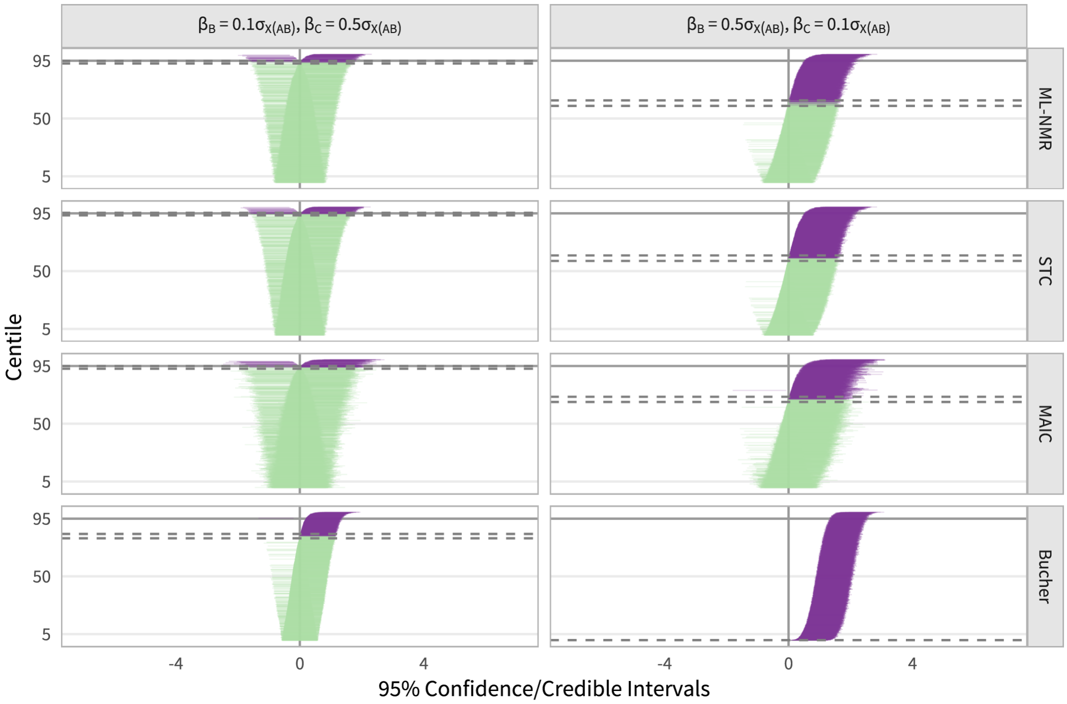

FIGURE B8 Coverage zip plots for the $d_{BC(AC)}$ contrast estimate for scenario c. One of the two effect modifiers was not adjusted for. The shared effect modifier assumption is broken, so that treatment B is subject to weak effect modification whilst treatment C is subject to strong effect modification and vice versa. The 95% confidence/credible intervals are coloured as coverers (green) or non-coverers (purple), and the colour change should occur at the 95th centile (i.e. nominal coverage). The horizontal dashed lines are 95% Monte Carlo confidence intervals for the coverage.

B.4 Scenario d

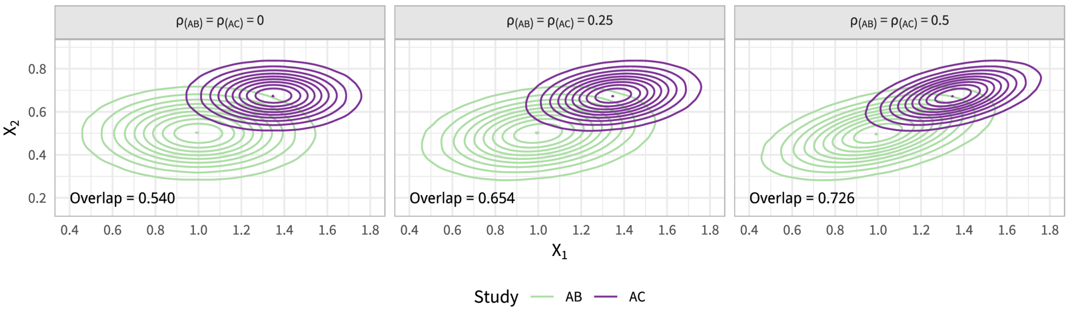

FIGURE B9 Joint covariate distributions in the AB and AC study, as the correlation between covariates is varied. The true overlap is defined as the proportion of the AC joint density contained within the 95% HDR of the AB joint density, calculated using numerical integration.

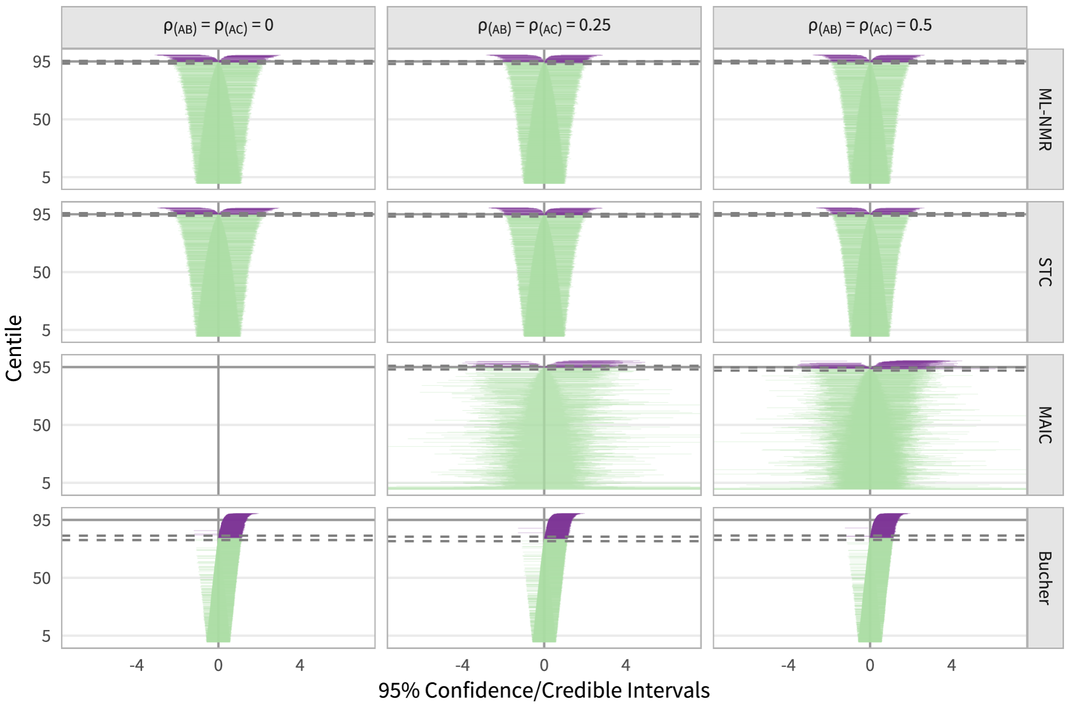

FIGURE B10 Coverage zip plots for the $d_{BC(AC)}$ contrast estimate for scenario d. The correlation between covariates is varied between 0, 0.25, and 0.5. Each method (other than Bucher) adjusts for the full set of effect modifiers. The 95% confidence/credible intervals are coloured as coverers (green) or non-coverers (purple), and the colour change should occur at the 95th centile (i.e. nominal coverage). The horizontal dashed lines are 95% Monte Carlo confidence intervals for the coverage.

TABLE B7 Simulation results for scenario d, adjusting for all effect modifiers. The correlation between covariates is varied between 0, 0.25, and 0.5. Monte Carlo standard errors for each statistic are shown in brackets.

Method	Contrast	Scenario	Bias	Empirical SE	Model SE	Coverage
ML-NMR	$d_{AB(AB)}$	$\rho_{(AB)} = \rho_{(AC)} = 0$	-0.059 (0.005)	0.211 (0.003)	0.207 (<0.001)	93.7 (0.5)
		$\rho_{(AB)} = \rho_{(AC)} = 0.25$	-0.056 (0.005)	0.215 (0.003)	0.207 (<0.001)	93.8 (0.5)
		$\rho_{(AB)} = \rho_{(AC)} = 0.5$	-0.059 (0.005)	0.212 (0.003)	0.207 (<0.001)	93.6 (0.5)
	$d_{AC(AB)}$	$\rho_{(AB)} = \rho_{(AC)} = 0$	-0.051 (0.012)	0.530 (0.008)	0.514 (<0.001)	94.5 (0.5)
		$\rho_{(AB)} = \rho_{(AC)} = 0.25$	-0.038 (0.011)	0.487 (0.008)	0.471 (<0.001)	94.6 (0.5)
		$\rho_{(AB)} = \rho_{(AC)} = 0.5$	-0.051 (0.010)	0.455 (0.007)	0.442 (<0.001)	94.0 (0.5)
	$d_{BC(AB)}$	$\rho_{(AB)} = \rho_{(AC)} = 0$	0.008 (0.013)	0.571 (0.009)	0.548 (<0.001)	94.3 (0.5)
		$\rho_{(AB)} = \rho_{(AC)} = 0.25$	0.019 (0.012)	0.522 (0.008)	0.507 (<0.001)	94.1 (0.5)
		$\rho_{(AB)} = \rho_{(AC)} = 0.5$	0.008 (0.011)	0.503 (0.008)	0.481 (<0.001)	94.5 (0.5)
	$d_{AB(AC)}$	$\rho_{(AB)} = \rho_{(AC)} = 0$	-0.045 (0.012)	0.524 (0.008)	0.506 (<0.001)	94.4 (0.5)
		$\rho_{(AB)} = \rho_{(AC)} = 0.25$	-0.041 (0.011)	0.474 (0.007)	0.462 (<0.001)	94.7 (0.5)
		$\rho_{(AB)} = \rho_{(AC)} = 0.5$	-0.046 (0.010)	0.449 (0.007)	0.432 (<0.001)	94.4 (0.5)
	$d_{AC(AC)}$	$\rho_{(AB)} = \rho_{(AC)} = 0$	-0.037 (0.005)	0.211 (0.003)	0.211 (<0.001)	94.4 (0.5)
		$\rho_{(AB)} = \rho_{(AC)} = 0.25$	-0.023 (0.005)	0.216 (0.003)	0.210 (<0.001)	94.3 (0.5)
		$\rho_{(AB)} = \rho_{(AC)} = 0.5$	-0.038 (0.005)	0.211 (0.003)	0.211 (<0.001)	94.5 (0.5)
	$d_{BC(AC)}$	$\rho_{(AB)} = \rho_{(AC)} = 0$	0.008 (0.013)	0.571 (0.009)	0.548 (<0.001)	94.3 (0.5)
		$\rho_{(AB)} = \rho_{(AC)} = 0.25$	0.019 (0.012)	0.522 (0.008)	0.507 (<0.001)	94.1 (0.5)
		$\rho_{(AB)} = \rho_{(AC)} = 0.5$	0.008 (0.011)	0.503 (0.008)	0.481 (<0.001)	94.5 (0.5)
STC	$d_{AB(AC)}$	$\rho_{(AB)} = \rho_{(AC)} = 0$	-0.024 (0.011)	0.511 (0.008)	0.500 (<0.001)	95.2 (0.5)
		$\rho_{(AB)} = \rho_{(AC)} = 0.25$	-0.020 (0.010)	0.463 (0.007)	0.457 (<0.001)	95.3 (0.5)
		$\rho_{(AB)} = \rho_{(AC)} = 0.5$	-0.025 (0.010)	0.439 (0.007)	0.427 (<0.001)	95.2 (0.5)
	$d_{BC(AC)}$	$\rho_{(AB)} = \rho_{(AC)} = 0$	0.011 (0.012)	0.557 (0.009)	0.542 (<0.001)	94.8 (0.5)
		$\rho_{(AB)} = \rho_{(AC)} = 0.25$	0.021 (0.011)	0.510 (0.008)	0.502 (<0.001)	94.2 (0.5)
		$\rho_{(AB)} = \rho_{(AC)} = 0.5$	0.011 (0.011)	0.492 (0.008)	0.475 (<0.001)	94.8 (0.5)
MAIC	$d_{AB(AC)}$	$\rho_{(AB)} = \rho_{(AC)} = 0$	-0.621 (0.067)	2.975 (0.047)	- (-)	87.6 (0.7)
		$\rho_{(AB)} = \rho_{(AC)} = 0.25$	-0.199 (0.028)	1.249 (0.020)	- (-)	88.3 (0.7)
		$\rho_{(AB)} = \rho_{(AC)} = 0.5$	-0.139 (0.018)	0.792 (0.013)	- (-)	90.8 (0.6)
	$d_{BC(AC)}$	$\rho_{(AB)} = \rho_{(AC)} = 0$	0.609 (0.067)	2.991 (0.047)	- (-)	100.0 (0.0)
		$\rho_{(AB)} = \rho_{(AC)} = 0.25$	0.200 (0.028)	1.260 (0.020)	- (-)	94.6 (0.5)
		$\rho_{(AB)} = \rho_{(AC)} = 0.5$	0.126 (0.019)	0.828 (0.013)	- (-)	93.4 (0.6)
Bucher	$d_{AB(AC)}$	$\rho_{(AB)} = \rho_{(AC)} = 0$	-0.320 (0.005)	0.203 (0.003)	0.202 (<0.001)	65.5 (1.1)
		$\rho_{(AB)} = \rho_{(AC)} = 0.25$	-0.316 (0.005)	0.207 (0.003)	0.202 (<0.001)	65.0 (1.1)
		$\rho_{(AB)} = \rho_{(AC)} = 0.5$	-0.317 (0.005)	0.204 (0.003)	0.202 (<0.001)	65.8 (1.1)
	$d_{BC(AC)}$	$\rho_{(AB)} = \rho_{(AC)} = 0$	0.307 (0.007)	0.296 (0.005)	0.290 (<0.001)	81.0 (0.9)
		$\rho_{(AB)} = \rho_{(AC)} = 0.25$	0.318 (0.007)	0.295 (0.005)	0.290 (<0.001)	80.2 (0.9)
		$\rho_{(AB)} = \rho_{(AC)} = 0.5$	0.304 (0.007)	0.298 (0.005)	0.290 (<0.001)	81.2 (0.9)

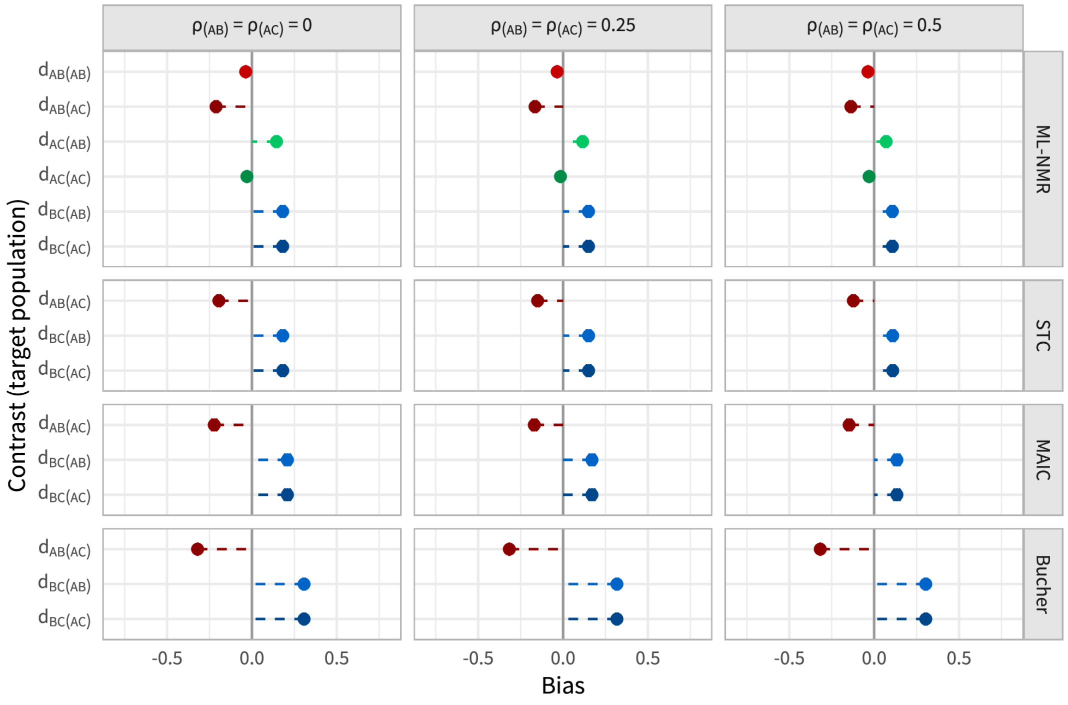

(a)

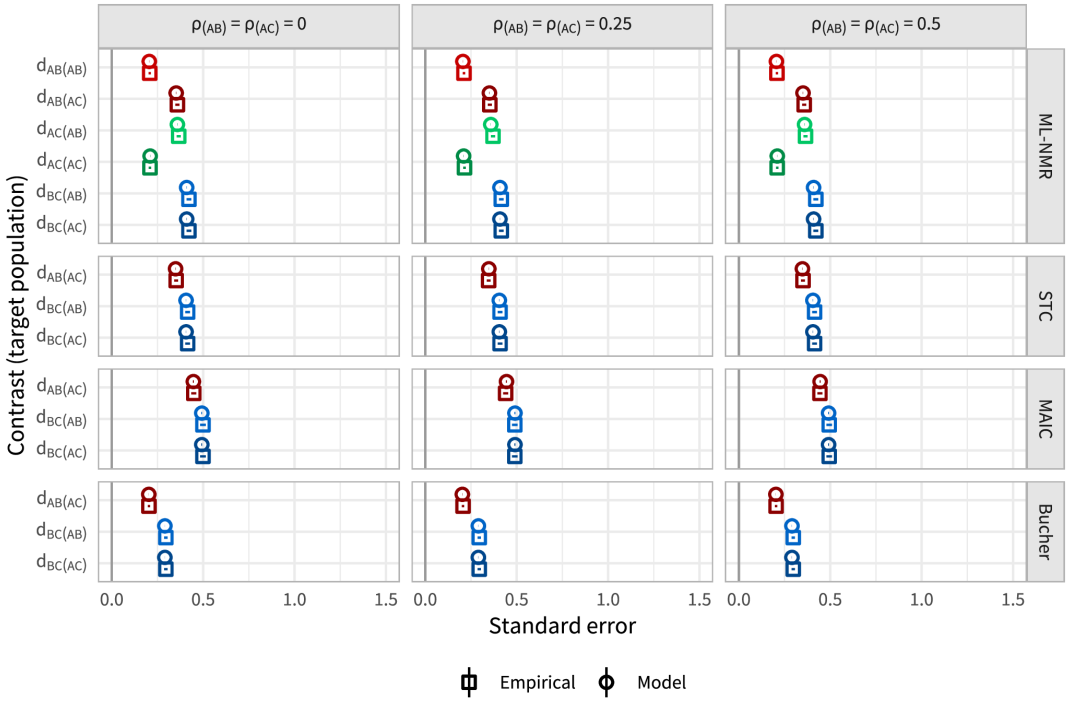

(b)

FIGURE B11 Bias (a) and standard errors (b) for the population-average contrast estimates for scenario d, along with 95% Monte Carlo confidence intervals. The correlation between covariates is varied between 0, 0.25, and 0.5. One of the two effect modifiers was not adjusted for. The points are coloured by contrast, with lighter shades for the AB population and darker for the AC population.

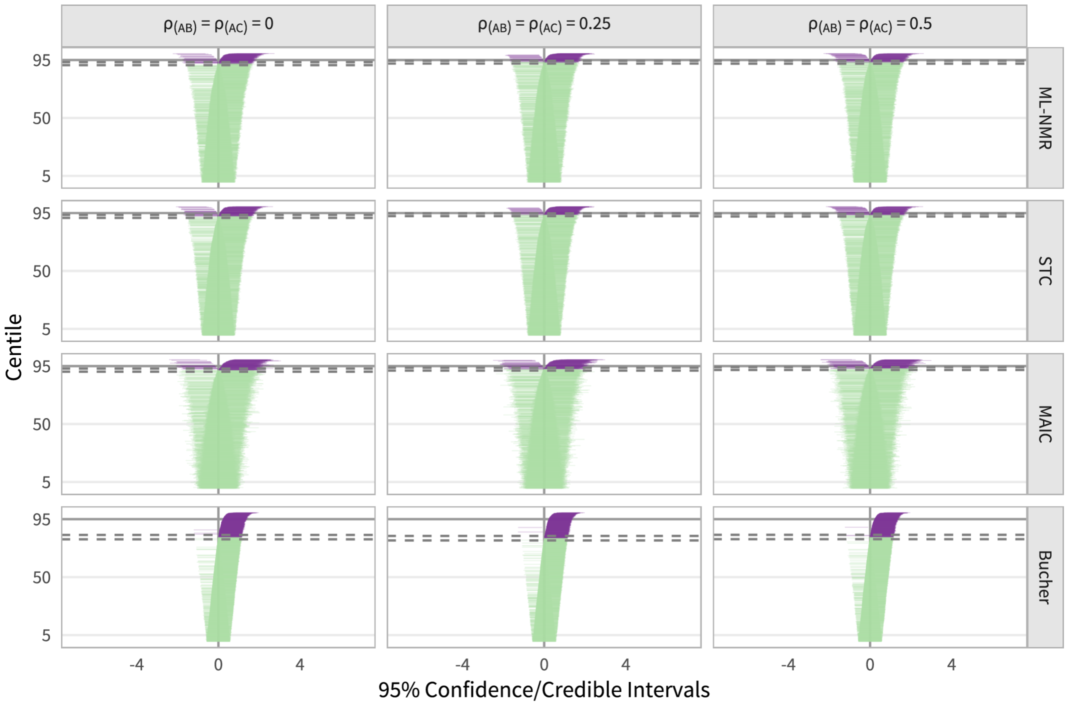

FIGURE B12 Coverage zip plots for the $d_{BC(AC)}$ contrast estimate for scenario d. The correlation between covariates is varied between 0, 0.25, and 0.5. One of the two effect modifiers was not adjusted for. The 95% confidence/credible intervals are coloured as coverers (green) or non-coverers (purple), and the colour change should occur at the 95th centile (i.e. nominal coverage). The horizontal dashed lines are 95% Monte Carlo confidence intervals for the coverage.

TABLE B8 Simulation results for scenario d, only adjusting for one of two effect modifiers. The correlation between covariates is varied between 0, 0.25, and 0.5. Monte Carlo standard errors for each statistic are shown in brackets.

Method	Contrast	Scenario	Bias	Empirical SE	Model SE	Coverage
ML-NMR	$d_{AB(AB)}$	$\rho_{(AB)} = \rho_{(AC)} = 0$	-0.037 (0.005)	0.208 (0.003)	0.205 (<0.001)	94.1 (0.5)
		$\rho_{(AB)} = \rho_{(AC)} = 0.25$	-0.035 (0.005)	0.212 (0.003)	0.205 (<0.001)	94.3 (0.5)
		$\rho_{(AB)} = \rho_{(AC)} = 0.5$	-0.037 (0.005)	0.208 (0.003)	0.205 (<0.001)	94.2 (0.5)
	$d_{AC(AB)}$	$\rho_{(AB)} = \rho_{(AC)} = 0$	0.145 (0.008)	0.366 (0.006)	0.359 (<0.001)	92.2 (0.6)
		$\rho_{(AB)} = \rho_{(AC)} = 0.25$	0.115 (0.008)	0.370 (0.006)	0.358 (<0.001)	92.5 (0.6)
		$\rho_{(AB)} = \rho_{(AC)} = 0.5$	0.071 (0.008)	0.365 (0.006)	0.359 (<0.001)	94.1 (0.5)
	$d_{BC(AB)}$	$\rho_{(AB)} = \rho_{(AC)} = 0$	0.182 (0.009)	0.422 (0.007)	0.410 (<0.001)	92.2 (0.6)
		$\rho_{(AB)} = \rho_{(AC)} = 0.25$	0.150 (0.009)	0.416 (0.007)	0.408 (<0.001)	93.3 (0.6)
		$\rho_{(AB)} = \rho_{(AC)} = 0.5$	0.107 (0.009)	0.421 (0.007)	0.408 (<0.001)	93.2 (0.6)
	$d_{AB(AC)}$	$\rho_{(AB)} = \rho_{(AC)} = 0$	-0.211 (0.008)	0.359 (0.006)	0.352 (<0.001)	90.3 (0.7)
		$\rho_{(AB)} = \rho_{(AC)} = 0.25$	-0.165 (0.008)	0.353 (0.006)	0.351 (<0.001)	93.0 (0.6)
		$\rho_{(AB)} = \rho_{(AC)} = 0.5$	-0.137 (0.008)	0.357 (0.006)	0.351 (<0.001)	92.8 (0.6)
	$d_{AC(AC)}$	$\rho_{(AB)} = \rho_{(AC)} = 0$	-0.029 (0.005)	0.209 (0.003)	0.210 (<0.001)	94.7 (0.5)
		$\rho_{(AB)} = \rho_{(AC)} = 0.25$	-0.015 (0.005)	0.215 (0.003)	0.209 (<0.001)	94.2 (0.5)
		$\rho_{(AB)} = \rho_{(AC)} = 0.5$	-0.030 (0.005)	0.210 (0.003)	0.210 (<0.001)	94.5 (0.5)
	$d_{BC(AC)}$	$\rho_{(AB)} = \rho_{(AC)} = 0$	0.182 (0.009)	0.422 (0.007)	0.410 (<0.001)	92.2 (0.6)
		$\rho_{(AB)} = \rho_{(AC)} = 0.25$	0.150 (0.009)	0.416 (0.007)	0.408 (<0.001)	93.3 (0.6)
		$\rho_{(AB)} = \rho_{(AC)} = 0.5$	0.107 (0.009)	0.421 (0.007)	0.408 (<0.001)	93.2 (0.6)
STC	$d_{AB(AC)}$	$\rho_{(AB)} = \rho_{(AC)} = 0$	-0.195 (0.008)	0.352 (0.006)	0.349 (<0.001)	91.4 (0.6)
		$\rho_{(AB)} = \rho_{(AC)} = 0.25$	-0.149 (0.008)	0.347 (0.005)	0.347 (<0.001)	94.3 (0.5)
		$\rho_{(AB)} = \rho_{(AC)} = 0.5$	-0.122 (0.008)	0.351 (0.006)	0.347 (<0.001)	93.7 (0.5)
	$d_{BC(AC)}$	$\rho_{(AB)} = \rho_{(AC)} = 0$	0.182 (0.009)	0.415 (0.007)	0.406 (<0.001)	92.4 (0.6)
		$\rho_{(AB)} = \rho_{(AC)} = 0.25$	0.151 (0.009)	0.409 (0.006)	0.405 (<0.001)	93.7 (0.5)
		$\rho_{(AB)} = \rho_{(AC)} = 0.5$	0.109 (0.009)	0.414 (0.007)	0.405 (<0.001)	93.4 (0.6)
MAIC	$d_{AB(AC)}$	$\rho_{(AB)} = \rho_{(AC)} = 0$	-0.222 (0.010)	0.449 (0.007)	0.446 (0.001)	88.3 (0.7)
		$\rho_{(AB)} = \rho_{(AC)} = 0.25$	-0.169 (0.010)	0.439 (0.007)	0.444 (0.001)	91.0 (0.6)
		$\rho_{(AB)} = \rho_{(AC)} = 0.5$	-0.147 (0.010)	0.444 (0.007)	0.444 (0.001)	91.1 (0.6)
	$d_{BC(AC)}$	$\rho_{(AB)} = \rho_{(AC)} = 0$	0.209 (0.011)	0.499 (0.008)	0.492 (0.001)	91.8 (0.6)
		$\rho_{(AB)} = \rho_{(AC)} = 0.25$	0.171 (0.011)	0.490 (0.008)	0.490 (0.001)	92.7 (0.6)
		$\rho_{(AB)} = \rho_{(AC)} = 0.5$	0.134 (0.011)	0.494 (0.008)	0.491 (0.001)	93.1 (0.6)
Bucher	$d_{AB(AC)}$	$\rho_{(AB)} = \rho_{(AC)} = 0$	-0.320 (0.005)	0.203 (0.003)	0.202 (<0.001)	65.5 (1.1)
		$\rho_{(AB)} = \rho_{(AC)} = 0.25$	-0.316 (0.005)	0.207 (0.003)	0.202 (<0.001)	65.0 (1.1)
		$\rho_{(AB)} = \rho_{(AC)} = 0.5$	-0.317 (0.005)	0.204 (0.003)	0.202 (<0.001)	65.8 (1.1)
	$d_{BC(AC)}$	$\rho_{(AB)} = \rho_{(AC)} = 0$	0.307 (0.007)	0.296 (0.005)	0.290 (<0.001)	81.0 (0.9)
		$\rho_{(AB)} = \rho_{(AC)} = 0.25$	0.318 (0.007)	0.295 (0.005)	0.290 (<0.001)	80.2 (0.9)
		$\rho_{(AB)} = \rho_{(AC)} = 0.5$	0.304 (0.007)	0.298 (0.005)	0.290 (<0.001)	81.2 (0.9)

B.5 Scenarios e and f

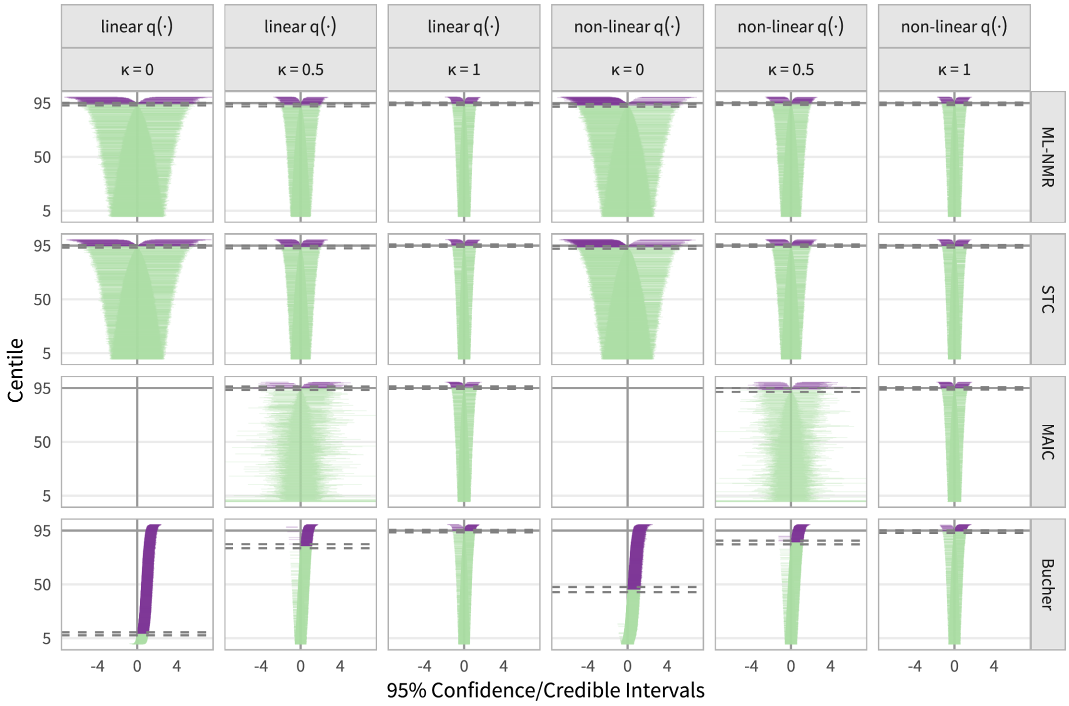

FIGURE B13 Coverage zip plots for the $d_{BC(AC)}$ contrast estimate for scenarios e and f. Each method (other than Bucher) adjusts for the full set of effect modifiers. The between-study overlap and covariate-outcome relationship are varied jointly. The 95% confidence/credible intervals are coloured as coverers (green) or non-coverers (purple), and the colour change should occur at the 95th centile (i.e. nominal coverage). The horizontal dashed lines are 95% Monte Carlo confidence intervals for the coverage.

TABLE B9 Simulation results for scenarios e and f, adjusting for all effect modifiers. The between-study overlap and covariate-outcome relationship are varied jointly. Monte Carlo standard errors for each statistic are shown in brackets.

Method	Contrast	Scenario		Bias	Empirical SE	Model SE	Coverage
ML-NMR	$d_{AB(AB)}$	$\kappa = 0$	linear $q(\cdot)$	-0.050 (0.005)	0.212 (0.003)	0.207 (<0.001)	93.3 (0.6)
		$\kappa = 0$	non-linear $q(\cdot)$	-0.018 (0.004)	0.197 (0.003)	0.197 (<0.001)	94.7 (0.5)
		$\kappa = 0.5$	linear $q(\cdot)$	-0.050 (0.005)	0.212 (0.003)	0.207 (<0.001)	93.4 (0.6)
		$\kappa = 0.5$	non-linear $q(\cdot)$	-0.018 (0.004)	0.197 (0.003)	0.197 (<0.001)	94.5 (0.5)
		$\kappa = 1$	linear $q(\cdot)$	-0.050 (0.005)	0.212 (0.003)	0.207 (<0.001)	93.8 (0.5)
		$\kappa = 1$	non-linear $q(\cdot)$	-0.018 (0.004)	0.198 (0.003)	0.196 (<0.001)	94.5 (0.5)
	$d_{AC(AB)}$	$\kappa = 0$	linear $q(\cdot)$	-0.083 (0.031)	1.394 (0.022)	1.338 (0.001)	94.0 (0.5)
		$\kappa = 0$	non-linear $q(\cdot)$	-0.317 (0.029)	1.319 (0.021)	1.280 (0.001)	93.0 (0.6)
		$\kappa = 0.5$	linear $q(\cdot)$	-0.052 (0.011)	0.488 (0.008)	0.470 (<0.001)	93.7 (0.5)
		$\kappa = 0.5$	non-linear $q(\cdot)$	-0.029 (0.011)	0.476 (0.008)	0.464 (<0.001)	94.2 (0.5)
		$\kappa = 1$	linear $q(\cdot)$	-0.042 (0.006)	0.247 (0.004)	0.243 (<0.001)	94.5 (0.5)
		$\kappa = 1$	non-linear $q(\cdot)$	-0.011 (0.006)	0.264 (0.004)	0.260 (<0.001)	94.8 (0.5)
	$d_{BC(AB)}$	$\kappa = 0$	linear $q(\cdot)$	-0.033 (0.032)	1.409 (0.022)	1.346 (0.001)	94.1 (0.5)
		$\kappa = 0$	non-linear $q(\cdot)$	-0.299 (0.030)	1.338 (0.021)	1.297 (0.001)	93.0 (0.6)
		$\kappa = 0.5$	linear $q(\cdot)$	-0.002 (0.012)	0.532 (0.008)	0.507 (<0.001)	93.3 (0.6)
		$\kappa = 0.5$	non-linear $q(\cdot)$	-0.011 (0.012)	0.520 (0.008)	0.506 (<0.001)	94.5 (0.5)
		$\kappa = 1$	linear $q(\cdot)$	0.008 (0.007)	0.326 (0.005)	0.316 (<0.001)	94.3 (0.5)
		$\kappa = 1$	non-linear $q(\cdot)$	0.007 (0.007)	0.331 (0.005)	0.326 (<0.001)	94.4 (0.5)
	$d_{AB(AC)}$	$\kappa = 0$	linear $q(\cdot)$	-0.006 (0.031)	1.390 (0.022)	1.330 (0.001)	94.0 (0.5)
		$\kappa = 0$	non-linear $q(\cdot)$	0.315 (0.029)	1.313 (0.021)	1.276 (0.001)	92.8 (0.6)
		$\kappa = 0.5$	linear $q(\cdot)$	-0.036 (0.011)	0.485 (0.008)	0.462 (<0.001)	93.5 (0.5)

TABLE B9 (continued)

Method	Contrast	Scenario		Bias	Empirical SE	Model SE	Coverage
		$\kappa = 0.5$	non-linear $q(\cdot)$	0.009 (0.010)	0.460 (0.007)	0.449 (<0.001)	94.0 (0.5)
		$\kappa = 1$	linear $q(\cdot)$	-0.046 (0.006)	0.246 (0.004)	0.236 (<0.001)	93.2 (0.6)
		$\kappa = 1$	non-linear $q(\cdot)$	-0.014 (0.005)	0.233 (0.004)	0.229 (<0.001)	94.6 (0.5)
	$d_{AC(AC)}$	$\kappa = 0$	linear $q(\cdot)$	-0.038 (0.005)	0.210 (0.003)	0.211 (<0.001)	95.0 (0.5)
		$\kappa = 0$	non-linear $q(\cdot)$	0.016 (0.006)	0.266 (0.004)	0.265 (<0.001)	94.7 (0.5)
		$\kappa = 0.5$	linear $q(\cdot)$	-0.038 (0.005)	0.210 (0.003)	0.211 (<0.001)	94.7 (0.5)
		$\kappa = 0.5$	non-linear $q(\cdot)$	-0.002 (0.005)	0.245 (0.004)	0.244 (<0.001)	95.0 (0.5)
		$\kappa = 1$	linear $q(\cdot)$	-0.038 (0.005)	0.210 (0.003)	0.211 (<0.001)	95.0 (0.5)
		$\kappa = 1$	non-linear $q(\cdot)$	-0.007 (0.005)	0.237 (0.004)	0.236 (<0.001)	94.9 (0.5)
	$d_{BC(AC)}$	$\kappa = 0$	linear $q(\cdot)$	-0.033 (0.032)	1.409 (0.022)	1.346 (0.001)	94.1 (0.5)
		$\kappa = 0$	non-linear $q(\cdot)$	-0.299 (0.030)	1.338 (0.021)	1.297 (0.001)	93.0 (0.6)
		$\kappa = 0.5$	linear $q(\cdot)$	-0.002 (0.012)	0.532 (0.008)	0.507 (<0.001)	93.3 (0.6)
		$\kappa = 0.5$	non-linear $q(\cdot)$	-0.011 (0.012)	0.520 (0.008)	0.506 (<0.001)	94.5 (0.5)
		$\kappa = 1$	linear $q(\cdot)$	0.008 (0.007)	0.326 (0.005)	0.316 (<0.001)	94.3 (0.5)
		$\kappa = 1$	non-linear $q(\cdot)$	0.007 (0.007)	0.331 (0.005)	0.326 (<0.001)	94.4 (0.5)
STC	$d_{AB(AC)}$	$\kappa = 0$	linear $q(\cdot)$	-0.004 (0.030)	1.356 (0.021)	1.313 (0.001)	94.3 (0.5)
		$\kappa = 0$	non-linear $q(\cdot)$	0.303 (0.029)	1.283 (0.020)	1.262 (0.001)	93.8 (0.5)
		$\kappa = 0.5$	linear $q(\cdot)$	-0.015 (0.011)	0.474 (0.007)	0.457 (<0.001)	94.1 (0.5)
		$\kappa = 0.5$	non-linear $q(\cdot)$	0.012 (0.010)	0.451 (0.007)	0.444 (<0.001)	94.6 (0.5)
		$\kappa = 1$	linear $q(\cdot)$	-0.019 (0.005)	0.241 (0.004)	0.234 (<0.001)	94.1 (0.5)
		$\kappa = 1$	non-linear $q(\cdot)$	-0.006 (0.005)	0.229 (0.004)	0.227 (<0.001)	95.0 (0.5)
	$d_{BC(AC)}$	$\kappa = 0$	linear $q(\cdot)$	-0.010 (0.031)	1.375 (0.022)	1.330 (0.001)	94.3 (0.5)
		$\kappa = 0$	non-linear $q(\cdot)$	-0.300 (0.029)	1.315 (0.021)	1.289 (0.001)	93.5 (0.6)
		$\kappa = 0.5$	linear $q(\cdot)$	0.002 (0.012)	0.521 (0.008)	0.502 (<0.001)	93.8 (0.5)
		$\kappa = 0.5$	non-linear $q(\cdot)$	-0.019 (0.012)	0.515 (0.008)	0.505 (<0.001)	94.8 (0.5)
		$\kappa = 1$	linear $q(\cdot)$	0.005 (0.007)	0.321 (0.005)	0.313 (<0.001)	94.8 (0.5)
		$\kappa = 1$	non-linear $q(\cdot)$	-0.002 (0.007)	0.329 (0.005)	0.325 (<0.001)	94.5 (0.5)
MAIC	$d_{AB(AC)}$	$\kappa = 0.5$	linear $q(\cdot)$	-0.184 (0.023)	1.046 (0.017)	- (-)	88.2 (0.7)
		$\kappa = 0.5$	non-linear $q(\cdot)$	-0.028 (0.022)	0.998 (0.016)	- (-)	92.6 (0.6)
		$\kappa = 1$	linear $q(\cdot)$	-0.005 (0.006)	0.254 (0.004)	0.253 (<0.001)	94.7 (0.5)
		$\kappa = 1$	non-linear $q(\cdot)$	-0.006 (0.005)	0.240 (0.004)	0.243 (<0.001)	95.5 (0.5)
	$d_{BC(AC)}$	$\kappa = 0.5$	linear $q(\cdot)$	0.170 (0.024)	1.064 (0.017)	- (-)	94.8 (0.5)
		$\kappa = 0.5$	non-linear $q(\cdot)$	0.021 (0.023)	1.028 (0.016)	- (-)	93.5 (0.6)
		$\kappa = 1$	linear $q(\cdot)$	-0.009 (0.007)	0.330 (0.005)	0.328 (<0.001)	95.4 (0.5)
		$\kappa = 1$	non-linear $q(\cdot)$	-0.002 (0.008)	0.337 (0.005)	0.337 (<0.001)	94.8 (0.5)
Bucher	$d_{AB(AC)}$	$\kappa = 0$	linear $q(\cdot)$	-0.984 (0.005)	0.204 (0.003)	0.202 (<0.001)	0.1 (<0.1)
		$\kappa = 0$	non-linear $q(\cdot)$	-0.670 (0.004)	0.191 (0.003)	0.193 (<0.001)	6.3 (0.5)
		$\kappa = 0.5$	linear $q(\cdot)$	-0.309 (0.005)	0.204 (0.003)	0.202 (<0.001)	67.9 (1.0)
		$\kappa = 0.5$	non-linear $q(\cdot)$	-0.295 (0.004)	0.191 (0.003)	0.193 (<0.001)	66.8 (1.1)
		$\kappa = 1$	linear $q(\cdot)$	-0.084 (0.005)	0.204 (0.003)	0.202 (<0.001)	93.0 (0.6)
		$\kappa = 1$	non-linear $q(\cdot)$	-0.091 (0.004)	0.191 (0.003)	0.193 (<0.001)	92.8 (0.6)
	$d_{BC(AC)}$	$\kappa = 0$	linear $q(\cdot)$	0.971 (0.007)	0.292 (0.005)	0.290 (<0.001)	8.7 (0.6)
		$\kappa = 0$	non-linear $q(\cdot)$	0.673 (0.007)	0.326 (0.005)	0.325 (<0.001)	45.6 (1.1)
		$\kappa = 0.5$	linear $q(\cdot)$	0.296 (0.007)	0.292 (0.005)	0.290 (<0.001)	81.9 (0.9)
		$\kappa = 0.5$	non-linear $q(\cdot)$	0.288 (0.007)	0.310 (0.005)	0.309 (<0.001)	85.0 (0.8)
		$\kappa = 1$	linear $q(\cdot)$	0.071 (0.007)	0.292 (0.005)	0.290 (<0.001)	94.5 (0.5)
		$\kappa = 1$	non-linear $q(\cdot)$	0.083 (0.007)	0.303 (0.005)	0.302 (<0.001)	94.2 (0.5)

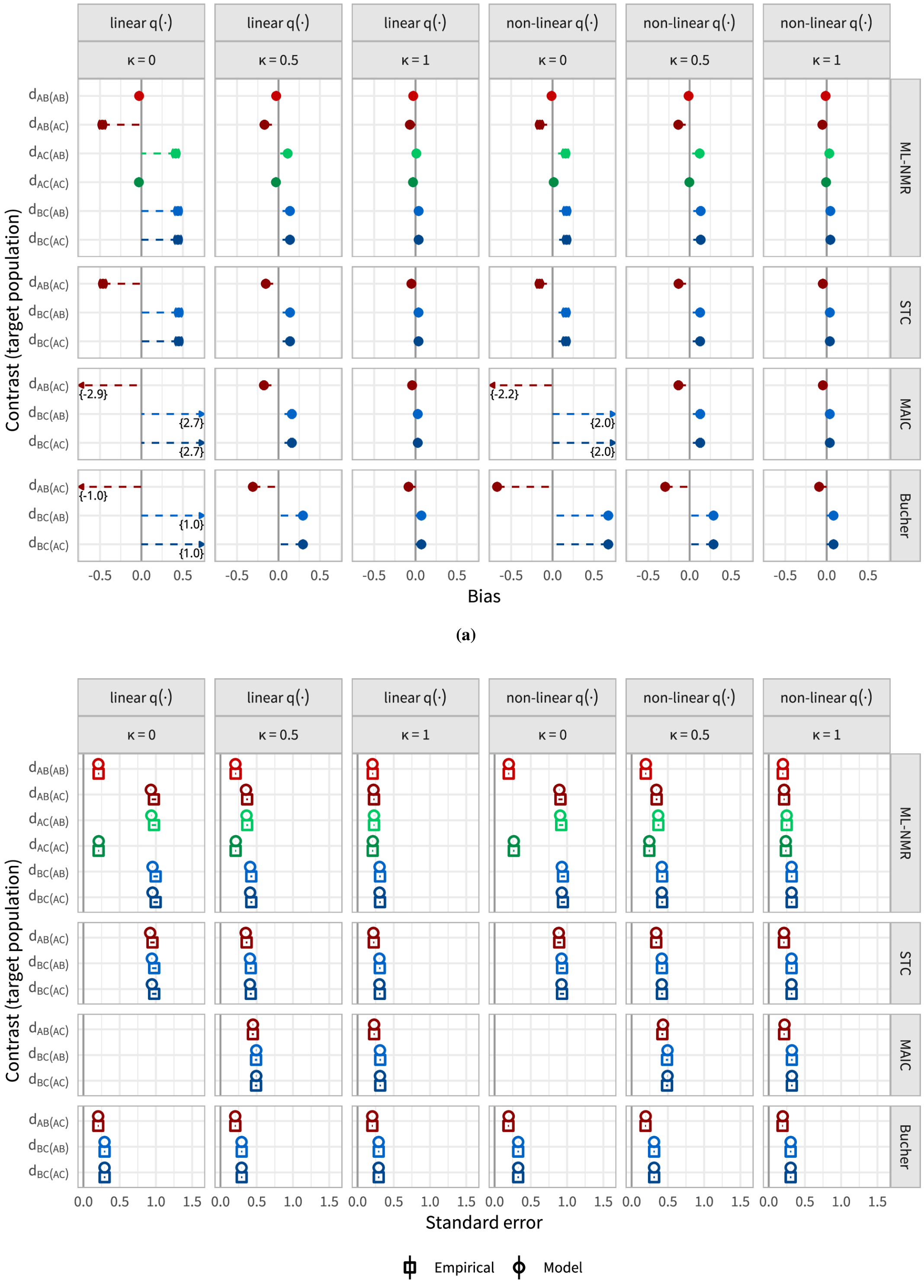

(a)

(b)

FIGURE B14 Bias (a) and standard errors (b) for the population-average contrast estimates for scenarios e and f, along with 95% Monte Carlo confidence intervals. One of the two effect modifiers was not adjusted for. The between-study overlap and covariate-outcome relationship are varied jointly. The points are coloured by contrast, with lighter shades for the AB population and darker for the AC population.

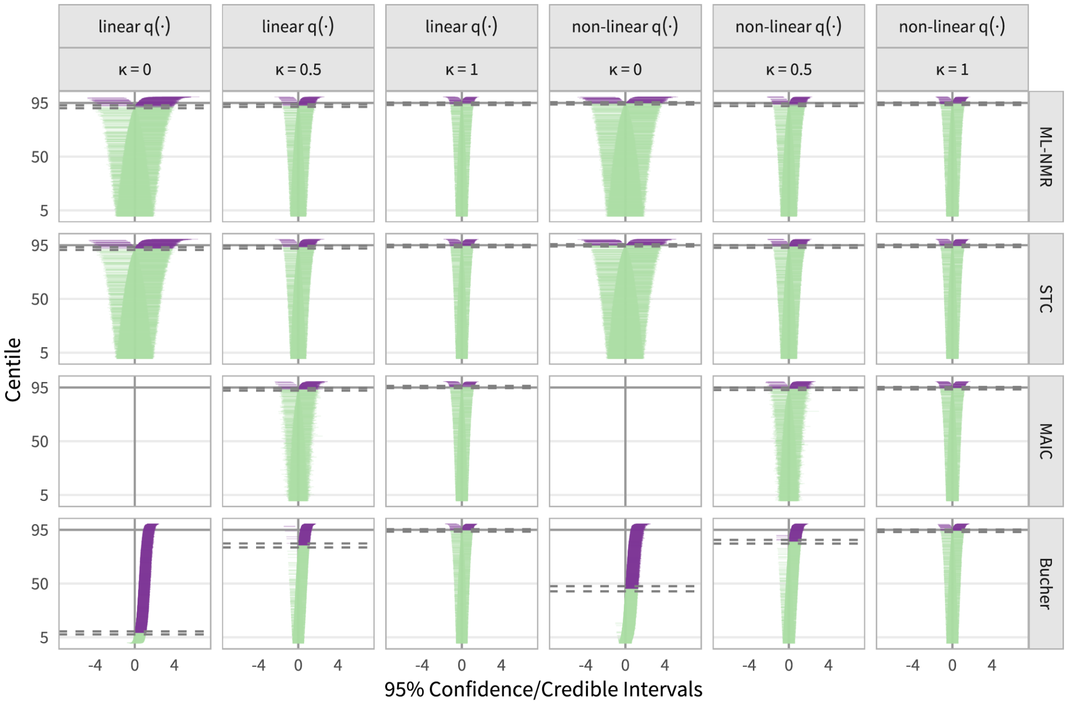

FIGURE B15 Coverage zip plots for the $d_{BC(AC)}$ contrast estimate for scenarios e and f. One of the two effect modifiers was not adjusted for. The between-study overlap and covariate-outcome relationship are varied jointly. The 95% confidence/credible intervals are coloured as coverers (green) or non-coverers (purple), and the colour change should occur at the 95th centile (i.e. nominal coverage). The horizontal dashed lines are 95% Monte Carlo confidence intervals for the coverage.

TABLE B10 Simulation results for scenarios e and f, only adjusting for one of two effect modifiers. The between-study overlap and covariate-outcome relationship are varied jointly. Monte Carlo standard errors for each statistic are shown in brackets.

Method	Contrast	Scenario		Bias	Empirical SE	Model SE	Coverage
ML-NMR	$d_{AB(AB)}$	$\kappa = 0$	linear $q(\cdot)$	-0.027 (0.005)	0.209 (0.003)	0.205 (<0.001)	93.6 (0.5)
		$\kappa = 0$	non-linear $q(\cdot)$	-0.012 (0.004)	0.195 (0.003)	0.195 (<0.001)	95.1 (0.5)
		$\kappa = 0.5$	linear $q(\cdot)$	-0.027 (0.005)	0.209 (0.003)	0.205 (<0.001)	93.8 (0.5)
		$\kappa = 0.5$	non-linear $q(\cdot)$	-0.012 (0.004)	0.195 (0.003)	0.195 (<0.001)	94.7 (0.5)
		$\kappa = 1$	linear $q(\cdot)$	-0.028 (0.005)	0.209 (0.003)	0.205 (<0.001)	93.8 (0.5)
		$\kappa = 1$	non-linear $q(\cdot)$	-0.013 (0.004)	0.195 (0.003)	0.195 (<0.001)	94.7 (0.5)
	$d_{AC(AB)}$	$\kappa = 0$	linear $q(\cdot)$	0.414 (0.022)	0.973 (0.015)	0.936 (<0.001)	91.7 (0.6)
		$\kappa = 0$	non-linear $q(\cdot)$	0.157 (0.021)	0.919 (0.015)	0.904 (<0.001)	94.0 (0.5)
		$\kappa = 0.5$	linear $q(\cdot)$	0.111 (0.008)	0.368 (0.006)	0.358 (<0.001)	92.9 (0.6)
		$\kappa = 0.5$	non-linear $q(\cdot)$	0.121 (0.008)	0.369 (0.006)	0.365 (<0.001)	93.2 (0.6)
		$\kappa = 1$	linear $q(\cdot)$	0.010 (0.005)	0.227 (0.004)	0.226 (<0.001)	94.8 (0.5)
		$\kappa = 1$	non-linear $q(\cdot)$	0.032 (0.006)	0.248 (0.004)	0.247 (<0.001)	94.5 (0.5)
	$d_{BC(AB)}$	$\kappa = 0$	linear $q(\cdot)$	0.441 (0.022)	0.997 (0.016)	0.953 (<0.001)	91.8 (0.6)
		$\kappa = 0$	non-linear $q(\cdot)$	0.169 (0.021)	0.943 (0.015)	0.927 (<0.001)	94.6 (0.5)
		$\kappa = 0.5$	linear $q(\cdot)$	0.139 (0.010)	0.425 (0.007)	0.408 (<0.001)	92.8 (0.6)
		$\kappa = 0.5$	non-linear $q(\cdot)$	0.134 (0.009)	0.421 (0.007)	0.415 (<0.001)	93.4 (0.6)
		$\kappa = 1$	linear $q(\cdot)$	0.038 (0.007)	0.310 (0.005)	0.303 (<0.001)	94.3 (0.5)
		$\kappa = 1$	non-linear $q(\cdot)$	0.045 (0.007)	0.318 (0.005)	0.315 (<0.001)	94.2 (0.5)
	$d_{AB(AC)}$	$\kappa = 0$	linear $q(\cdot)$	-0.472 (0.022)	0.972 (0.015)	0.929 (<0.001)	90.8 (0.6)
		$\kappa = 0$	non-linear $q(\cdot)$	-0.155 (0.020)	0.909 (0.014)	0.893 (<0.001)	94.5 (0.5)
		$\kappa = 0.5$	linear $q(\cdot)$	-0.169 (0.008)	0.367 (0.006)	0.351 (<0.001)	91.3 (0.6)
		$\kappa = 0.5$	non-linear $q(\cdot)$	-0.137 (0.008)	0.345 (0.005)	0.340 (<0.001)	93.0 (0.6)
		$\kappa = 1$	linear $q(\cdot)$	-0.068 (0.005)	0.227 (0.004)	0.219 (<0.001)	93.2 (0.6)
		$\kappa = 1$	non-linear $q(\cdot)$	-0.052 (0.005)	0.212 (0.003)	0.212 (<0.001)	94.4 (0.5)
	$d_{AC(AC)}$	$\kappa = 0$	linear $q(\cdot)$	-0.030 (0.005)	0.209 (0.003)	0.210 (<0.001)	95.1 (0.5)
		$\kappa = 0$	non-linear $q(\cdot)$	0.014 (0.006)	0.265 (0.004)	0.264 (<0.001)	94.8 (0.5)
		$\kappa = 0.5$	linear $q(\cdot)$	-0.030 (0.005)	0.209 (0.003)	0.210 (<0.001)	95.0 (0.5)
		$\kappa = 0.5$	non-linear $q(\cdot)$	-0.003 (0.005)	0.244 (0.004)	0.243 (<0.001)	95.0 (0.5)
		$\kappa = 1$	linear $q(\cdot)$	-0.030 (0.005)	0.209 (0.003)	0.210 (<0.001)	95.0 (0.5)
		$\kappa = 1$	non-linear $q(\cdot)$	-0.007 (0.005)	0.237 (0.004)	0.235 (<0.001)	95.2 (0.5)
	$d_{BC(AC)}$	$\kappa = 0$	linear $q(\cdot)$	0.441 (0.022)	0.997 (0.016)	0.953 (<0.001)	91.8 (0.6)
		$\kappa = 0$	non-linear $q(\cdot)$	0.169 (0.021)	0.943 (0.015)	0.927 (<0.001)	94.6 (0.5)
		$\kappa = 0.5$	linear $q(\cdot)$	0.139 (0.010)	0.425 (0.007)	0.408 (<0.001)	92.8 (0.6)
		$\kappa = 0.5$	non-linear $q(\cdot)$	0.134 (0.009)	0.421 (0.007)	0.415 (<0.001)	93.4 (0.6)
		$\kappa = 1$	linear $q(\cdot)$	0.038 (0.007)	0.310 (0.005)	0.303 (<0.001)	94.3 (0.5)
		$\kappa = 1$	non-linear $q(\cdot)$	0.045 (0.007)	0.318 (0.005)	0.315 (<0.001)	94.2 (0.5)
STC	$d_{AB(AC)}$	$\kappa = 0$	linear $q(\cdot)$	-0.464 (0.021)	0.952 (0.015)	0.920 (<0.001)	91.7 (0.6)
		$\kappa = 0$	non-linear $q(\cdot)$	-0.158 (0.020)	0.891 (0.014)	0.885 (<0.001)	94.8 (0.5)
		$\kappa = 0.5$	linear $q(\cdot)$	-0.153 (0.008)	0.361 (0.006)	0.348 (<0.001)	92.4 (0.6)
		$\kappa = 0.5$	non-linear $q(\cdot)$	-0.134 (0.008)	0.339 (0.005)	0.338 (<0.001)	93.7 (0.5)
		$\kappa = 1$	linear $q(\cdot)$	-0.050 (0.005)	0.223 (0.004)	0.218 (<0.001)	93.7 (0.5)
		$\kappa = 1$	non-linear $q(\cdot)$	-0.046 (0.005)	0.210 (0.003)	0.210 (<0.001)	94.8 (0.5)
	$d_{BC(AC)}$	$\kappa = 0$	linear $q(\cdot)$	0.451 (0.022)	0.976 (0.015)	0.943 (<0.001)	92.2 (0.6)
		$\kappa = 0$	non-linear $q(\cdot)$	0.161 (0.021)	0.930 (0.015)	0.923 (<0.001)	94.9 (0.5)
		$\kappa = 0.5$	linear $q(\cdot)$	0.140 (0.009)	0.418 (0.007)	0.405 (<0.001)	93.3 (0.6)
		$\kappa = 0.5$	non-linear $q(\cdot)$	0.127 (0.009)	0.418 (0.007)	0.415 (<0.001)	93.8 (0.5)
		$\kappa = 1$	linear $q(\cdot)$	0.036 (0.007)	0.307 (0.005)	0.301 (<0.001)	94.3 (0.5)
		$\kappa = 1$	non-linear $q(\cdot)$	0.038 (0.007)	0.316 (0.005)	0.314 (<0.001)	94.4 (0.5)
MAIC	$d_{AB(AC)}$	$\kappa = 0$	linear $q(\cdot)$	-2.902 (0.087)	3.913 (0.062)	- (-)	44.4 (1.1)
		$\kappa = 0$	non-linear $q(\cdot)$	-2.239 (0.084)	3.770 (0.060)	- (-)	22.2 (0.9)
		$\kappa = 0.5$	linear $q(\cdot)$	-0.175 (0.010)	0.447 (0.007)	0.447 (0.001)	91.0 (0.6)
		$\kappa = 0.5$	non-linear $q(\cdot)$	-0.135 (0.009)	0.423 (0.007)	0.432 (0.001)	91.7 (0.6)
		$\kappa = 1$	linear $q(\cdot)$	-0.041 (0.005)	0.229 (0.004)	0.228 (<0.001)	93.6 (0.5)
		$\kappa = 1$	non-linear $q(\cdot)$	-0.046 (0.005)	0.213 (0.003)	0.218 (<0.001)	94.7 (0.5)
	$d_{BC(AC)}$	$\kappa = 0$	linear $q(\cdot)$	2.709 (0.087)	3.872 (0.061)	- (-)	- (-)
		$\kappa = 0$	non-linear $q(\cdot)$	2.044 (0.081)	3.643 (0.058)	- (-)	- (-)
		$\kappa = 0.5$	linear $q(\cdot)$	0.162 (0.011)	0.490 (0.008)	0.493 (0.001)	93.5 (0.6)
		$\kappa = 0.5$	non-linear $q(\cdot)$	0.128 (0.011)	0.488 (0.008)	0.495 (0.001)	93.9 (0.5)
		$\kappa = 1$	linear $q(\cdot)$	0.027 (0.007)	0.313 (0.005)	0.309 (<0.001)	95.5 (0.5)
		$\kappa = 1$	non-linear $q(\cdot)$	0.038 (0.007)	0.319 (0.005)	0.319 (<0.001)	94.5 (0.5)
Bucher	$d_{AB(AC)}$	$\kappa = 0$	linear $q(\cdot)$	-0.984 (0.005)	0.204 (0.003)	0.202 (<0.001)	0.1 (<0.1)
		$\kappa = 0$	non-linear $q(\cdot)$	-0.670 (0.004)	0.191 (0.003)	0.193 (<0.001)	6.3 (0.5)

TABLE B10 (continued)

Method	Contrast	Scenario		Bias	Empirical SE	Model SE	Coverage
		$\kappa = 0.5$	linear $q(\cdot)$	-0.309 (0.005)	0.204 (0.003)	0.202 (<0.001)	67.9 (1.0)
		$\kappa = 0.5$	non-linear $q(\cdot)$	-0.295 (0.004)	0.191 (0.003)	0.193 (<0.001)	66.8 (1.1)
		$\kappa = 1$	linear $q(\cdot)$	-0.084 (0.005)	0.204 (0.003)	0.202 (<0.001)	93.0 (0.6)
		$\kappa = 1$	non-linear $q(\cdot)$	-0.091 (0.004)	0.191 (0.003)	0.193 (<0.001)	92.8 (0.6)
	$d_{BC(AC)}$	$\kappa = 0$	linear $q(\cdot)$	0.971 (0.007)	0.292 (0.005)	0.290 (<0.001)	8.7 (0.6)
		$\kappa = 0$	non-linear $q(\cdot)$	0.673 (0.007)	0.326 (0.005)	0.325 (<0.001)	45.6 (1.1)
		$\kappa = 0.5$	linear $q(\cdot)$	0.296 (0.007)	0.292 (0.005)	0.290 (<0.001)	81.9 (0.9)
		$\kappa = 0.5$	non-linear $q(\cdot)$	0.288 (0.007)	0.310 (0.005)	0.309 (<0.001)	85.0 (0.8)
		$\kappa = 1$	linear $q(\cdot)$	0.071 (0.007)	0.292 (0.005)	0.290 (<0.001)	94.5 (0.5)
		$\kappa = 1$	non-linear $q(\cdot)$	0.083 (0.007)	0.303 (0.005)	0.302 (<0.001)	94.2 (0.5)

B.6 Scenarios g, h, and i

TABLE B11 Simulation results for scenarios g, h, and i, adjusting for all effect modifiers. The covariate distributions and correlation structures in each study are varied jointly. Monte Carlo standard errors for each statistic are shown in brackets.

Method	Contrast	Scenario			Bias	Empirical SE	Model SE	Coverage
ML-NMR	$d_{AB(AB)}$	$X_{(AB)} \sim$ Gam	$X_{(AC)} \sim$ Gam	$\rho_{(AB)} = 0.25, \rho_{(AC)} = 0$	-0.058 (0.005)	0.207 (0.003)	0.207 (<0.001)	94.0 (0.5)
		$X_{(AB)} \sim$ Gam	$X_{(AC)} \sim$ Gam	$\rho_{(AB)} = \rho_{(AC)} = 0.25$	-0.058 (0.005)	0.207 (0.003)	0.207 (<0.001)	94.2 (0.5)
		$X_{(AB)} \sim$ Gam	$X_{(AC)} \sim$ Gam	$\rho_{(AB)} = 0.25, \rho_{(AC)} = 0.5$	-0.058 (0.005)	0.207 (0.003)	0.207 (<0.001)	94.4 (0.5)
		$X_{(AB)} \sim$ Gam	$X_{(AC)} \sim$ N	$\rho_{(AB)} = 0.25, \rho_{(AC)} = 0$	-0.058 (0.005)	0.207 (0.003)	0.207 (<0.001)	94.2 (0.5)
		$X_{(AB)} \sim$ Gam	$X_{(AC)} \sim$ N	$\rho_{(AB)} = \rho_{(AC)} = 0.25$	-0.058 (0.005)	0.207 (0.003)	0.207 (<0.001)	94.2 (0.5)
		$X_{(AB)} \sim$ Gam	$X_{(AC)} \sim$ N	$\rho_{(AB)} = 0.25, \rho_{(AC)} = 0.5$	-0.058 (0.005)	0.207 (0.003)	0.207 (<0.001)	94.1 (0.5)
		$X_{(AB)} \sim$ N	$X_{(AC)} \sim$ Gam	$\rho_{(AB)} = 0.25, \rho_{(AC)} = 0$	-0.059 (0.005)	0.208 (0.003)	0.207 (<0.001)	94.5 (0.5)
		$X_{(AB)} \sim$ N	$X_{(AC)} \sim$ Gam	$\rho_{(AB)} = \rho_{(AC)} = 0.25$	-0.059 (0.005)	0.208 (0.003)	0.207 (<0.001)	94.3 (0.5)
		$X_{(AB)} \sim$ N	$X_{(AC)} \sim$ Gam	$\rho_{(AB)} = 0.25, \rho_{(AC)} = 0.5$	-0.059 (0.005)	0.208 (0.003)	0.207 (<0.001)	94.5 (0.5)
		$X_{(AB)} \sim$ N	$X_{(AC)} \sim$ N	$\rho_{(AB)} = 0.25, \rho_{(AC)} = 0$	-0.059 (0.005)	0.208 (0.003)	0.207 (<0.001)	94.2 (0.5)
		$X_{(AB)} \sim$ N	$X_{(AC)} \sim$ N	$\rho_{(AB)} = \rho_{(AC)} = 0.25$	-0.059 (0.005)	0.208 (0.003)	0.207 (<0.001)	94.3 (0.5)
		$X_{(AB)} \sim$ N	$X_{(AC)} \sim$ N	$\rho_{(AB)} = 0.25, \rho_{(AC)} = 0.5$	-0.059 (0.005)	0.208 (0.003)	0.207 (<0.001)	94.4 (0.5)
	$d_{AC(AB)}$	$X_{(AB)} \sim$ Gam	$X_{(AC)} \sim$ Gam	$\rho_{(AB)} = 0.25, \rho_{(AC)} = 0$	-0.028 (0.011)	0.476 (0.008)	0.470 (<0.001)	94.8 (0.5)
		$X_{(AB)} \sim$ Gam	$X_{(AC)} \sim$ Gam	$\rho_{(AB)} = \rho_{(AC)} = 0.25$	-0.028 (0.011)	0.477 (0.008)	0.470 (<0.001)	95.0 (0.5)
		$X_{(AB)} \sim$ Gam	$X_{(AC)} \sim$ Gam	$\rho_{(AB)} = 0.25, \rho_{(AC)} = 0.5$	-0.028 (0.011)	0.476 (0.008)	0.470 (<0.001)	94.8 (0.5)
		$X_{(AB)} \sim$ Gam	$X_{(AC)} \sim$ N	$\rho_{(AB)} = 0.25, \rho_{(AC)} = 0$	-0.028 (0.011)	0.477 (0.008)	0.470 (<0.001)	95.0 (0.5)
		$X_{(AB)} \sim$ Gam	$X_{(AC)} \sim$ N	$\rho_{(AB)} = \rho_{(AC)} = 0.25$	-0.028 (0.011)	0.477 (0.008)	0.470 (<0.001)	94.9 (0.5)
		$X_{(AB)} \sim$ Gam	$X_{(AC)} \sim$ N	$\rho_{(AB)} = 0.25, \rho_{(AC)} = 0.5$	-0.028 (0.011)	0.477 (0.008)	0.470 (<0.001)	94.8 (0.5)
		$X_{(AB)} \sim$ N	$X_{(AC)} \sim$ Gam	$\rho_{(AB)} = 0.25, \rho_{(AC)} = 0$	-0.051 (0.011)	0.478 (0.008)	0.471 (<0.001)	94.9 (0.5)
		$X_{(AB)} \sim$ N	$X_{(AC)} \sim$ Gam	$\rho_{(AB)} = \rho_{(AC)} = 0.25$	-0.051 (0.011)	0.478 (0.008)	0.472 (<0.001)	94.8 (0.5)
		$X_{(AB)} \sim$ N	$X_{(AC)} \sim$ Gam	$\rho_{(AB)} = 0.25, \rho_{(AC)} = 0.5$	-0.051 (0.011)	0.478 (0.008)	0.471 (<0.001)	94.6 (0.5)
		$X_{(AB)} \sim$ N	$X_{(AC)} \sim$ N	$\rho_{(AB)} = 0.25, \rho_{(AC)} = 0$	-0.051 (0.011)	0.478 (0.008)	0.472 (<0.001)	94.9 (0.5)
		$X_{(AB)} \sim$ N	$X_{(AC)} \sim$ N	$\rho_{(AB)} = \rho_{(AC)} = 0.25$	-0.051 (0.011)	0.478 (0.008)	0.472 (<0.001)	94.7 (0.5)
		$X_{(AB)} \sim$ N	$X_{(AC)} \sim$ N	$\rho_{(AB)} = 0.25, \rho_{(AC)} = 0.5$	-0.051 (0.011)	0.478 (0.008)	0.471 (<0.001)	94.8 (0.5)
	$d_{BC(AB)}$	$X_{(AB)} \sim$ Gam	$X_{(AC)} \sim$ Gam	$\rho_{(AB)} = 0.25, \rho_{(AC)} = 0$	0.030 (0.012)	0.516 (0.008)	0.509 (<0.001)	94.5 (0.5)
		$X_{(AB)} \sim$ Gam	$X_{(AC)} \sim$ Gam	$\rho_{(AB)} = \rho_{(AC)} = 0.25$	0.030 (0.012)	0.516 (0.008)	0.509 (<0.001)	94.6 (0.5)
		$X_{(AB)} \sim$ Gam	$X_{(AC)} \sim$ Gam	$\rho_{(AB)} = 0.25, \rho_{(AC)} = 0.5$	0.030 (0.012)	0.516 (0.008)	0.509 (<0.001)	94.7 (0.5)
		$X_{(AB)} \sim$ Gam	$X_{(AC)} \sim$ N	$\rho_{(AB)} = 0.25, \rho_{(AC)} = 0$	0.030 (0.012)	0.516 (0.008)	0.509 (<0.001)	94.7 (0.5)
		$X_{(AB)} \sim$ Gam	$X_{(AC)} \sim$ N	$\rho_{(AB)} = \rho_{(AC)} = 0.25$	0.029 (0.012)	0.516 (0.008)	0.509 (<0.001)	94.8 (0.5)
		$X_{(AB)} \sim$ Gam	$X_{(AC)} \sim$ N	$\rho_{(AB)} = 0.25, \rho_{(AC)} = 0.5$	0.030 (0.012)	0.516 (0.008)	0.509 (<0.001)	94.8 (0.5)
		$X_{(AB)} \sim$ N	$X_{(AC)} \sim$ Gam	$\rho_{(AB)} = 0.25, \rho_{(AC)} = 0$	0.008 (0.012)	0.514 (0.008)	0.508 (<0.001)	94.8 (0.5)
		$X_{(AB)} \sim$ N	$X_{(AC)} \sim$ Gam	$\rho_{(AB)} = \rho_{(AC)} = 0.25$	0.008 (0.012)	0.515 (0.008)	0.508 (<0.001)	94.7 (0.5)
		$X_{(AB)} \sim$ N	$X_{(AC)} \sim$ Gam	$\rho_{(AB)} = 0.25, \rho_{(AC)} = 0.5$	0.008 (0.012)	0.514 (0.008)	0.508 (<0.001)	94.5 (0.5)
		$X_{(AB)} \sim$ N	$X_{(AC)} \sim$ N	$\rho_{(AB)} = 0.25, \rho_{(AC)} = 0$	0.008 (0.012)	0.514 (0.008)	0.509 (<0.001)	94.8 (0.5)
		$X_{(AB)} \sim$ N	$X_{(AC)} \sim$ N	$\rho_{(AB)} = \rho_{(AC)} = 0.25$	0.007 (0.012)	0.515 (0.008)	0.509 (<0.001)	94.8 (0.5)
		$X_{(AB)} \sim$ N	$X_{(AC)} \sim$ N	$\rho_{(AB)} = 0.25, \rho_{(AC)} = 0.5$	0.008 (0.012)	0.514 (0.008)	0.508 (<0.001)	94.5 (0.5)
	$d_{AB(AC)}$	$X_{(AB)} \sim$ Gam	$X_{(AC)} \sim$ Gam	$\rho_{(AB)} = 0.25, \rho_{(AC)} = 0$	-0.069 (0.011)	0.470 (0.007)	0.464 (<0.001)	94.2 (0.5)
		$X_{(AB)} \sim$ Gam	$X_{(AC)} \sim$ Gam	$\rho_{(AB)} = \rho_{(AC)} = 0.25$	-0.069 (0.011)	0.471 (0.007)	0.464 (<0.001)	94.2 (0.5)
		$X_{(AB)} \sim$ Gam	$X_{(AC)} \sim$ Gam	$\rho_{(AB)} = 0.25, \rho_{(AC)} = 0.5$	-0.069 (0.011)	0.470 (0.007)	0.464 (<0.001)	94.4 (0.5)
		$X_{(AB)} \sim$ Gam	$X_{(AC)} \sim$ N	$\rho_{(AB)} = 0.25, \rho_{(AC)} = 0$	-0.069 (0.011)	0.471 (0.007)	0.465 (<0.001)	94.2 (0.5)
		$X_{(AB)} \sim$ Gam	$X_{(AC)} \sim$ N	$\rho_{(AB)} = \rho_{(AC)} = 0.25$	-0.069 (0.011)	0.470 (0.007)	0.464 (<0.001)	94.2 (0.5)
		$X_{(AB)} \sim$ Gam	$X_{(AC)} \sim$ N	$\rho_{(AB)} = 0.25, \rho_{(AC)} = 0.5$	-0.069 (0.011)	0.470 (0.007)	0.464 (<0.001)	94.2 (0.5)
		$X_{(AB)} \sim$ N	$X_{(AC)} \sim$ Gam	$\rho_{(AB)} = 0.25, \rho_{(AC)} = 0$	-0.047 (0.010)	0.468 (0.007)	0.463 (<0.001)	94.4 (0.5)
		$X_{(AB)} \sim$ N	$X_{(AC)} \sim$ Gam	$\rho_{(AB)} = \rho_{(AC)} = 0.25$	-0.047 (0.010)	0.468 (0.007)	0.463 (<0.001)	94.4 (0.5)
		$X_{(AB)} \sim$ N	$X_{(AC)} \sim$ Gam	$\rho_{(AB)} = 0.25, \rho_{(AC)} = 0.5$	-0.047 (0.010)	0.468 (0.007)	0.463 (<0.001)	94.5 (0.5)
		$X_{(AB)} \sim$ N	$X_{(AC)} \sim$ N	$\rho_{(AB)} = 0.25, \rho_{(AC)} = 0$	-0.047 (0.010)	0.468 (0.007)	0.463 (<0.001)	94.3 (0.5)
		$X_{(AB)} \sim$ N	$X_{(AC)} \sim$ N	$\rho_{(AB)} = \rho_{(AC)} = 0.25$	-0.047 (0.010)	0.468 (0.007)	0.463 (<0.001)	94.4 (0.5)
		$X_{(AB)} \sim$ N	$X_{(AC)} \sim$ N	$\rho_{(AB)} = 0.25, \rho_{(AC)} = 0.5$	-0.047 (0.010)	0.468 (0.007)	0.463 (<0.001)	94.3 (0.5)
	$d_{AC(AC)}$	$X_{(AB)} \sim$ Gam	$X_{(AC)} \sim$ Gam	$\rho_{(AB)} = 0.25, \rho_{(AC)} = 0$	-0.039 (0.005)	0.219 (0.003)	0.211 (<0.001)	94.0 (0.5)
		$X_{(AB)} \sim$ Gam	$X_{(AC)} \sim$ Gam	$\rho_{(AB)} = \rho_{(AC)} = 0.25$	-0.040 (0.005)	0.220 (0.003)	0.211 (<0.001)	94.0 (0.5)
		$X_{(AB)} \sim$ Gam	$X_{(AC)} \sim$ Gam	$\rho_{(AB)} = 0.25, \rho_{(AC)} = 0.5$	-0.039 (0.005)	0.220 (0.003)	0.211 (<0.001)	94.0 (0.5)
		$X_{(AB)} \sim$ Gam	$X_{(AC)} \sim$ N	$\rho_{(AB)} = 0.25, \rho_{(AC)} = 0$	-0.039 (0.005)	0.220 (0.003)	0.211 (<0.001)	94.3 (0.5)
		$X_{(AB)} \sim$ Gam	$X_{(AC)} \sim$ N	$\rho_{(AB)} = \rho_{(AC)} = 0.25$	-0.040 (0.005)	0.220 (0.003)	0.211 (<0.001)	93.9 (0.5)
		$X_{(AB)} \sim$ Gam	$X_{(AC)} \sim$ N	$\rho_{(AB)} = 0.25, \rho_{(AC)} = 0.5$	-0.039 (0.005)	0.220 (0.003)	0.211 (<0.001)	93.8 (0.5)
		$X_{(AB)} \sim$ N	$X_{(AC)} \sim$ Gam	$\rho_{(AB)} = 0.25, \rho_{(AC)} = 0$	-0.039 (0.005)	0.219 (0.003)	0.211 (<0.001)	94.1 (0.5)
		$X_{(AB)} \sim$ N	$X_{(AC)} \sim$ Gam	$\rho_{(AB)} = \rho_{(AC)} = 0.25$	-0.039 (0.005)	0.220 (0.003)	0.211 (<0.001)	94.0 (0.5)
		$X_{(AB)} \sim$ N	$X_{(AC)} \sim$ Gam	$\rho_{(AB)} = 0.25, \rho_{(AC)} = 0.5$	-0.039 (0.005)	0.220 (0.003)	0.211 (<0.001)	94.2 (0.5)
		$X_{(AB)} \sim$ N	$X_{(AC)} \sim$ N	$\rho_{(AB)} = 0.25, \rho_{(AC)} = 0$	-0.039 (0.005)	0.220 (0.003)	0.211 (<0.001)	94.2 (0.5)
		$X_{(AB)} \sim$ N	$X_{(AC)} \sim$ N	$\rho_{(AB)} = \rho_{(AC)} = 0.25$	-0.040 (0.005)	0.220 (0.003)	0.211 (<0.001)	94.0 (0.5)
		$X_{(AB)} \sim$ N	$X_{(AC)} \sim$ N	$\rho_{(AB)} = 0.25, \rho_{(AC)} = 0.5$	-0.039 (0.005)	0.220 (0.003)	0.211 (<0.001)	93.8 (0.5)
	$d_{BC(AC)}$	$X_{(AB)} \sim$ Gam	$X_{(AC)} \sim$ Gam	$\rho_{(AB)} = 0.25, \rho_{(AC)} = 0$	0.030 (0.012)	0.516 (0.008)	0.509 (<0.001)	94.5 (0.5)
		$X_{(AB)} \sim$ Gam	$X_{(AC)} \sim$ Gam	$\rho_{(AB)} = \rho_{(AC)} = 0.25$	0.030 (0.012)	0.516 (0.008)	0.509 (<0.001)	94.6 (0.5)

TABLE B11 (continued)

Method	Contrast	Scenario			Bias	Empirical SE	Model SE	Coverage
		$X_{(AB)} \sim$ Gam	$X_{(AC)} \sim$ Gam	$\rho_{(AB)} = 0.25, \rho_{(AC)} = 0.5$	0.030 (0.012)	0.516 (0.008)	0.509 (<0.001)	94.7 (0.5)
		$X_{(AB)} \sim$ Gam	$X_{(AC)} \sim$ N	$\rho_{(AB)} = 0.25, \rho_{(AC)} = 0$	0.030 (0.012)	0.516 (0.008)	0.509 (<0.001)	94.7 (0.5)
		$X_{(AB)} \sim$ Gam	$X_{(AC)} \sim$ N	$\rho_{(AB)} = \rho_{(AC)} = 0.25$	0.029 (0.012)	0.516 (0.008)	0.509 (<0.001)	94.8 (0.5)
		$X_{(AB)} \sim$ Gam	$X_{(AC)} \sim$ N	$\rho_{(AB)} = 0.25, \rho_{(AC)} = 0.5$	0.030 (0.012)	0.516 (0.008)	0.509 (<0.001)	94.8 (0.5)
		$X_{(AB)} \sim$ N	$X_{(AC)} \sim$ Gam	$\rho_{(AB)} = 0.25, \rho_{(AC)} = 0$	0.008 (0.012)	0.514 (0.008)	0.508 (<0.001)	94.8 (0.5)
		$X_{(AB)} \sim$ N	$X_{(AC)} \sim$ Gam	$\rho_{(AB)} = \rho_{(AC)} = 0.25$	0.008 (0.012)	0.515 (0.008)	0.508 (<0.001)	94.7 (0.5)
		$X_{(AB)} \sim$ N	$X_{(AC)} \sim$ Gam	$\rho_{(AB)} = 0.25, \rho_{(AC)} = 0.5$	0.008 (0.012)	0.514 (0.008)	0.508 (<0.001)	94.5 (0.5)
		$X_{(AB)} \sim$ N	$X_{(AC)} \sim$ N	$\rho_{(AB)} = 0.25, \rho_{(AC)} = 0$	0.008 (0.012)	0.514 (0.008)	0.509 (<0.001)	94.8 (0.5)
		$X_{(AB)} \sim$ N	$X_{(AC)} \sim$ N	$\rho_{(AB)} = \rho_{(AC)} = 0.25$	0.007 (0.012)	0.515 (0.008)	0.509 (<0.001)	94.8 (0.5)
		$X_{(AB)} \sim$ N	$X_{(AC)} \sim$ N	$\rho_{(AB)} = 0.25, \rho_{(AC)} = 0.5$	0.008 (0.012)	0.514 (0.008)	0.508 (<0.001)	94.5 (0.5)
STC	$d_{AB(AC)}$	$X_{(AB)} \sim$ Gam	$X_{(AC)} \sim$ Gam	$\rho_{(AB)} = 0.25, \rho_{(AC)} = 0$	-0.038 (0.010)	0.458 (0.007)	0.458 (<0.001)	95.2 (0.5)
		$X_{(AB)} \sim$ Gam	$X_{(AC)} \sim$ Gam	$\rho_{(AB)} = \rho_{(AC)} = 0.25$	-0.037 (0.010)	0.458 (0.007)	0.458 (<0.001)	95.2 (0.5)
		$X_{(AB)} \sim$ Gam	$X_{(AC)} \sim$ Gam	$\rho_{(AB)} = 0.25, \rho_{(AC)} = 0.5$	-0.037 (0.010)	0.457 (0.007)	0.458 (<0.001)	95.2 (0.5)
		$X_{(AB)} \sim$ Gam	$X_{(AC)} \sim$ N	$\rho_{(AB)} = 0.25, \rho_{(AC)} = 0$	-0.038 (0.010)	0.458 (0.007)	0.458 (<0.001)	95.2 (0.5)
		$X_{(AB)} \sim$ Gam	$X_{(AC)} \sim$ N	$\rho_{(AB)} = \rho_{(AC)} = 0.25$	-0.037 (0.010)	0.458 (0.007)	0.458 (<0.001)	95.2 (0.5)
		$X_{(AB)} \sim$ Gam	$X_{(AC)} \sim$ N	$\rho_{(AB)} = 0.25, \rho_{(AC)} = 0.5$	-0.037 (0.010)	0.457 (0.007)	0.458 (<0.001)	95.2 (0.5)
		$X_{(AB)} \sim$ N	$X_{(AC)} \sim$ Gam	$\rho_{(AB)} = 0.25, \rho_{(AC)} = 0$	-0.026 (0.010)	0.457 (0.007)	0.458 (<0.001)	95.1 (0.5)
		$X_{(AB)} \sim$ N	$X_{(AC)} \sim$ Gam	$\rho_{(AB)} = \rho_{(AC)} = 0.25$	-0.026 (0.010)	0.457 (0.007)	0.458 (<0.001)	95.1 (0.5)
		$X_{(AB)} \sim$ N	$X_{(AC)} \sim$ Gam	$\rho_{(AB)} = 0.25, \rho_{(AC)} = 0.5$	-0.026 (0.010)	0.457 (0.007)	0.458 (<0.001)	95.2 (0.5)
		$X_{(AB)} \sim$ N	$X_{(AC)} \sim$ N	$\rho_{(AB)} = 0.25, \rho_{(AC)} = 0$	-0.026 (0.010)	0.457 (0.007)	0.458 (<0.001)	95.1 (0.5)
		$X_{(AB)} \sim$ N	$X_{(AC)} \sim$ N	$\rho_{(AB)} = \rho_{(AC)} = 0.25$	-0.026 (0.010)	0.457 (0.007)	0.458 (<0.001)	95.1 (0.5)
		$X_{(AB)} \sim$ N	$X_{(AC)} \sim$ N	$\rho_{(AB)} = 0.25, \rho_{(AC)} = 0.5$	-0.026 (0.010)	0.457 (0.007)	0.458 (<0.001)	95.2 (0.5)
	$d_{BC(AC)}$	$X_{(AB)} \sim$ Gam	$X_{(AC)} \sim$ Gam	$\rho_{(AB)} = 0.25, \rho_{(AC)} = 0$	0.023 (0.011)	0.504 (0.008)	0.503 (<0.001)	95.2 (0.5)
		$X_{(AB)} \sim$ Gam	$X_{(AC)} \sim$ Gam	$\rho_{(AB)} = \rho_{(AC)} = 0.25$	0.022 (0.011)	0.504 (0.008)	0.503 (<0.001)	95.2 (0.5)
		$X_{(AB)} \sim$ Gam	$X_{(AC)} \sim$ Gam	$\rho_{(AB)} = 0.25, \rho_{(AC)} = 0.5$	0.022 (0.011)	0.504 (0.008)	0.503 (<0.001)	95.3 (0.5)
		$X_{(AB)} \sim$ Gam	$X_{(AC)} \sim$ N	$\rho_{(AB)} = 0.25, \rho_{(AC)} = 0$	0.023 (0.011)	0.504 (0.008)	0.503 (<0.001)	95.2 (0.5)
		$X_{(AB)} \sim$ Gam	$X_{(AC)} \sim$ N	$\rho_{(AB)} = \rho_{(AC)} = 0.25$	0.022 (0.011)	0.504 (0.008)	0.503 (<0.001)	95.2 (0.5)
		$X_{(AB)} \sim$ Gam	$X_{(AC)} \sim$ N	$\rho_{(AB)} = 0.25, \rho_{(AC)} = 0.5$	0.023 (0.011)	0.504 (0.008)	0.503 (<0.001)	95.3 (0.5)
		$X_{(AB)} \sim$ N	$X_{(AC)} \sim$ Gam	$\rho_{(AB)} = 0.25, \rho_{(AC)} = 0$	0.011 (0.011)	0.503 (0.008)	0.503 (<0.001)	95.0 (0.5)
		$X_{(AB)} \sim$ N	$X_{(AC)} \sim$ Gam	$\rho_{(AB)} = \rho_{(AC)} = 0.25$	0.011 (0.011)	0.503 (0.008)	0.503 (<0.001)	95.0 (0.5)
		$X_{(AB)} \sim$ N	$X_{(AC)} \sim$ Gam	$\rho_{(AB)} = 0.25, \rho_{(AC)} = 0.5$	0.011 (0.011)	0.503 (0.008)	0.503 (<0.001)	94.9 (0.5)
		$X_{(AB)} \sim$ N	$X_{(AC)} \sim$ N	$\rho_{(AB)} = 0.25, \rho_{(AC)} = 0$	0.012 (0.011)	0.503 (0.008)	0.503 (<0.001)	95.0 (0.5)
		$X_{(AB)} \sim$ N	$X_{(AC)} \sim$ N	$\rho_{(AB)} = \rho_{(AC)} = 0.25$	0.011 (0.011)	0.503 (0.008)	0.503 (<0.001)	95.0 (0.5)
		$X_{(AB)} \sim$ N	$X_{(AC)} \sim$ N	$\rho_{(AB)} = 0.25, \rho_{(AC)} = 0.5$	0.011 (0.011)	0.503 (0.008)	0.503 (<0.001)	94.9 (0.5)
MAIC	$d_{AB(AC)}$	$X_{(AB)} \sim$ Gam	$X_{(AC)} \sim$ Gam	$\rho_{(AB)} = 0.25, \rho_{(AC)} = 0$	-0.138 (0.019)	0.850 (0.013)	- (-)	90.1 (0.7)
		$X_{(AB)} \sim$ Gam	$X_{(AC)} \sim$ Gam	$\rho_{(AB)} = \rho_{(AC)} = 0.25$	-0.138 (0.019)	0.849 (0.013)	- (-)	90.2 (0.7)
		$X_{(AB)} \sim$ Gam	$X_{(AC)} \sim$ Gam	$\rho_{(AB)} = 0.25, \rho_{(AC)} = 0.5$	-0.139 (0.019)	0.849 (0.013)	- (-)	90.2 (0.7)
		$X_{(AB)} \sim$ Gam	$X_{(AC)} \sim$ N	$\rho_{(AB)} = 0.25, \rho_{(AC)} = 0$	-0.138 (0.019)	0.850 (0.013)	10.701 (4.733)	90.2 (0.7)
		$X_{(AB)} \sim$ Gam	$X_{(AC)} \sim$ N	$\rho_{(AB)} = \rho_{(AC)} = 0.25$	-0.139 (0.019)	0.850 (0.013)	49.602 (22.145)	90.2 (0.7)
		$X_{(AB)} \sim$ Gam	$X_{(AC)} \sim$ N	$\rho_{(AB)} = 0.25, \rho_{(AC)} = 0.5$	-0.139 (0.019)	0.849 (0.013)	- (-)	90.2 (0.7)
		$X_{(AB)} \sim$ N	$X_{(AC)} \sim$ Gam	$\rho_{(AB)} = 0.25, \rho_{(AC)} = 0$	-0.219 (0.024)	1.091 (0.017)	- (-)	89.4 (0.7)
		$X_{(AB)} \sim$ N	$X_{(AC)} \sim$ Gam	$\rho_{(AB)} = \rho_{(AC)} = 0.25$	-0.221 (0.025)	1.112 (0.018)	- (-)	89.5 (0.7)
		$X_{(AB)} \sim$ N	$X_{(AC)} \sim$ Gam	$\rho_{(AB)} = 0.25, \rho_{(AC)} = 0.5$	-0.220 (0.024)	1.089 (0.017)	- (-)	89.5 (0.7)
		$X_{(AB)} \sim$ N	$X_{(AC)} \sim$ N	$\rho_{(AB)} = 0.25, \rho_{(AC)} = 0$	-0.219 (0.024)	1.083 (0.017)	- (-)	89.4 (0.7)
		$X_{(AB)} \sim$ N	$X_{(AC)} \sim$ N	$\rho_{(AB)} = \rho_{(AC)} = 0.25$	-0.219 (0.024)	1.087 (0.017)	- (-)	89.5 (0.7)
		$X_{(AB)} \sim$ N	$X_{(AC)} \sim$ N	$\rho_{(AB)} = 0.25, \rho_{(AC)} = 0.5$	-0.220 (0.025)	1.097 (0.017)	- (-)	89.5 (0.7)
	$d_{BC(AC)}$	$X_{(AB)} \sim$ Gam	$X_{(AC)} \sim$ Gam	$\rho_{(AB)} = 0.25, \rho_{(AC)} = 0$	0.123 (0.020)	0.875 (0.014)	- (-)	95.4 (0.5)
		$X_{(AB)} \sim$ Gam	$X_{(AC)} \sim$ Gam	$\rho_{(AB)} = \rho_{(AC)} = 0.25$	0.123 (0.020)	0.875 (0.014)	- (-)	95.1 (0.5)
		$X_{(AB)} \sim$ Gam	$X_{(AC)} \sim$ Gam	$\rho_{(AB)} = 0.25, \rho_{(AC)} = 0.5$	0.124 (0.020)	0.875 (0.014)	- (-)	95.1 (0.5)
		$X_{(AB)} \sim$ Gam	$X_{(AC)} \sim$ N	$\rho_{(AB)} = 0.25, \rho_{(AC)} = 0$	0.123 (0.020)	0.875 (0.014)	10.703 (4.732)	95.1 (0.5)
		$X_{(AB)} \sim$ Gam	$X_{(AC)} \sim$ N	$\rho_{(AB)} = \rho_{(AC)} = 0.25$	0.123 (0.020)	0.875 (0.014)	49.603 (22.145)	95.0 (0.5)
		$X_{(AB)} \sim$ Gam	$X_{(AC)} \sim$ N	$\rho_{(AB)} = 0.25, \rho_{(AC)} = 0.5$	0.124 (0.020)	0.875 (0.014)	- (-)	95.0 (0.5)
		$X_{(AB)} \sim$ N	$X_{(AC)} \sim$ Gam	$\rho_{(AB)} = 0.25, \rho_{(AC)} = 0$	0.205 (0.025)	1.106 (0.017)	- (-)	96.0 (0.4)
		$X_{(AB)} \sim$ N	$X_{(AC)} \sim$ Gam	$\rho_{(AB)} = \rho_{(AC)} = 0.25$	0.206 (0.025)	1.126 (0.018)	- (-)	95.8 (0.5)
		$X_{(AB)} \sim$ N	$X_{(AC)} \sim$ Gam	$\rho_{(AB)} = 0.25, \rho_{(AC)} = 0.5$	0.204 (0.025)	1.104 (0.017)	- (-)	95.6 (0.5)
		$X_{(AB)} \sim$ N	$X_{(AC)} \sim$ N	$\rho_{(AB)} = 0.25, \rho_{(AC)} = 0$	0.204 (0.025)	1.098 (0.017)	- (-)	95.8 (0.4)
		$X_{(AB)} \sim$ N	$X_{(AC)} \sim$ N	$\rho_{(AB)} = \rho_{(AC)} = 0.25$	0.204 (0.025)	1.103 (0.017)	- (-)	95.9 (0.4)
		$X_{(AB)} \sim$ N	$X_{(AC)} \sim$ N	$\rho_{(AB)} = 0.25, \rho_{(AC)} = 0.5$	0.206 (0.025)	1.112 (0.018)	- (-)	95.4 (0.5)
Bucher	$d_{AB(AC)}$	$X_{(AB)} \sim$ Gam	$X_{(AC)} \sim$ Gam	$\rho_{(AB)} = 0.25, \rho_{(AC)} = 0$	-0.318 (0.004)	0.199 (0.003)	0.202 (<0.001)	65.5 (1.1)
		$X_{(AB)} \sim$ Gam	$X_{(AC)} \sim$ Gam	$\rho_{(AB)} = \rho_{(AC)} = 0.25$	-0.318 (0.004)	0.199 (0.003)	0.202 (<0.001)	65.5 (1.1)
		$X_{(AB)} \sim$ Gam	$X_{(AC)} \sim$ Gam	$\rho_{(AB)} = 0.25, \rho_{(AC)} = 0.5$	-0.318 (0.004)	0.199 (0.003)	0.202 (<0.001)	65.5 (1.1)
		$X_{(AB)} \sim$ Gam	$X_{(AC)} \sim$ N	$\rho_{(AB)} = 0.25, \rho_{(AC)} = 0$	-0.318 (0.004)	0.199 (0.003)	0.202 (<0.001)	65.5 (1.1)
		$X_{(AB)} \sim$ Gam	$X_{(AC)} \sim$ N	$\rho_{(AB)} = \rho_{(AC)} = 0.25$	-0.318 (0.004)	0.199 (0.003)	0.202 (<0.001)	65.5 (1.1)
		$X_{(AB)} \sim$ Gam	$X_{(AC)} \sim$ N	$\rho_{(AB)} = 0.25, \rho_{(AC)} = 0.5$	-0.318 (0.004)	0.199 (0.003)	0.202 (<0.001)	65.5 (1.1)
		$X_{(AB)} \sim$ N	$X_{(AC)} \sim$ Gam	$\rho_{(AB)} = 0.25, \rho_{(AC)} = 0$	-0.318 (0.004)	0.199 (0.003)	0.202 (<0.001)	65.3 (1.1)
		$X_{(AB)} \sim$ N	$X_{(AC)} \sim$ Gam	$\rho_{(AB)} = \rho_{(AC)} = 0.25$	-0.318 (0.004)	0.199 (0.003)	0.202 (<0.001)	65.3 (1.1)

TABLE B11 (continued)

Method	Contrast	Scenario			Bias	Empirical SE	Model SE	Coverage
		$X_{(AB)} \sim$ N	$X_{(AC)} \sim$ Gam	$\rho_{(AB)} = 0.25$, $\rho_{(AC)} = 0.5$	-0.318 (0.004)	0.199 (0.003)	0.202 (<0.001)	65.3 (1.1)
		$X_{(AB)} \sim$ N	$X_{(AC)} \sim$ N	$\rho_{(AB)} = 0.25$, $\rho_{(AC)} = 0$	-0.318 (0.004)	0.199 (0.003)	0.202 (<0.001)	65.3 (1.1)
		$X_{(AB)} \sim$ N	$X_{(AC)} \sim$ N	$\rho_{(AB)} = \rho_{(AC)} = 0.25$	-0.318 (0.004)	0.199 (0.003)	0.202 (<0.001)	65.3 (1.1)
		$X_{(AB)} \sim$ N	$X_{(AC)} \sim$ N	$\rho_{(AB)} = 0.25$, $\rho_{(AC)} = 0.5$	-0.318 (0.004)	0.199 (0.003)	0.202 (<0.001)	65.3 (1.1)
	$d_{BC(AC)}$	$X_{(AB)} \sim$ Gam	$X_{(AC)} \sim$ Gam	$\rho_{(AB)} = 0.25$, $\rho_{(AC)} = 0$	0.303 (0.007)	0.295 (0.005)	0.291 (<0.001)	81.8 (0.9)
		$X_{(AB)} \sim$ Gam	$X_{(AC)} \sim$ Gam	$\rho_{(AB)} = \rho_{(AC)} = 0.25$	0.303 (0.007)	0.296 (0.005)	0.291 (<0.001)	81.8 (0.9)
		$X_{(AB)} \sim$ Gam	$X_{(AC)} \sim$ Gam	$\rho_{(AB)} = 0.25$, $\rho_{(AC)} = 0.5$	0.303 (0.007)	0.296 (0.005)	0.291 (<0.001)	81.7 (0.9)
		$X_{(AB)} \sim$ Gam	$X_{(AC)} \sim$ N	$\rho_{(AB)} = 0.25$, $\rho_{(AC)} = 0$	0.304 (0.007)	0.295 (0.005)	0.291 (<0.001)	81.7 (0.9)
		$X_{(AB)} \sim$ Gam	$X_{(AC)} \sim$ N	$\rho_{(AB)} = \rho_{(AC)} = 0.25$	0.303 (0.007)	0.296 (0.005)	0.291 (<0.001)	81.7 (0.9)
		$X_{(AB)} \sim$ Gam	$X_{(AC)} \sim$ N	$\rho_{(AB)} = 0.25$, $\rho_{(AC)} = 0.5$	0.303 (0.007)	0.295 (0.005)	0.291 (<0.001)	81.6 (0.9)
		$X_{(AB)} \sim$ N	$X_{(AC)} \sim$ Gam	$\rho_{(AB)} = 0.25$, $\rho_{(AC)} = 0$	0.303 (0.007)	0.295 (0.005)	0.291 (<0.001)	81.8 (0.9)
		$X_{(AB)} \sim$ N	$X_{(AC)} \sim$ Gam	$\rho_{(AB)} = \rho_{(AC)} = 0.25$	0.303 (0.007)	0.296 (0.005)	0.291 (<0.001)	81.8 (0.9)
		$X_{(AB)} \sim$ N	$X_{(AC)} \sim$ Gam	$\rho_{(AB)} = 0.25$, $\rho_{(AC)} = 0.5$	0.303 (0.007)	0.296 (0.005)	0.291 (<0.001)	81.8 (0.9)
		$X_{(AB)} \sim$ N	$X_{(AC)} \sim$ N	$\rho_{(AB)} = 0.25$, $\rho_{(AC)} = 0$	0.304 (0.007)	0.295 (0.005)	0.291 (<0.001)	81.8 (0.9)
		$X_{(AB)} \sim$ N	$X_{(AC)} \sim$ N	$\rho_{(AB)} = \rho_{(AC)} = 0.25$	0.303 (0.007)	0.295 (0.005)	0.291 (<0.001)	81.8 (0.9)
		$X_{(AB)} \sim$ N	$X_{(AC)} \sim$ N	$\rho_{(AB)} = 0.25$, $\rho_{(AC)} = 0.5$	0.303 (0.007)	0.295 (0.005)	0.291 (<0.001)	81.8 (0.9)

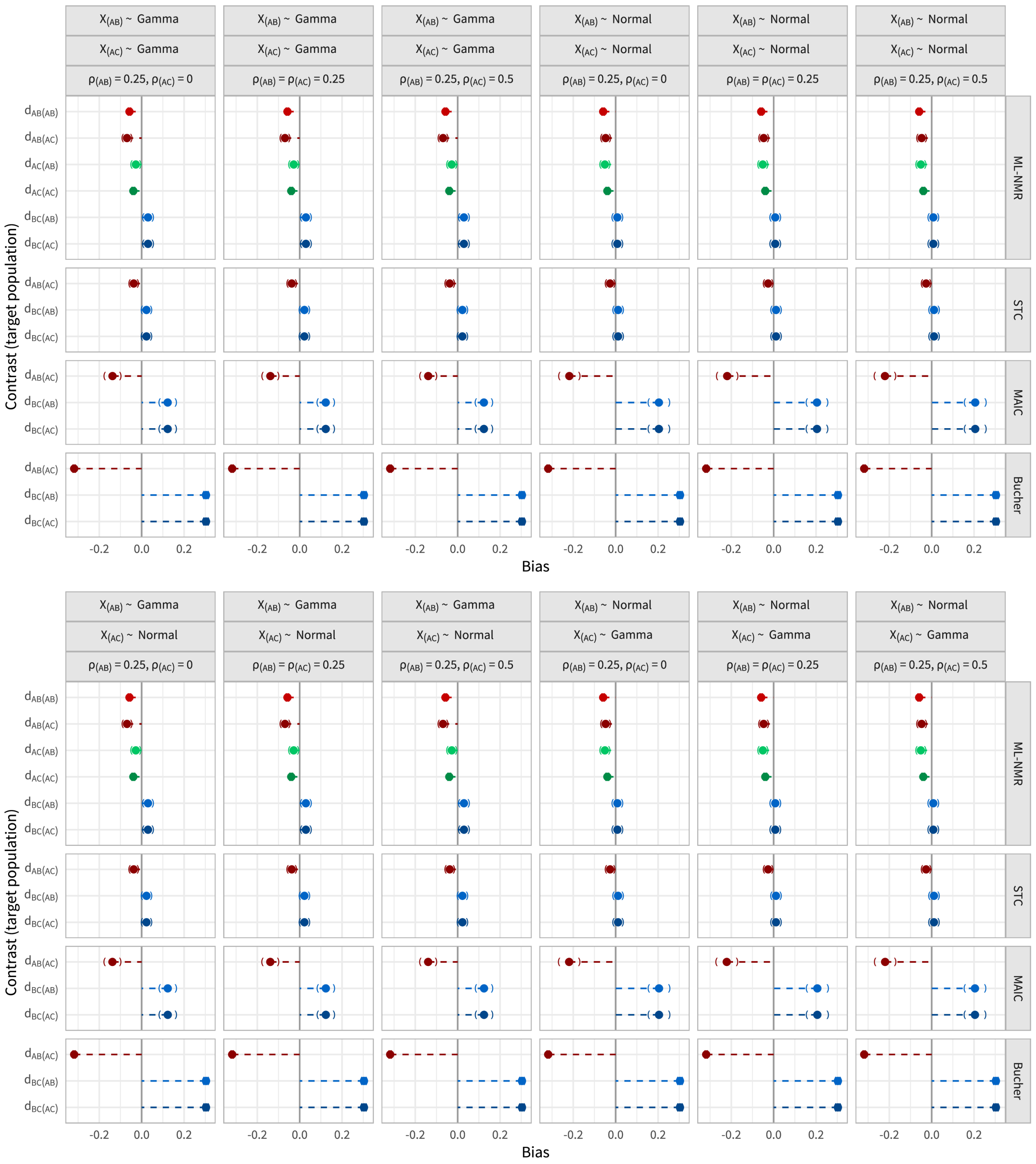

FIGURE B16 Bias in the population-average contrast estimates for scenarios g, h, and i, along with 95% Monte Carlo confidence intervals. Each method (other than Bucher) adjusts for the full set of effect modifiers. The covariate distributions and correlation structures in each study population are varied jointly. The points are coloured by contrast, with lighter shades for the AB population and darker for the AC population.

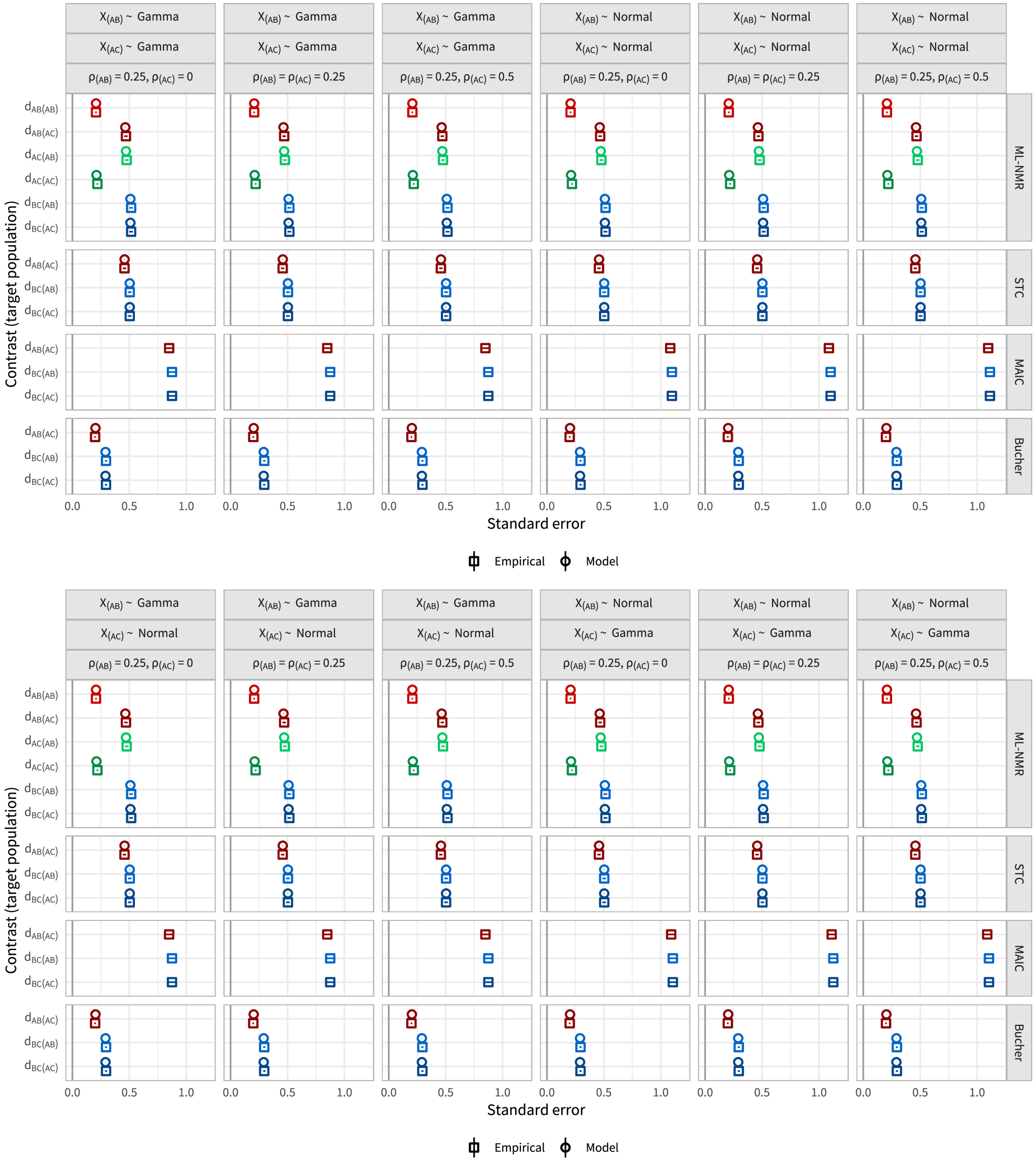

FIGURE B17 Empirical and model standard errors for scenarios g, h, and i, along with 95% Monte Carlo confidence intervals. Each method (other than Bucher) adjusts for the full set of effect modifiers. The covariate distributions and correlation structures in each study population are varied jointly. The points are coloured by contrast, with lighter shades for the AB population and darker for the AC population.

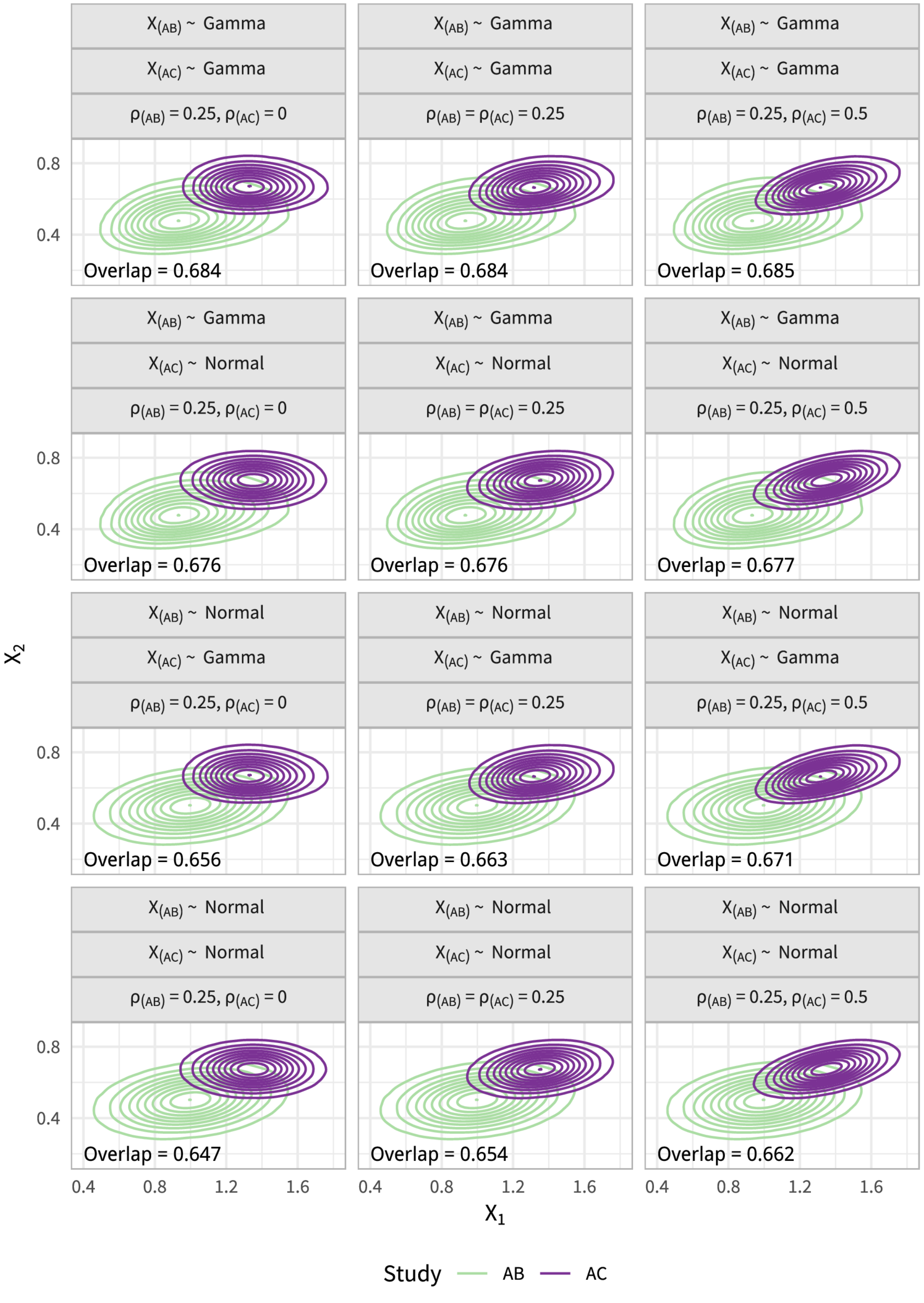

FIGURE B18 Joint covariate distributions in the AB and AC study, as the covariate distributions and correlation between covariates are varied. The true overlap is defined as the proportion of the AC joint density contained within the 95% HDR of the AB joint density, calculated using numerical integration.

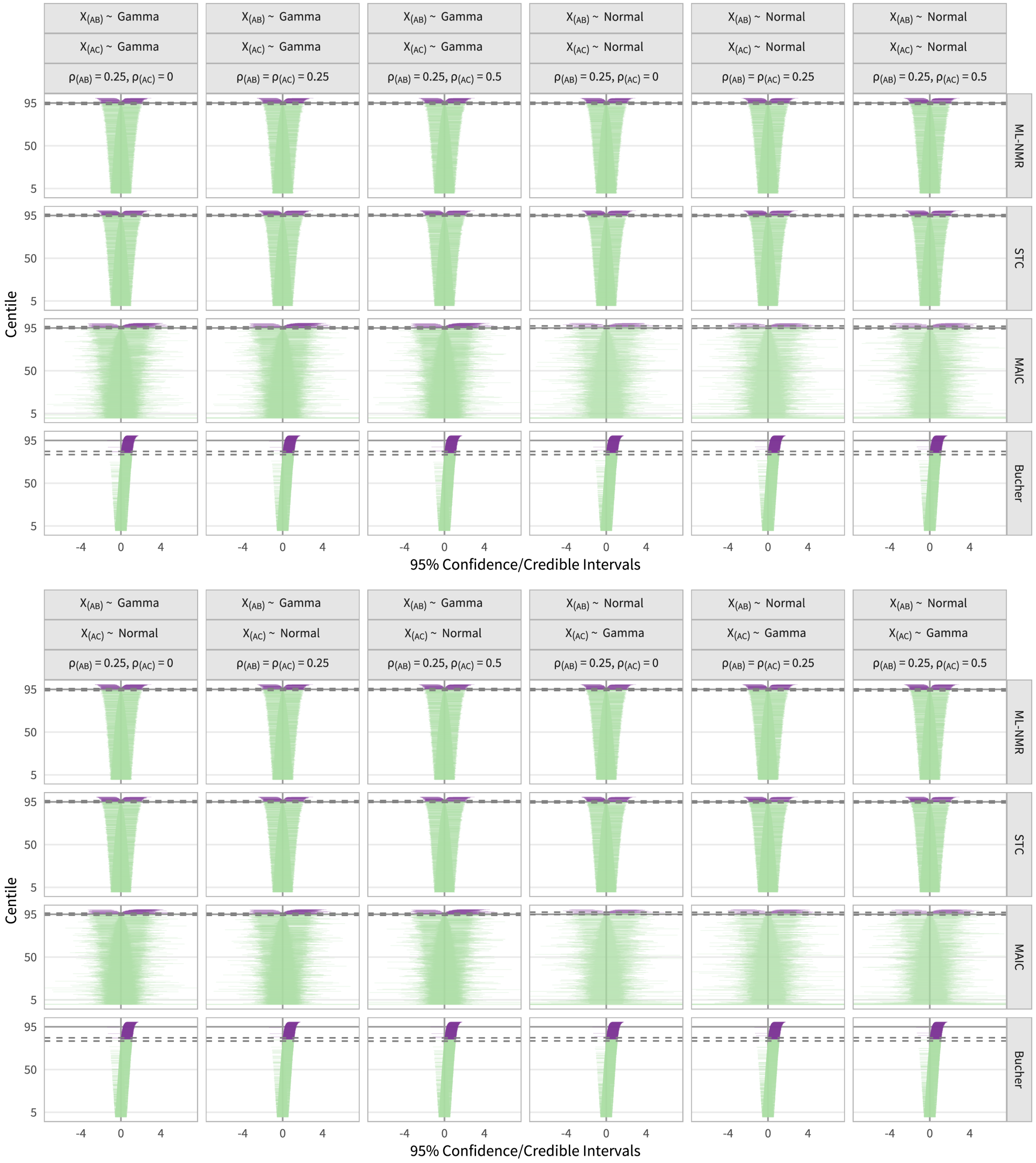

FIGURE B19 Coverage zip plots for the $d_{BC(AC)}$ contrast estimate for scenarios g, h, and i. Each method (other than Bucher) adjusts for the full set of effect modifiers. The covariate distributions and correlation structures in each study population are varied jointly. The 95% confidence/credible intervals are coloured as coverers (green) or non-coverers (purple), and the colour change should occur at the 95th centile (i.e. nominal coverage). The horizontal dashed lines are 95% Monte Carlo confidence intervals for the coverage.

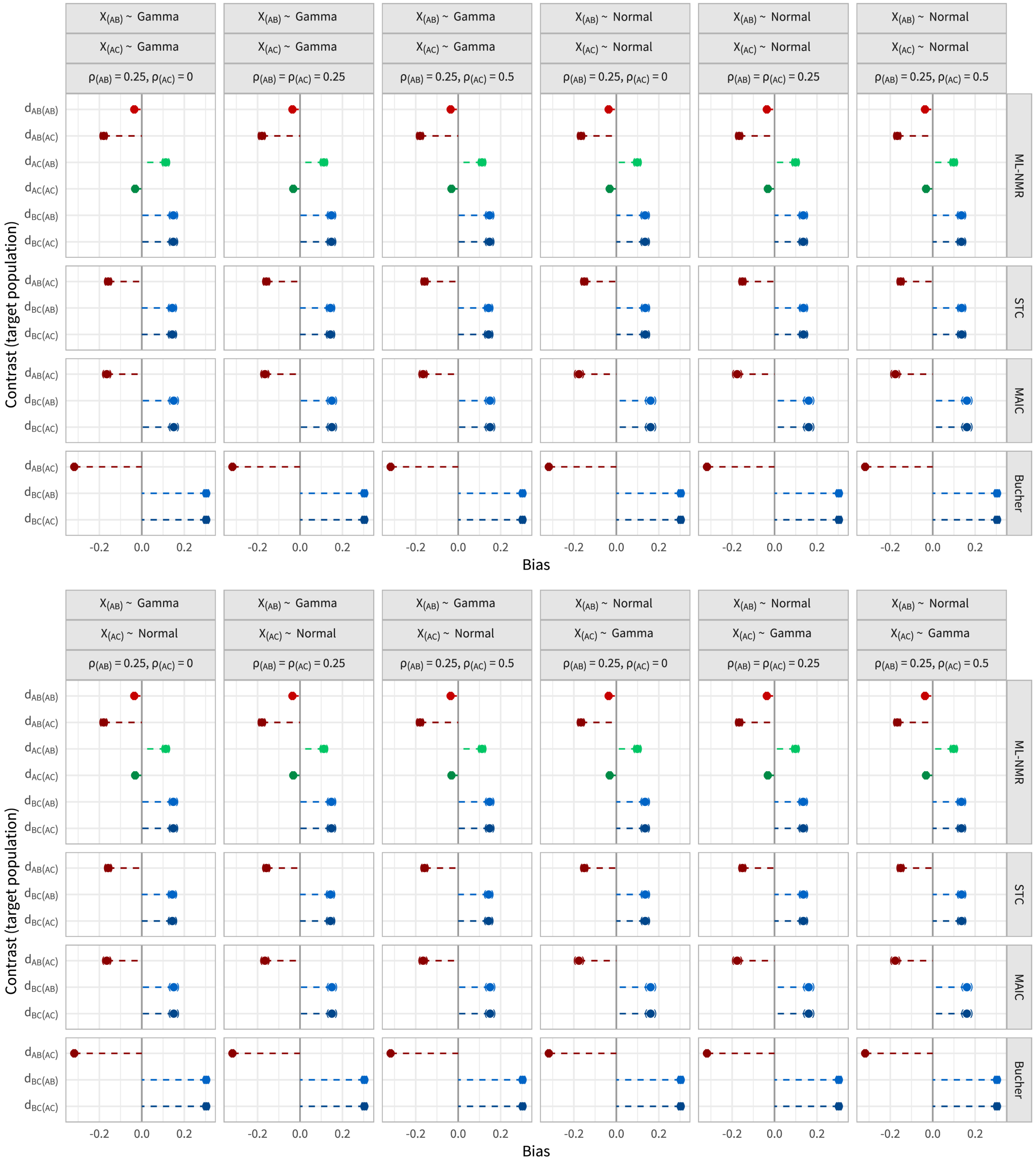

FIGURE B20 Bias in the population-average contrast estimates for scenarios g, h, and i, along with 95% Monte Carlo confidence intervals. One of the two effect modifiers was not adjusted for. The covariate distributions and correlation structures in each study population are varied jointly. The points are coloured by contrast, with lighter shades for the AB population and darker for the AC population.

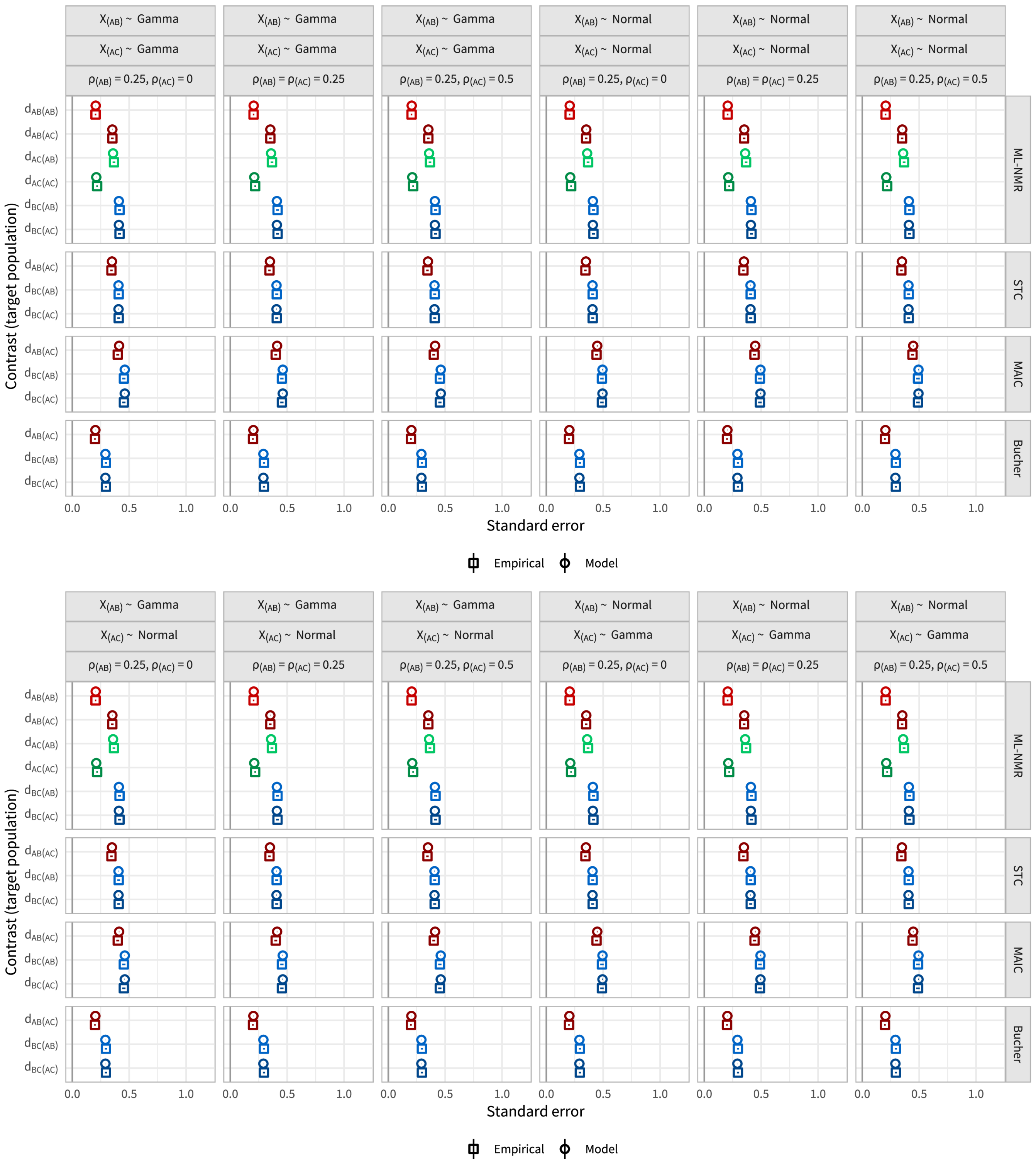

FIGURE B21 Empirical and model standard errors for scenarios g, h, and i, along with 95% Monte Carlo confidence intervals. One of the two effect modifiers was not adjusted for. The covariate distributions and correlation structures in each study population are varied jointly. The points are coloured by contrast, with lighter shades for the AB population and darker for the AC population.

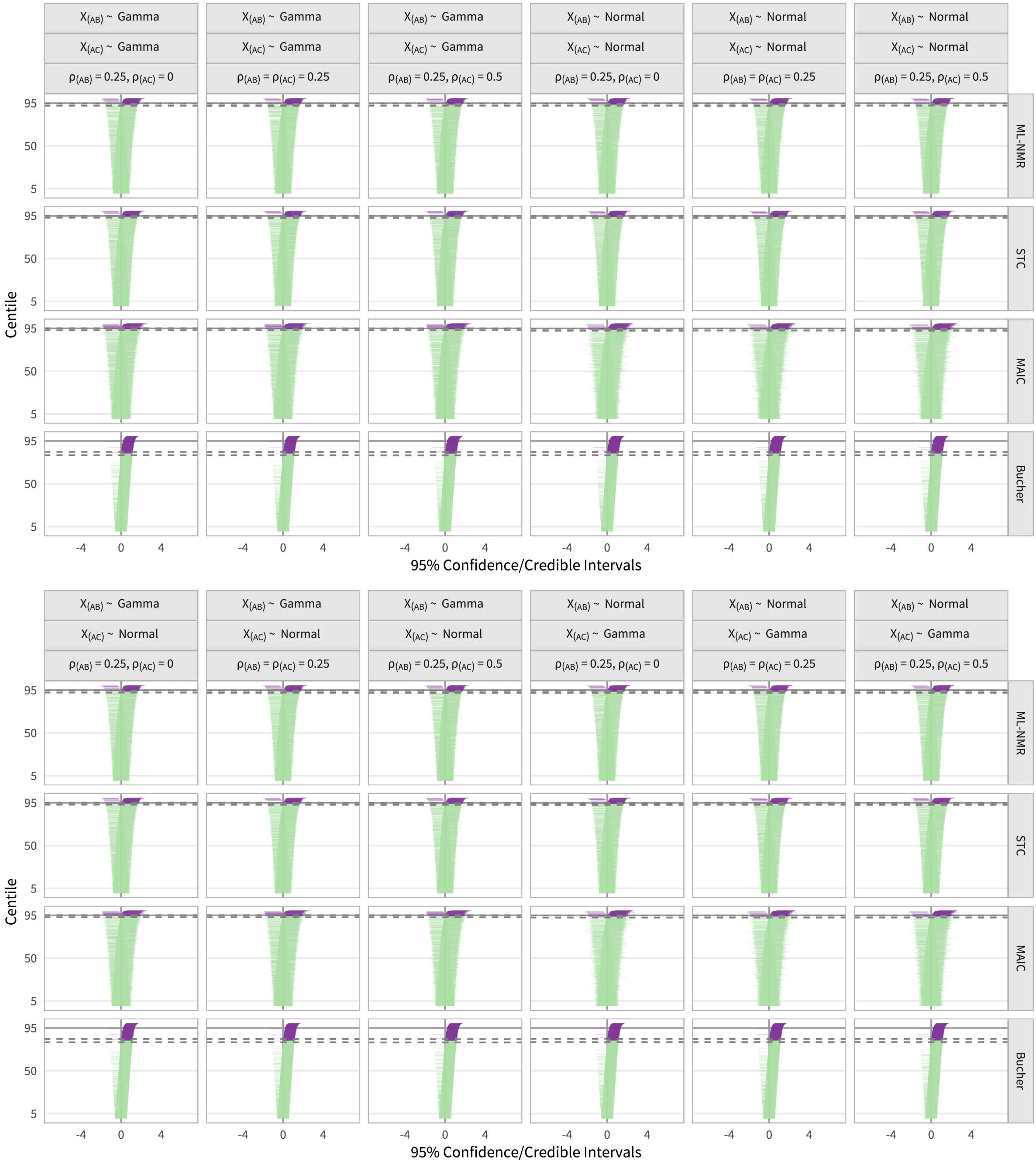

FIGURE B22 Coverage zip plots for the $d_{BC(AC)}$ contrast estimate for scenarios g, h, and i. One of the two effect modifiers was not adjusted for. The covariate distributions and correlation structures in each study population are varied jointly. The 95% confidence/credible intervals are coloured as coverers (green) or non-coverers (purple), and the colour change should occur at the 95th centile (i.e. nominal coverage). The horizontal dashed lines are 95% Monte Carlo confidence intervals for the coverage.

TABLE B12 Simulation results for scenarios g, h, and i, only adjusting for one of two effect modifiers. The covariate distributions and correlation structures in each study are varied jointly. Monte Carlo standard errors for each statistic are shown in brackets.

Method	Contrast	Scenario			Bias	Empirical SE	Model SE	Coverage
ML-NMR	$d_{AB(AB)}$	$X_{(AB)} \sim$ Gam	$X_{(AC)} \sim$ Gam	$\rho_{(AB)} = 0.25, \rho_{(AC)} = 0$	-0.036 (0.005)	0.204 (0.003)	0.205 (<0.001)	95.2 (0.5)
		$X_{(AB)} \sim$ Gam	$X_{(AC)} \sim$ Gam	$\rho_{(AB)} = \rho_{(AC)} = 0.25$	-0.036 (0.005)	0.204 (0.003)	0.205 (<0.001)	95.2 (0.5)
		$X_{(AB)} \sim$ Gam	$X_{(AC)} \sim$ Gam	$\rho_{(AB)} = 0.25, \rho_{(AC)} = 0.5$	-0.036 (0.005)	0.204 (0.003)	0.205 (<0.001)	94.8 (0.5)
		$X_{(AB)} \sim$ Gam	$X_{(AC)} \sim$ N	$\rho_{(AB)} = 0.25, \rho_{(AC)} = 0$	-0.036 (0.005)	0.204 (0.003)	0.205 (<0.001)	95.0 (0.5)
		$X_{(AB)} \sim$ Gam	$X_{(AC)} \sim$ N	$\rho_{(AB)} = \rho_{(AC)} = 0.25$	-0.036 (0.005)	0.204 (0.003)	0.205 (<0.001)	95.0 (0.5)
		$X_{(AB)} \sim$ Gam	$X_{(AC)} \sim$ N	$\rho_{(AB)} = 0.25, \rho_{(AC)} = 0.5$	-0.036 (0.005)	0.204 (0.003)	0.205 (<0.001)	94.8 (0.5)
		$X_{(AB)} \sim$ N	$X_{(AC)} \sim$ Gam	$\rho_{(AB)} = 0.25, \rho_{(AC)} = 0$	-0.036 (0.005)	0.205 (0.003)	0.205 (<0.001)	94.9 (0.5)
		$X_{(AB)} \sim$ N	$X_{(AC)} \sim$ Gam	$\rho_{(AB)} = \rho_{(AC)} = 0.25$	-0.036 (0.005)	0.205 (0.003)	0.205 (<0.001)	94.8 (0.5)
		$X_{(AB)} \sim$ N	$X_{(AC)} \sim$ Gam	$\rho_{(AB)} = 0.25, \rho_{(AC)} = 0.5$	-0.036 (0.005)	0.205 (0.003)	0.205 (<0.001)	94.8 (0.5)
		$X_{(AB)} \sim$ N	$X_{(AC)} \sim$ N	$\rho_{(AB)} = 0.25, \rho_{(AC)} = 0$	-0.036 (0.005)	0.205 (0.003)	0.205 (<0.001)	95.0 (0.5)
		$X_{(AB)} \sim$ N	$X_{(AC)} \sim$ N	$\rho_{(AB)} = \rho_{(AC)} = 0.25$	-0.036 (0.005)	0.205 (0.003)	0.205 (<0.001)	94.9 (0.5)
		$X_{(AB)} \sim$ N	$X_{(AC)} \sim$ N	$\rho_{(AB)} = 0.25, \rho_{(AC)} = 0.5$	-0.036 (0.005)	0.205 (0.003)	0.205 (<0.001)	94.8 (0.5)
	$d_{AC(AB)}$	$X_{(AB)} \sim$ Gam	$X_{(AC)} \sim$ Gam	$\rho_{(AB)} = 0.25, \rho_{(AC)} = 0$	0.113 (0.008)	0.366 (0.006)	0.358 (<0.001)	93.5 (0.6)
		$X_{(AB)} \sim$ Gam	$X_{(AC)} \sim$ Gam	$\rho_{(AB)} = \rho_{(AC)} = 0.25$	0.112 (0.008)	0.366 (0.006)	0.358 (<0.001)	93.5 (0.6)
		$X_{(AB)} \sim$ Gam	$X_{(AC)} \sim$ Gam	$\rho_{(AB)} = 0.25, \rho_{(AC)} = 0.5$	0.112 (0.008)	0.366 (0.006)	0.358 (<0.001)	93.5 (0.5)
		$X_{(AB)} \sim$ Gam	$X_{(AC)} \sim$ N	$\rho_{(AB)} = 0.25, \rho_{(AC)} = 0$	0.113 (0.008)	0.366 (0.006)	0.358 (<0.001)	93.8 (0.5)
		$X_{(AB)} \sim$ Gam	$X_{(AC)} \sim$ N	$\rho_{(AB)} = \rho_{(AC)} = 0.25$	0.112 (0.008)	0.366 (0.006)	0.358 (<0.001)	93.5 (0.6)
		$X_{(AB)} \sim$ Gam	$X_{(AC)} \sim$ N	$\rho_{(AB)} = 0.25, \rho_{(AC)} = 0.5$	0.112 (0.008)	0.366 (0.006)	0.358 (<0.001)	93.7 (0.5)
		$X_{(AB)} \sim$ N	$X_{(AC)} \sim$ Gam	$\rho_{(AB)} = 0.25, \rho_{(AC)} = 0$	0.099 (0.008)	0.367 (0.006)	0.359 (<0.001)	94.2 (0.5)
		$X_{(AB)} \sim$ N	$X_{(AC)} \sim$ Gam	$\rho_{(AB)} = \rho_{(AC)} = 0.25$	0.099 (0.008)	0.367 (0.006)	0.359 (<0.001)	94.3 (0.5)
		$X_{(AB)} \sim$ N	$X_{(AC)} \sim$ Gam	$\rho_{(AB)} = 0.25, \rho_{(AC)} = 0.5$	0.099 (0.008)	0.367 (0.006)	0.359 (<0.001)	94.0 (0.5)
		$X_{(AB)} \sim$ N	$X_{(AC)} \sim$ N	$\rho_{(AB)} = 0.25, \rho_{(AC)} = 0$	0.099 (0.008)	0.367 (0.006)	0.359 (<0.001)	94.1 (0.5)
		$X_{(AB)} \sim$ N	$X_{(AC)} \sim$ N	$\rho_{(AB)} = \rho_{(AC)} = 0.25$	0.099 (0.008)	0.367 (0.006)	0.359 (<0.001)	94.2 (0.5)
		$X_{(AB)} \sim$ N	$X_{(AC)} \sim$ N	$\rho_{(AB)} = 0.25, \rho_{(AC)} = 0.5$	0.099 (0.008)	0.367 (0.006)	0.359 (<0.001)	94.2 (0.5)
	$d_{BC(AB)}$	$X_{(AB)} \sim$ Gam	$X_{(AC)} \sim$ Gam	$\rho_{(AB)} = 0.25, \rho_{(AC)} = 0$	0.148 (0.009)	0.415 (0.007)	0.409 (<0.001)	93.3 (0.6)
		$X_{(AB)} \sim$ Gam	$X_{(AC)} \sim$ Gam	$\rho_{(AB)} = \rho_{(AC)} = 0.25$	0.148 (0.009)	0.416 (0.007)	0.409 (<0.001)	93.2 (0.6)
		$X_{(AB)} \sim$ Gam	$X_{(AC)} \sim$ Gam	$\rho_{(AB)} = 0.25, \rho_{(AC)} = 0.5$	0.148 (0.009)	0.415 (0.007)	0.409 (<0.001)	93.3 (0.6)
		$X_{(AB)} \sim$ Gam	$X_{(AC)} \sim$ N	$\rho_{(AB)} = 0.25, \rho_{(AC)} = 0$	0.148 (0.009)	0.415 (0.007)	0.409 (<0.001)	93.5 (0.6)
		$X_{(AB)} \sim$ Gam	$X_{(AC)} \sim$ N	$\rho_{(AB)} = \rho_{(AC)} = 0.25$	0.148 (0.009)	0.415 (0.007)	0.409 (<0.001)	93.3 (0.6)
		$X_{(AB)} \sim$ Gam	$X_{(AC)} \sim$ N	$\rho_{(AB)} = 0.25, \rho_{(AC)} = 0.5$	0.148 (0.009)	0.415 (0.007)	0.409 (<0.001)	93.2 (0.6)
		$X_{(AB)} \sim$ N	$X_{(AC)} \sim$ Gam	$\rho_{(AB)} = 0.25, \rho_{(AC)} = 0$	0.135 (0.009)	0.414 (0.007)	0.409 (<0.001)	93.2 (0.6)
		$X_{(AB)} \sim$ N	$X_{(AC)} \sim$ Gam	$\rho_{(AB)} = \rho_{(AC)} = 0.25$	0.135 (0.009)	0.414 (0.007)	0.409 (<0.001)	93.2 (0.6)
		$X_{(AB)} \sim$ N	$X_{(AC)} \sim$ Gam	$\rho_{(AB)} = 0.25, \rho_{(AC)} = 0.5$	0.135 (0.009)	0.414 (0.007)	0.409 (<0.001)	93.3 (0.6)
		$X_{(AB)} \sim$ N	$X_{(AC)} \sim$ N	$\rho_{(AB)} = 0.25, \rho_{(AC)} = 0$	0.135 (0.009)	0.414 (0.007)	0.409 (<0.001)	93.3 (0.6)
		$X_{(AB)} \sim$ N	$X_{(AC)} \sim$ N	$\rho_{(AB)} = \rho_{(AC)} = 0.25$	0.135 (0.009)	0.414 (0.007)	0.409 (<0.001)	93.2 (0.6)
		$X_{(AB)} \sim$ N	$X_{(AC)} \sim$ N	$\rho_{(AB)} = 0.25, \rho_{(AC)} = 0.5$	0.135 (0.009)	0.414 (0.007)	0.409 (<0.001)	93.4 (0.6)
	$d_{AB(AC)}$	$X_{(AB)} \sim$ Gam	$X_{(AC)} \sim$ Gam	$\rho_{(AB)} = 0.25, \rho_{(AC)} = 0$	-0.180 (0.008)	0.352 (0.006)	0.352 (<0.001)	92.5 (0.6)
		$X_{(AB)} \sim$ Gam	$X_{(AC)} \sim$ Gam	$\rho_{(AB)} = \rho_{(AC)} = 0.25$	-0.180 (0.008)	0.352 (0.006)	0.352 (<0.001)	92.5 (0.6)
		$X_{(AB)} \sim$ Gam	$X_{(AC)} \sim$ Gam	$\rho_{(AB)} = 0.25, \rho_{(AC)} = 0.5$	-0.179 (0.008)	0.352 (0.006)	0.352 (<0.001)	92.7 (0.6)
		$X_{(AB)} \sim$ Gam	$X_{(AC)} \sim$ N	$\rho_{(AB)} = 0.25, \rho_{(AC)} = 0$	-0.180 (0.008)	0.351 (0.006)	0.352 (<0.001)	92.3 (0.6)
		$X_{(AB)} \sim$ Gam	$X_{(AC)} \sim$ N	$\rho_{(AB)} = \rho_{(AC)} = 0.25$	-0.180 (0.008)	0.352 (0.006)	0.352 (<0.001)	92.3 (0.6)
		$X_{(AB)} \sim$ Gam	$X_{(AC)} \sim$ N	$\rho_{(AB)} = 0.25, \rho_{(AC)} = 0.5$	-0.180 (0.008)	0.352 (0.006)	0.352 (<0.001)	92.3 (0.6)
		$X_{(AB)} \sim$ N	$X_{(AC)} \sim$ Gam	$\rho_{(AB)} = 0.25, \rho_{(AC)} = 0$	-0.167 (0.008)	0.350 (0.006)	0.351 (<0.001)	92.8 (0.6)
		$X_{(AB)} \sim$ N	$X_{(AC)} \sim$ Gam	$\rho_{(AB)} = \rho_{(AC)} = 0.25$	-0.167 (0.008)	0.350 (0.006)	0.351 (<0.001)	92.8 (0.6)
		$X_{(AB)} \sim$ N	$X_{(AC)} \sim$ Gam	$\rho_{(AB)} = 0.25, \rho_{(AC)} = 0.5$	-0.167 (0.008)	0.350 (0.006)	0.351 (<0.001)	92.8 (0.6)
		$X_{(AB)} \sim$ N	$X_{(AC)} \sim$ N	$\rho_{(AB)} = 0.25, \rho_{(AC)} = 0$	-0.167 (0.008)	0.350 (0.006)	0.351 (<0.001)	92.8 (0.6)
		$X_{(AB)} \sim$ N	$X_{(AC)} \sim$ N	$\rho_{(AB)} = \rho_{(AC)} = 0.25$	-0.167 (0.008)	0.350 (0.006)	0.351 (<0.001)	92.7 (0.6)
		$X_{(AB)} \sim$ N	$X_{(AC)} \sim$ N	$\rho_{(AB)} = 0.25, \rho_{(AC)} = 0.5$	-0.167 (0.008)	0.350 (0.006)	0.351 (<0.001)	92.7 (0.6)
	$d_{AC(AC)}$	$X_{(AB)} \sim$ Gam	$X_{(AC)} \sim$ Gam	$\rho_{(AB)} = 0.25, \rho_{(AC)} = 0$	-0.031 (0.005)	0.218 (0.003)	0.210 (<0.001)	94.2 (0.5)
		$X_{(AB)} \sim$ Gam	$X_{(AC)} \sim$ Gam	$\rho_{(AB)} = \rho_{(AC)} = 0.25$	-0.032 (0.005)	0.219 (0.003)	0.210 (<0.001)	94.2 (0.5)
		$X_{(AB)} \sim$ Gam	$X_{(AC)} \sim$ Gam	$\rho_{(AB)} = 0.25, \rho_{(AC)} = 0.5$	-0.032 (0.005)	0.218 (0.003)	0.210 (<0.001)	94.2 (0.5)
		$X_{(AB)} \sim$ Gam	$X_{(AC)} \sim$ N	$\rho_{(AB)} = 0.25, \rho_{(AC)} = 0$	-0.031 (0.005)	0.218 (0.003)	0.210 (<0.001)	94.3 (0.5)
		$X_{(AB)} \sim$ Gam	$X_{(AC)} \sim$ N	$\rho_{(AB)} = \rho_{(AC)} = 0.25$	-0.032 (0.005)	0.218 (0.003)	0.210 (<0.001)	93.9 (0.5)
		$X_{(AB)} \sim$ Gam	$X_{(AC)} \sim$ N	$\rho_{(AB)} = 0.25, \rho_{(AC)} = 0.5$	-0.032 (0.005)	0.218 (0.003)	0.210 (<0.001)	93.9 (0.5)
		$X_{(AB)} \sim$ N	$X_{(AC)} \sim$ Gam	$\rho_{(AB)} = 0.25, \rho_{(AC)} = 0$	-0.031 (0.005)	0.218 (0.003)	0.210 (<0.001)	94.2 (0.5)
		$X_{(AB)} \sim$ N	$X_{(AC)} \sim$ Gam	$\rho_{(AB)} = \rho_{(AC)} = 0.25$	-0.032 (0.005)	0.219 (0.003)	0.210 (<0.001)	94.0 (0.5)
		$X_{(AB)} \sim$ N	$X_{(AC)} \sim$ Gam	$\rho_{(AB)} = 0.25, \rho_{(AC)} = 0.5$	-0.032 (0.005)	0.218 (0.003)	0.210 (<0.001)	94.2 (0.5)
		$X_{(AB)} \sim$ N	$X_{(AC)} \sim$ N	$\rho_{(AB)} = 0.25, \rho_{(AC)} = 0$	-0.031 (0.005)	0.218 (0.003)	0.210 (<0.001)	94.2 (0.5)
		$X_{(AB)} \sim$ N	$X_{(AC)} \sim$ N	$\rho_{(AB)} = \rho_{(AC)} = 0.25$	-0.032 (0.005)	0.218 (0.003)	0.210 (<0.001)	94.0 (0.5)
		$X_{(AB)} \sim$ N	$X_{(AC)} \sim$ N	$\rho_{(AB)} = 0.25, \rho_{(AC)} = 0.5$	-0.031 (0.005)	0.218 (0.003)	0.210 (<0.001)	93.8 (0.5)
	$d_{BC(AC)}$	$X_{(AB)} \sim$ Gam	$X_{(AC)} \sim$ Gam	$\rho_{(AB)} = 0.25, \rho_{(AC)} = 0$	0.148 (0.009)	0.415 (0.007)	0.409 (<0.001)	93.3 (0.6)
		$X_{(AB)} \sim$ Gam	$X_{(AC)} \sim$ Gam	$\rho_{(AB)} = \rho_{(AC)} = 0.25$	0.148 (0.009)	0.416 (0.007)	0.409 (<0.001)	93.2 (0.6)
		$X_{(AB)} \sim$ Gam	$X_{(AC)} \sim$ Gam	$\rho_{(AB)} = 0.25, \rho_{(AC)} = 0.5$	0.148 (0.009)	0.415 (0.007)	0.409 (<0.001)	93.3 (0.6)
		$X_{(AB)} \sim$ Gam	$X_{(AC)} \sim$ N	$\rho_{(AB)} = 0.25, \rho_{(AC)} = 0$	0.148 (0.009)	0.415 (0.007)	0.409 (<0.001)	93.5 (0.6)

TABLE B12 (continued)

Method	Contrast	Scenario			Bias	Empirical SE	Model SE	Coverage
		$X_{(AB)} \sim$ Gam	$X_{(AC)} \sim$ N	$\rho_{(AB)} = \rho_{(AC)} = 0.25$	0.148 (0.009)	0.415 (0.007)	0.409 (<0.001)	93.3 (0.6)
		$X_{(AB)} \sim$ Gam	$X_{(AC)} \sim$ N	$\rho_{(AB)} = 0.25, \rho_{(AC)} = 0.5$	0.148 (0.009)	0.415 (0.007)	0.409 (<0.001)	93.2 (0.6)
		$X_{(AB)} \sim$ N	$X_{(AC)} \sim$ Gam	$\rho_{(AB)} = 0.25, \rho_{(AC)} = 0$	0.135 (0.009)	0.414 (0.007)	0.409 (<0.001)	93.2 (0.6)
		$X_{(AB)} \sim$ N	$X_{(AC)} \sim$ Gam	$\rho_{(AB)} = \rho_{(AC)} = 0.25$	0.135 (0.009)	0.414 (0.007)	0.409 (<0.001)	93.2 (0.6)
		$X_{(AB)} \sim$ N	$X_{(AC)} \sim$ Gam	$\rho_{(AB)} = 0.25, \rho_{(AC)} = 0.5$	0.135 (0.009)	0.414 (0.007)	0.409 (<0.001)	93.3 (0.6)
		$X_{(AB)} \sim$ N	$X_{(AC)} \sim$ N	$\rho_{(AB)} = 0.25, \rho_{(AC)} = 0$	0.135 (0.009)	0.414 (0.007)	0.409 (<0.001)	93.3 (0.6)
		$X_{(AB)} \sim$ N	$X_{(AC)} \sim$ N	$\rho_{(AB)} = \rho_{(AC)} = 0.25$	0.135 (0.009)	0.414 (0.007)	0.409 (<0.001)	93.2 (0.6)
		$X_{(AB)} \sim$ N	$X_{(AC)} \sim$ N	$\rho_{(AB)} = 0.25, \rho_{(AC)} = 0.5$	0.135 (0.009)	0.414 (0.007)	0.409 (<0.001)	93.4 (0.6)
STC	$d_{AB(AC)}$	$X_{(AB)} \sim$ Gam	$X_{(AC)} \sim$ Gam	$\rho_{(AB)} = 0.25, \rho_{(AC)} = 0$	-0.159 (0.008)	0.344 (0.005)	0.348 (<0.001)	94.0 (0.5)
		$X_{(AB)} \sim$ Gam	$X_{(AC)} \sim$ Gam	$\rho_{(AB)} = \rho_{(AC)} = 0.25$	-0.159 (0.008)	0.344 (0.005)	0.348 (<0.001)	94.0 (0.5)
		$X_{(AB)} \sim$ Gam	$X_{(AC)} \sim$ Gam	$\rho_{(AB)} = 0.25, \rho_{(AC)} = 0.5$	-0.159 (0.008)	0.344 (0.005)	0.348 (<0.001)	94.0 (0.5)
		$X_{(AB)} \sim$ Gam	$X_{(AC)} \sim$ N	$\rho_{(AB)} = 0.25, \rho_{(AC)} = 0$	-0.159 (0.008)	0.344 (0.005)	0.348 (<0.001)	94.0 (0.5)
		$X_{(AB)} \sim$ Gam	$X_{(AC)} \sim$ N	$\rho_{(AB)} = \rho_{(AC)} = 0.25$	-0.159 (0.008)	0.344 (0.005)	0.348 (<0.001)	94.0 (0.5)
		$X_{(AB)} \sim$ Gam	$X_{(AC)} \sim$ N	$\rho_{(AB)} = 0.25, \rho_{(AC)} = 0.5$	-0.159 (0.008)	0.344 (0.005)	0.348 (<0.001)	94.0 (0.5)
		$X_{(AB)} \sim$ N	$X_{(AC)} \sim$ Gam	$\rho_{(AB)} = 0.25, \rho_{(AC)} = 0$	-0.151 (0.008)	0.344 (0.005)	0.348 (<0.001)	94.2 (0.5)
		$X_{(AB)} \sim$ N	$X_{(AC)} \sim$ Gam	$\rho_{(AB)} = \rho_{(AC)} = 0.25$	-0.151 (0.008)	0.344 (0.005)	0.348 (<0.001)	94.2 (0.5)
		$X_{(AB)} \sim$ N	$X_{(AC)} \sim$ Gam	$\rho_{(AB)} = 0.25, \rho_{(AC)} = 0.5$	-0.151 (0.008)	0.344 (0.005)	0.348 (<0.001)	94.2 (0.5)
		$X_{(AB)} \sim$ N	$X_{(AC)} \sim$ N	$\rho_{(AB)} = 0.25, \rho_{(AC)} = 0$	-0.151 (0.008)	0.344 (0.005)	0.348 (<0.001)	94.2 (0.5)
		$X_{(AB)} \sim$ N	$X_{(AC)} \sim$ N	$\rho_{(AB)} = \rho_{(AC)} = 0.25$	-0.151 (0.008)	0.344 (0.005)	0.348 (<0.001)	94.2 (0.5)
		$X_{(AB)} \sim$ N	$X_{(AC)} \sim$ N	$\rho_{(AB)} = 0.25, \rho_{(AC)} = 0.5$	-0.151 (0.008)	0.344 (0.005)	0.348 (<0.001)	94.2 (0.5)
	$d_{BC(AC)}$	$X_{(AB)} \sim$ Gam	$X_{(AC)} \sim$ Gam	$\rho_{(AB)} = 0.25, \rho_{(AC)} = 0$	0.144 (0.009)	0.408 (0.006)	0.406 (<0.001)	94.0 (0.5)
		$X_{(AB)} \sim$ Gam	$X_{(AC)} \sim$ Gam	$\rho_{(AB)} = \rho_{(AC)} = 0.25$	0.143 (0.009)	0.408 (0.006)	0.406 (<0.001)	93.8 (0.5)
		$X_{(AB)} \sim$ Gam	$X_{(AC)} \sim$ Gam	$\rho_{(AB)} = 0.25, \rho_{(AC)} = 0.5$	0.143 (0.009)	0.408 (0.006)	0.406 (<0.001)	93.8 (0.5)
		$X_{(AB)} \sim$ Gam	$X_{(AC)} \sim$ N	$\rho_{(AB)} = 0.25, \rho_{(AC)} = 0$	0.144 (0.009)	0.408 (0.006)	0.406 (<0.001)	94.0 (0.5)
		$X_{(AB)} \sim$ Gam	$X_{(AC)} \sim$ N	$\rho_{(AB)} = \rho_{(AC)} = 0.25$	0.143 (0.009)	0.408 (0.006)	0.406 (<0.001)	93.9 (0.5)
		$X_{(AB)} \sim$ Gam	$X_{(AC)} \sim$ N	$\rho_{(AB)} = 0.25, \rho_{(AC)} = 0.5$	0.144 (0.009)	0.408 (0.006)	0.406 (<0.001)	93.8 (0.5)
		$X_{(AB)} \sim$ N	$X_{(AC)} \sim$ Gam	$\rho_{(AB)} = 0.25, \rho_{(AC)} = 0$	0.136 (0.009)	0.408 (0.006)	0.406 (<0.001)	93.8 (0.5)
		$X_{(AB)} \sim$ N	$X_{(AC)} \sim$ Gam	$\rho_{(AB)} = \rho_{(AC)} = 0.25$	0.136 (0.009)	0.408 (0.006)	0.406 (<0.001)	93.9 (0.5)
		$X_{(AB)} \sim$ N	$X_{(AC)} \sim$ Gam	$\rho_{(AB)} = 0.25, \rho_{(AC)} = 0.5$	0.136 (0.009)	0.408 (0.006)	0.406 (<0.001)	93.8 (0.5)
		$X_{(AB)} \sim$ N	$X_{(AC)} \sim$ N	$\rho_{(AB)} = 0.25, \rho_{(AC)} = 0$	0.136 (0.009)	0.408 (0.006)	0.406 (<0.001)	93.8 (0.5)
		$X_{(AB)} \sim$ N	$X_{(AC)} \sim$ N	$\rho_{(AB)} = \rho_{(AC)} = 0.25$	0.136 (0.009)	0.408 (0.006)	0.406 (<0.001)	93.7 (0.5)
		$X_{(AB)} \sim$ N	$X_{(AC)} \sim$ N	$\rho_{(AB)} = 0.25, \rho_{(AC)} = 0.5$	0.136 (0.009)	0.408 (0.006)	0.406 (<0.001)	93.8 (0.5)
MAIC	$d_{AB(AC)}$	$X_{(AB)} \sim$ Gam	$X_{(AC)} \sim$ Gam	$\rho_{(AB)} = 0.25, \rho_{(AC)} = 0$	-0.165 (0.009)	0.399 (0.006)	0.411 (<0.001)	91.7 (0.6)
		$X_{(AB)} \sim$ Gam	$X_{(AC)} \sim$ Gam	$\rho_{(AB)} = \rho_{(AC)} = 0.25$	-0.165 (0.009)	0.399 (0.006)	0.411 (<0.001)	91.7 (0.6)
		$X_{(AB)} \sim$ Gam	$X_{(AC)} \sim$ Gam	$\rho_{(AB)} = 0.25, \rho_{(AC)} = 0.5$	-0.165 (0.009)	0.399 (0.006)	0.411 (<0.001)	91.7 (0.6)
		$X_{(AB)} \sim$ Gam	$X_{(AC)} \sim$ N	$\rho_{(AB)} = 0.25, \rho_{(AC)} = 0$	-0.165 (0.009)	0.399 (0.006)	0.411 (<0.001)	91.7 (0.6)
		$X_{(AB)} \sim$ Gam	$X_{(AC)} \sim$ N	$\rho_{(AB)} = \rho_{(AC)} = 0.25$	-0.165 (0.009)	0.399 (0.006)	0.411 (<0.001)	91.7 (0.6)
		$X_{(AB)} \sim$ Gam	$X_{(AC)} \sim$ N	$\rho_{(AB)} = 0.25, \rho_{(AC)} = 0.5$	-0.165 (0.009)	0.399 (0.006)	0.411 (<0.001)	91.7 (0.6)
		$X_{(AB)} \sim$ N	$X_{(AC)} \sim$ Gam	$\rho_{(AB)} = 0.25, \rho_{(AC)} = 0$	-0.176 (0.010)	0.442 (0.007)	0.448 (0.001)	91.2 (0.6)
		$X_{(AB)} \sim$ N	$X_{(AC)} \sim$ Gam	$\rho_{(AB)} = \rho_{(AC)} = 0.25$	-0.176 (0.010)	0.442 (0.007)	0.448 (0.001)	91.2 (0.6)
		$X_{(AB)} \sim$ N	$X_{(AC)} \sim$ Gam	$\rho_{(AB)} = 0.25, \rho_{(AC)} = 0.5$	-0.176 (0.010)	0.442 (0.007)	0.448 (0.001)	91.2 (0.6)
		$X_{(AB)} \sim$ N	$X_{(AC)} \sim$ N	$\rho_{(AB)} = 0.25, \rho_{(AC)} = 0$	-0.176 (0.010)	0.441 (0.007)	0.447 (0.001)	91.3 (0.6)
		$X_{(AB)} \sim$ N	$X_{(AC)} \sim$ N	$\rho_{(AB)} = \rho_{(AC)} = 0.25$	-0.176 (0.010)	0.441 (0.007)	0.447 (0.001)	91.3 (0.6)
		$X_{(AB)} \sim$ N	$X_{(AC)} \sim$ N	$\rho_{(AB)} = 0.25, \rho_{(AC)} = 0.5$	-0.176 (0.010)	0.441 (0.007)	0.447 (0.001)	91.3 (0.6)
	$d_{BC(AC)}$	$X_{(AB)} \sim$ Gam	$X_{(AC)} \sim$ Gam	$\rho_{(AB)} = 0.25, \rho_{(AC)} = 0$	0.150 (0.010)	0.453 (0.007)	0.461 (<0.001)	94.1 (0.5)
		$X_{(AB)} \sim$ Gam	$X_{(AC)} \sim$ Gam	$\rho_{(AB)} = \rho_{(AC)} = 0.25$	0.150 (0.010)	0.453 (0.007)	0.461 (<0.001)	94.0 (0.5)
		$X_{(AB)} \sim$ Gam	$X_{(AC)} \sim$ Gam	$\rho_{(AB)} = 0.25, \rho_{(AC)} = 0.5$	0.150 (0.010)	0.453 (0.007)	0.461 (<0.001)	94.0 (0.5)
		$X_{(AB)} \sim$ Gam	$X_{(AC)} \sim$ N	$\rho_{(AB)} = 0.25, \rho_{(AC)} = 0$	0.150 (0.010)	0.453 (0.007)	0.461 (<0.001)	94.2 (0.5)
		$X_{(AB)} \sim$ Gam	$X_{(AC)} \sim$ N	$\rho_{(AB)} = \rho_{(AC)} = 0.25$	0.150 (0.010)	0.453 (0.007)	0.461 (<0.001)	94.2 (0.5)
		$X_{(AB)} \sim$ Gam	$X_{(AC)} \sim$ N	$\rho_{(AB)} = 0.25, \rho_{(AC)} = 0.5$	0.150 (0.010)	0.453 (0.007)	0.461 (<0.001)	94.1 (0.5)
		$X_{(AB)} \sim$ N	$X_{(AC)} \sim$ Gam	$\rho_{(AB)} = 0.25, \rho_{(AC)} = 0$	0.161 (0.011)	0.489 (0.008)	0.494 (0.001)	93.7 (0.5)
		$X_{(AB)} \sim$ N	$X_{(AC)} \sim$ Gam	$\rho_{(AB)} = \rho_{(AC)} = 0.25$	0.161 (0.011)	0.489 (0.008)	0.494 (0.001)	93.8 (0.5)
		$X_{(AB)} \sim$ N	$X_{(AC)} \sim$ Gam	$\rho_{(AB)} = 0.25, \rho_{(AC)} = 0.5$	0.161 (0.011)	0.490 (0.008)	0.494 (0.001)	93.8 (0.5)
		$X_{(AB)} \sim$ N	$X_{(AC)} \sim$ N	$\rho_{(AB)} = 0.25, \rho_{(AC)} = 0$	0.161 (0.011)	0.489 (0.008)	0.494 (0.001)	93.5 (0.5)
		$X_{(AB)} \sim$ N	$X_{(AC)} \sim$ N	$\rho_{(AB)} = \rho_{(AC)} = 0.25$	0.161 (0.011)	0.489 (0.008)	0.494 (0.001)	93.8 (0.5)
		$X_{(AB)} \sim$ N	$X_{(AC)} \sim$ N	$\rho_{(AB)} = 0.25, \rho_{(AC)} = 0.5$	0.161 (0.011)	0.489 (0.008)	0.494 (0.001)	93.5 (0.5)
Bucher	$d_{AB(AC)}$	$X_{(AB)} \sim$ Gam	$X_{(AC)} \sim$ Gam	$\rho_{(AB)} = 0.25, \rho_{(AC)} = 0$	-0.318 (0.004)	0.199 (0.003)	0.202 (<0.001)	65.5 (1.1)
		$X_{(AB)} \sim$ Gam	$X_{(AC)} \sim$ Gam	$\rho_{(AB)} = \rho_{(AC)} = 0.25$	-0.318 (0.004)	0.199 (0.003)	0.202 (<0.001)	65.5 (1.1)
		$X_{(AB)} \sim$ Gam	$X_{(AC)} \sim$ Gam	$\rho_{(AB)} = 0.25, \rho_{(AC)} = 0.5$	-0.318 (0.004)	0.199 (0.003)	0.202 (<0.001)	65.5 (1.1)
		$X_{(AB)} \sim$ Gam	$X_{(AC)} \sim$ N	$\rho_{(AB)} = 0.25, \rho_{(AC)} = 0$	-0.318 (0.004)	0.199 (0.003)	0.202 (<0.001)	65.5 (1.1)
		$X_{(AB)} \sim$ Gam	$X_{(AC)} \sim$ N	$\rho_{(AB)} = \rho_{(AC)} = 0.25$	-0.318 (0.004)	0.199 (0.003)	0.202 (<0.001)	65.5 (1.1)
		$X_{(AB)} \sim$ Gam	$X_{(AC)} \sim$ N	$\rho_{(AB)} = 0.25, \rho_{(AC)} = 0.5$	-0.318 (0.004)	0.199 (0.003)	0.202 (<0.001)	65.5 (1.1)
		$X_{(AB)} \sim$ N	$X_{(AC)} \sim$ Gam	$\rho_{(AB)} = 0.25, \rho_{(AC)} = 0$	-0.318 (0.004)	0.199 (0.003)	0.202 (<0.001)	65.3 (1.1)
		$X_{(AB)} \sim$ N	$X_{(AC)} \sim$ Gam	$\rho_{(AB)} = \rho_{(AC)} = 0.25$	-0.318 (0.004)	0.199 (0.003)	0.202 (<0.001)	65.3 (1.1)
		$X_{(AB)} \sim$ N	$X_{(AC)} \sim$ Gam	$\rho_{(AB)} = 0.25, \rho_{(AC)} = 0.5$	-0.318 (0.004)	0.199 (0.003)	0.202 (<0.001)	65.3 (1.1)
		$X_{(AB)} \sim$ N	$X_{(AC)} \sim$ N	$\rho_{(AB)} = 0.25, \rho_{(AC)} = 0$	-0.318 (0.004)	0.199 (0.003)	0.202 (<0.001)	65.3 (1.1)

TABLE B12 (continued)

Method	Contrast	Scenario			Bias	Empirical SE	Model SE	Coverage
		$X_{(AB)} \sim$ N	$X_{(AC)} \sim$ N	$\rho_{(AB)} = \rho_{(AC)} = 0.25$	-0.318 (0.004)	0.199 (0.003)	0.202 (<0.001)	65.3 (1.1)
		$X_{(AB)} \sim$ N	$X_{(AC)} \sim$ N	$\rho_{(AB)} = 0.25, \rho_{(AC)} = 0.5$	-0.318 (0.004)	0.199 (0.003)	0.202 (<0.001)	65.3 (1.1)
	$d_{BC(AC)}$	$X_{(AB)} \sim$ Gam	$X_{(AC)} \sim$ Gam	$\rho_{(AB)} = 0.25, \rho_{(AC)} = 0$	0.303 (0.007)	0.295 (0.005)	0.291 (<0.001)	81.8 (0.9)
		$X_{(AB)} \sim$ Gam	$X_{(AC)} \sim$ Gam	$\rho_{(AB)} = \rho_{(AC)} = 0.25$	0.303 (0.007)	0.296 (0.005)	0.291 (<0.001)	81.8 (0.9)
		$X_{(AB)} \sim$ Gam	$X_{(AC)} \sim$ Gam	$\rho_{(AB)} = 0.25, \rho_{(AC)} = 0.5$	0.303 (0.007)	0.296 (0.005)	0.291 (<0.001)	81.7 (0.9)
		$X_{(AB)} \sim$ Gam	$X_{(AC)} \sim$ N	$\rho_{(AB)} = 0.25, \rho_{(AC)} = 0$	0.304 (0.007)	0.295 (0.005)	0.291 (<0.001)	81.7 (0.9)
		$X_{(AB)} \sim$ Gam	$X_{(AC)} \sim$ N	$\rho_{(AB)} = \rho_{(AC)} = 0.25$	0.303 (0.007)	0.296 (0.005)	0.291 (<0.001)	81.7 (0.9)
		$X_{(AB)} \sim$ Gam	$X_{(AC)} \sim$ N	$\rho_{(AB)} = 0.25, \rho_{(AC)} = 0.5$	0.303 (0.007)	0.295 (0.005)	0.291 (<0.001)	81.6 (0.9)
		$X_{(AB)} \sim$ N	$X_{(AC)} \sim$ Gam	$\rho_{(AB)} = 0.25, \rho_{(AC)} = 0$	0.303 (0.007)	0.295 (0.005)	0.291 (<0.001)	81.8 (0.9)
		$X_{(AB)} \sim$ N	$X_{(AC)} \sim$ Gam	$\rho_{(AB)} = \rho_{(AC)} = 0.25$	0.303 (0.007)	0.296 (0.005)	0.291 (<0.001)	81.8 (0.9)
		$X_{(AB)} \sim$ N	$X_{(AC)} \sim$ Gam	$\rho_{(AB)} = 0.25, \rho_{(AC)} = 0.5$	0.303 (0.007)	0.296 (0.005)	0.291 (<0.001)	81.8 (0.9)
		$X_{(AB)} \sim$ N	$X_{(AC)} \sim$ N	$\rho_{(AB)} = 0.25, \rho_{(AC)} = 0$	0.304 (0.007)	0.295 (0.005)	0.291 (<0.001)	81.8 (0.9)
		$X_{(AB)} \sim$ N	$X_{(AC)} \sim$ N	$\rho_{(AB)} = \rho_{(AC)} = 0.25$	0.303 (0.007)	0.295 (0.005)	0.291 (<0.001)	81.8 (0.9)
		$X_{(AB)} \sim$ N	$X_{(AC)} \sim$ N	$\rho_{(AB)} = 0.25, \rho_{(AC)} = 0.5$	0.303 (0.007)	0.295 (0.005)	0.291 (<0.001)	81.8 (0.9)
